# Integrative taxonomy of a new and highly-diverse genus of onchidiid slugs from the Coral Triangle (Gastropoda, Pulmonata, Onchidiidae)

**DOI:** 10.3897/zookeys.763.21252

**Published:** 2018-06-04

**Authors:** Tricia C. Goulding, Munawar Khalil, Shau Hwai Tan, Benoît Dayrat

**Affiliations:** 1 Department of Biology, Pennsylvania State University, University Park, PA 16802, USA; 2 Current address: Bernice Pauahi Bishop Museum, 1525 Bernice St, Honolulu, HI 96817; 3 Department of Marine Science, Universitas Malikussaleh. Reuleut Main Campus, Kecamatan Muara Batu, North Aceh, Aceh, 24355, Indonesia; 4 Marine Science Laboratory, School of Biological Sciences, and Centre for Marine and Coastal Studies, Universiti Sains Malaysia, 11800 Penang, Malaysia

**Keywords:** Euthyneura, Indo-West Pacific, marine biogeography, phylogeography, systematics

## Abstract

A new genus of onchidiid slugs, *Wallaconchis* Goulding & Dayrat, **gen. n.**, is described, including ten species. Five species were previously described but known only from the type material: *Wallaconchis ater* (Lesson, 1830), *W. graniferum* (Semper, 1880), *W. nangkauriense* (Plate, 1893), *W. buetschlii* (Stantschinsky, 1907), and *W. gracile* (Stantschinsky, 1907), all of which were originally classified in *Onchidium* Buchannan, 1800. Many new records are provided for these five species, which greatly expand their known geographic distributions. Five species are new: *Wallaconchis achleitneri* Goulding, **sp. n.**, *W. comendadori* Goulding & Dayrat, **sp. n.**, *W. melanesiensis* Goulding & Dayrat, **sp. n.**, *W. sinanui* Goulding & Dayrat, **sp. n.**, and *W. uncinus* Goulding & Dayrat, **sp. n.** Nine of the ten *Wallaconchis* species are found in the Coral Triangle (eastern Indonesia and the Philippines). Sympatry is high, with up to six species found on the island of Bohol (Philippines) and eight species overlapping in northern Sulawesi (Indonesia). *Wallaconchis* is distinguished from other onchidiids by its bright dorsal colors (red, yellow, orange) but those are extremely variable and not useful for specific identification. Internally, the reproductive system can be used to identify all *Wallaconchis* species. The copulatory organs of *Wallaconchis* species are especially diverse compared to other onchidiid genera, and the possible role of reproductive incompatibility in species diversification is discussed. All specimens examined were freshly collected for the purpose of a worldwide revision of the Onchidiidae Rafinesque, 1815. The species are well delineated using DNA sequences and comparative anatomy. Mitochondrial DNA analysis yields thirteen molecular units separated by a large barcode gap, while nuclear DNA yields nine units. By integrating nuclear DNA and mitochondrial DNA with morphology, ten species are recognized. The natural history of each species (e.g., the microhabitat where they are found) is also documented. Nomenclature is addressed thoroughly (the types of all onchidiid species were examined, lectotypes were designated when needed, *nomina dubia* are discussed). Morphological characters, transitions to new microhabitats, and diversification processes are discussed in the context of a robust molecular phylogeny.

## Introduction

The Onchidiidae is a group of shell-less, marine, air-breathing slugs that live in intertidal habitats worldwide. Most of the diversity is found in mangroves of South-East Asia, where onchidiids have diversified into several genera, such as *Onchidium* Buchannan, 1800, *Peronina* Plate, 1893, *Platevindex* Baker, 1938, and *Melayonchis* Dayrat & Goulding, 2017. The genus *Peronia* Fleming, 1822, which can easily be identified externally by branched gills on the dorsal notum, has diversified in the rocky intertidal across the Indo-West Pacific. However, for decades the diversity of onchidiid slugs has remained poorly known for a variety of reasons: mangroves have rarely been explored; preserved slugs can hardly be distinguished externally; and taxonomists have avoided the overwhelming nomenclature (Dayrat 2009). As a result, most species could not be reliably identified even though many species are geographically widespread and common.

The Dayrat lab is currently engaged in a global revision of the Onchidiidae, based on an integrative approach involving extensive fieldwork (sampling thousands of individuals at more than 300 stations worldwide), natural history observations, comparative anatomy, and DNA sequencing. An important component of integrative taxonomy is sound nomenclature; this revision has required the examination of the type material of all onchidiid species, the detailed analysis of all species descriptions, and the re-evaluation of the nomenclatural status of every genus- and species-group name. First, monophyletic genera had to be delineated, and now each genus is being revised separately (Dayrat et al. 2016, 2017; Dayrat and Goulding 2017; Goulding et al. in press).

Here, a new genus, *Wallaconchis* Goulding & Dayrat, gen. n., is described from the Indo-West Pacific, with a geographic distribution from the Andaman Islands (Bay of Bengal) to the Philippines, Papua New Guinea, Vanuatu, and Queensland. Phylogenetic analyses show that the monophyly of this new genus is strongly-supported and that it includes ten species. A new genus name is needed because no existing genus name applies to this monophyletic group. This new genus is characterized by a unique combination of morphological characters found in no other onchidiid genus: no dorsal gills, intestinal loops of type I, no rectal gland, and no accessory penial gland. As is often the case in other genera, all ten *Wallaconchis* species are cryptic externally but each is characterized by a distinctive internal anatomy.

Five of the ten species described here are new: *Wallaconchis achleitneri* Goulding, sp. n., *W. comendadori* Goulding & Dayrat, sp. n., *W. melanesiensis* Goulding & Dayrat, sp. n., *W. sinanui* Goulding & Dayrat, sp. n., and *W. uncinus* Goulding & Dayrat, sp. n. The five other species were known only from the type material and are re-described here in detail for the first time with fresh material collected from many locations: *Wallaconchis ater* (Lesson, 1830), *W. graniferum* (Semper, 1880), *W. nangkauriense* (Plate, 1893), *W. buetschlii* (Stantschinsky, 1907), and *W. gracile* (Stantschinsky, 1907), all of which were originally classified in *Onchidium*.

More than 230 non-type specimens are examined in the present study, all of which were collected by the authors in the past few years. DNA sequences are provided for 169 of these specimens. In addition to the description of five new species, dozens of new records are provided for the species with existing names.

The name *Wallaconchis* honors Alfred Russel Wallace, the famous naturalist who traveled extensively in the Indo-Malay Archipelago, the core of the geographic distribution of this new genus. Indeed, nine of the ten *Wallaconchis* species are found within the boundaries of the Coral Triangle, in the waters of eastern Indonesia and the Philippines. Four of these species are distributed outside the Coral Triangle as well, in the Andaman Islands, southern China, northern Queensland, and Vanuatu. In addition, one species is endemic to the central coast of Queensland, Australia.

The five *Wallaconchis* species to which existing species names are applied, were all originally described based on preserved material. Previous authors did not have access to live animals. The new collections, studied here, have revealed that many *Wallaconchis* species exhibit bright dorsal colors, such as red, yellow, and orange, which are unusual in the Onchidiidae. Also, the color of the dorsal notum is highly variable intra-specifically and color variation overlaps between the species. As a result, it remains extremely challenging to identify *Wallaconchis* species in the field, especially because they are commonly found at the exact same spots (up to six species together in a few square meters).

Contrary to other onchidiid genera, which usually are restricted to one or two microhabitats (for instance, *Peronina* species live on soft and deep mud saturated with water), *Wallaconchis* species live on a variety of different microhabitats: coral rubble, which is common in coastal mangroves of the Coral Triangle, fine sand, coarse sand, sandy mud, firm mud, as well as rocks covered with algae. Photographs of microhabitats and live specimens in the field are provided in each species description. In the general discussion, microhabitats are mapped onto the *Wallaconchis* phylogeny and evolutionary transitions in microhabitat are discussed in relation to species diversification.

## Materials and methods

### Collecting

Nearly all specimens examined here were newly collected, which provided fresh material for DNA sequencing and invaluable natural history observations in the field. Specimens were individually numbered and photographed in the field, providing the source for microhabitat data of each species. At each site, individuals were chosen to represent color, size, and microhabitat variation. A piece of tissue was cut from most specimens for DNA extraction, the rest of the specimen being relaxed and preserved for comparative anatomy. Some specimens were also preserved directly in 95% ethanol. Thanks to our numbering of individuals in the field, each DNA sequence can be matched to a particular preserved specimen and the corresponding field photographs. Specimens preserved for dissection were relaxed in magnesium chloride for approximately one day and then preserved in 70% ethanol. Samples from Sumatra, Sulawesi, and Queensland were fixed in formalin prior to being transferred into ethanol. Collections were made by Tricia Goulding and party in Australia, Benoît Dayrat and party in India and in the Philippines, and Munawar Khalil and party in Indonesia. Specimens were collected by hand at low tide. GPS coordinates are available for all stations sampled by the authors and each site is labelled with a unique station number (e.g., st 100). The only specimens that were not collected by the authors are the types of existing species and the specimens collected in Vanuatu and Papua New Guinea during expeditions organized by Dr. Philippe Bouchet (Muséum national d’histoire naturelle, Paris, France).

### Specimens

All available types of Onchidiidae were examined. In total, 231 non-type specimens were examined and sequenced for this study. Mitochondrial COI sequences are provided here for169 individuals and two additional mitochondrial loci (12S and 16S) and three additional nuclear loci (ITS1, ITS2 and 28S) were sequenced for a subsample of individuals. Individuals were subsampled to represent the highest COI genetic divergence within each species, as well as a broad range of geographic localities. Individual DNA extraction numbers are indicated on phylogenetic trees, in the lists of material examined, as well as in figure captions (DNA extraction numbers are included in square brackets). Animal sizes are indicated as length/width in millimeters. All specimens freshly collected by the authors (i.e., all specimens included here except for the types of existing species and the MNHN material from Vanuatu and Papua New Guinea) were deposited as vouchers in institutions in the countries where they were collected.

**Museum abbreviations are**:

**BNHS** Bombay Natural History Society, Mumbai, India

**MNHN** Muséum national d’histoire naturelle, Paris, France

**MTQ** Museum of Tropical Queensland, Townsville, Queensland, Australia

**PNM** National Museum of the Philippines, Manila, Philippines

**SMF** Naturmuseum Senckenberg, Frankfurt-am-Main, Germany

**UMIZ** Universitas Malikussaleh, North Aceh, Sumatra, Indonesia

**ZMB** Zoologisches Museum, Berlin, Germany

**ZMUC** Zoological Museum University of Copenhagen, Denmark

### Anatomical preparations and descriptions

Anatomical observations were made under a dissecting microscope and drawn with a camera lucida. Radulae and male reproductive organs were prepared for scanning electron microscopy (SEM). Radulae were cleaned in 10% NaOH for a week, rinsed in distilled water, briefly cleaned in an ultrasonic water bath (less than a minute), sputter-coated with gold-palladium and examined by SEM. Soft parts were dehydrated in ethanol and critical point-dried before coating. The anatomy of *W. sinanui*, the type species, is fully detailed. The written description of the many anatomical features that are virtually identical between species (nervous system, heart, etc.) is given only for the type species to avoid repeating the information ten times. So, any feature that is only mentioned in *W. sinanui* is identical in the nine other species.

### DNA extraction and PCR amplification

DNA was extracted using the phenol-chloroform extraction protocol with cetyltrimethyl-ammonium bromide (CTAB). Three mitochondrial and three nuclear loci were amplified with PCR. Portions of three mitochondrial (COI, 16S, and 12S) loci were amplified using the following primers (all 5’-3’): COIF GGT CAA CAA ATC ATA AAG ATA TTG G, and COIR TAA ACT TCA GGG TGA CCA AAR AAY CA (Folmer et al. 1994); 16Sar CGC CTG TTT ATC AAA AAC AT (Palumbi 1996), and 16S 972R CCG GTC TGA ACT CAG ATC ATG T (Dayrat et al. 2011); 12sai-L AAA CTA GGA TTA GAT ACC CTA TTA T, and 12SB-H GAG GGTGAC GGG CGG TGT GT (Palumbi 1996). The nuclear ITS1 and ITS2 regions (including part of the 5.8S region), as well as part of the 28S region were amplified using the following primers (all 5’-3’): ITS1-18S TAACAAGGTTTCCGTATGTGAA, ITS1-5.8S GCGTTCTTCATCGATGC (Armbruster et al. 2000), ITS2-LSU1 CTAGCTGCGAGAATTAATGTGA, ITS2-LSU-3 ACTTTCCCTCACGGTACTTG (Wade and Mordan 2000), 28SC1 ACC CGC TGA ATT TAA GCA T (Hassouna et al. 1984), and 28SD3 GAC GAT CGA TTT GCA CGT CA (Vonnemann et al. 2005). The PCR reactions for COI and 16S were 25 μl reactions containing 15.8 μl of water, 2.5 μl of 10X PCR Buffer, 1.5 μl of 25 mM MgCl_2_, 0.5 μl of each 10 μM primer, 2 μl of dNTP mixture, 0.2 μl (1 unit) of TaKaRa Taq (Clontech, Cat No. R001A), 1 μl of 20 ng/μl template DNA, and 1 μl of 100X BSA (Bovine Serum Albumin). The PCR reaction for 12S differed by reducing the water to 14.3 μl, and replacing the MgCl_2_ and BSA with 4 μl of Q solution (QIAGEN, ID: 201203). The PCR reaction for 28S was the same as for 12S, except that the dNTP mixture was reduced to 1 μl, the Q solution increased to 5 μl, and the amount of template DNA reduced to 0.5 μl. The PCR reactions for ITS1 and ITS2 used the reagents in the same amounts as COI and 16S, except that water was reduced to 14.8 μl, the amount of 100X BSA was increased to 2 μl, and the amount of template DNA reduced to 0.5 μl. The COI and 16S thermoprofile was: 5 minutes at 94 °C; 35 cycles of 40 seconds at 94 °C, 1 minute at 46 °C, and 1 minute at 72 °C; and 10 minutes at 72 °C. The 12S thermoprofile was the same as for COI, except that it was run for 40 cycles. The 28S thermoprofile was: 4 minutes at 94 °C; 38 cycles of 30 seconds at 94 °C, 50 seconds at 52 °C, and 2 and a half minutes at 72 °C; and 10 minutes at 72 °C. The thermoprofile used for ITS2 was: 1 minute at 96 °C; 35 cycles of 30 seconds at 94 °C, 30 seconds at 50 °C, and 1 minute at 72 °C; and a final extension of 10 minutes at 72 °C. The thermoprofile used for ITS1 was: 2 minutes at 95 °C; 36 cycles of 45 seconds at 95 °C, 1 minute at 55 °C, and 1 minute 30 seconds at 72 °C; and a final extension of 10 minutes at 72 °C. The PCR products were cleaned with ExoSAP-IT (Affymetrix, Santa Clara, CA, USA) prior to sequencing. Sequenced fragments represented approximately 680 bp of COI, 530 bp of 16S, 350 bp of 12S, 1000 bp of 28S, 680 bp of ITS1 and 5.8S and 580 bp of ITS2 and 28S. Finally, COI sequences were also translated into amino acid sequences in MEGA using the invertebrate mitochondrial genetic code to check for the presence of stop codons (no stop codon was found).

### Phylogenetic analyses

Chromatograms were consulted to resolve rare ambiguous base calls. Consensus sequences for 28S, and when necessary ITS1 and ITS2, were produced by aligning forward and reverse sequences in Geneious Pro 9.1.8 (Biomatters, Auckland, New Zealand). Alignments were obtained using Clustal W in MEGA 7 (Kumar et al. 2016). DNA sequences were all deposited in GenBank and vouchers deposited in museum collections (Table 1). The ends of each alignment were trimmed and sequences were concatenated. The concatenated mitochondrial alignment included 1,472 nucleotide positions: 624 (COI), 472 (16S), and 376 (12S). The concatenated nuclear alignment included 2,309 nucleotide positions: 792 (ITS1 and 5.8S), 504 (ITS2, with overlapping region of 28S removed) and 1,013 (28S). A separate concatenated nuclear alignment was made just with ITS (ITS1 and ITS2) sequences from a larger number of individuals than the nuclear alignment and included 1312 nucleotide positions: 805 (ITS1 and part of 5.8S) and 507 (ITS2), which are longer than the original sequenced fragments due to the insertion of gaps (largely due to the comparison with outgroups).

**Table 1.** GenBank accession numbers for DNA sequences. All sequences are new, except for the two specimens from China (Sun et al. 2014) and for some of the outgroups (Dayrat et al. 2011, 2016, 2017; Dayrat and Goulding 2017).

Species	Individual (DNA)	Locality	GenBank COI	GenBank 16S	GenBank 12S	GenBank ITS1	GenBank ITS2	GenBank 28S
***W. sinanui***	2737 H	Ambon, Indonesia	MG970711	MG970879	MG970945			
	2738	Ambon, Indonesia	MG970712	MG970880	MG970946			
	2740	Ambon, Indonesia	MG970713	MG970881	MG970947	MG971020	MG971093	MG971161
	2746	Ambon, Indonesia	MG970714					
	5844	Ambon, Indonesia	MG970715			MG971021	MG971094	MG971162
	5845	Ambon, Indonesia	MG970716			MG971022	MG971095	MG971163
	5846	Ambon, Indonesia	MG970717					
***W. uncinus***	2250	Sulawesi, Indonesia	MG970718	MG970882	MG970948			
	2256	Sulawesi, Indonesia	MG970719	MG970883	MG970949	MG971023	MG971096	MG971164
	2261	Sulawesi, Indonesia	MG970720					
	2268	Sulawesi, Indonesia	MG970721			MG971024	MG971097	MG971165
	2751 H	Ambon, Indonesia	MG970722	MG970884	MG970950			
	2752	Ambon, Indonesia	MG970723					
	2843	Ambon, Indonesia	MG970724					
	3138	Bali, Indonesia	MG970725	MG970885	MG970951			
	5029	Halmahera, Indonesia	MG970726			MG971025	MG971098	MG971166
	5056	Halmahera, Indonesia	MG970727					
	5070	Halmahera, Indonesia	MG970728					
	5079	Halmahera, Indonesia	MG970729	MG970886	MG970952			
	5080	Halmahera, Indonesia	MG970730					
	5900	Timor, Indonesia	MG970731	MG970887	MG970953	MG971026	MG971099	MG971167
***W. buetschlii***	2120	Sulawesi, Indonesia	MG970732			MG971027	MG971100	
	2122	Sulawesi, Indonesia	MG970733					
	2189	Sulawesi, Indonesia	MG970734	MG970888	MG970954	MG971028	MG971101	MG971168
	2249	Sulawesi, Indonesia	MG970735			MG971029	MG971102	
	2310	Sulawesi, Indonesia	MG970736					
	2554	Queensland, Australia	MG970737	MG970889	MG970955	MG971030	MG971103	MG971169
	2555	Queensland, Australia	MG970738					
	2722	Ambon, Indonesia	MG970739	MG970890	MG970956	MG971031	MG971104	MG971170
	2853	Ambon, Indonesia	MG970740					
	2872	Seram, Indonesia	MG970741					
	2928	Kei, Indonesia	MG970742					
	2933	Kei, Indonesia	MG970743					
	2982	Lombok, Indonesia	MG970744			MG971032	MG971105	
	2989	Lombok, Indonesia	MG970745	MG970891	MG970957			
	3123	Bali, Indonesia	MG970746					
	3128	Bali, Indonesia	MG970747			MG971033	MG971106	MG971171
	3159	Luzon, Philippines	MG970748					
	3409	Bohol, Philippines	MG970749	MG970892	MG970958	MG971034	MG971107	MG971172
	3410	Bohol, Philippines	MG970750					
	3631	Bohol, Philippines	MG970751					
	3637	Bohol, Philippines	MG970752					
***W. buetschlii***	5016	Halmahera, Indonesia	MG970753	MG970893	MG970959			
	5067	Halmahera, Indonesia	MG970754					
	5898	Timor, Indonesia	MG970755					
***W. gracile***	2252	Sulawesi, Indonesia	MG970756					
	2276	Sulawesi, Indonesia	MG970757					
	2277	Sulawesi, Indonesia	MG970758	MG970894	MG970960	MG971035	MG971108	MG971173
	2280	Sulawesi, Indonesia	MG970759					
	3106	Bali, Indonesia	MG970760					
	3107	Bali, Indonesia	MG970761	MG970895	MG970961			
	3633	Bohol, Philippines	MG970762					
	3648	Bohol, Philippines	MG970763	MG970896	MG970962	MG971036	MG971109	MG971174
	3652	Bohol, Philippines	MG970764					
	3653	Bohol, Philippines	MG970765	MG970897	MG970963	MG971037	MG971110	MG971175
	5158	Halmahera, Indonesia	MG970766	MG970898	MG970964			
	5159	Halmahera, Indonesia	MG970767					
	5932	Timor, Indonesia	MG970768	MG970899	MG970965			
***W. nangkauriense***	1074	Andaman Islands, India	MG970769	MG970900	MG970966			
	1075	Andaman Islands, India	MG970770					
	1786	Sumatra, Indonesia	MG970771	MG970901	MG970967			
	1787	Sumatra, Indonesia	MG970772			MG971038	MG971111	MG971176
	1788	Sumatra, Indonesia	MG970773			MG971039	MG971112	
	2156	Sulawesi, Indonesia	MG970774					
	2161	Sulawesi, Indonesia	MG970775	MG970902	MG970968	MG971040	MG971113	MG971177
	2192	Sulawesi, Indonesia	MG970776	MG970903	MG970969			
	2257	Sulawesi, Indonesia	MG970777					
	2731	Ambon, Indonesia	MG970778	MG970904	MG970970	MG971041	MG971114	MG971178
	2972	Lombok, Indonesia	MG970779			MG971042	MG971115	
	3129	Bali, Indonesia	MG970780					
	3136	Bali, Indonesia	MG970781	MG970905	MG970971	MG971043	MG971116	MG971179
	3162	Luzon, Philippines	MG970782			MG971044	MG971117	
	3208	Luzon, Philippines	MG970783			MG971045	MG971118	
	3217	Luzon, Philippines	MG970784			MG971046	MG971119	
	3268	Bohol, Philippines	MG970785			MG971047	MG971120	
	3269	Bohol, Philippines	MG970786	MG970906	MG970972	MG971048	MG971121	MG971180
	3276	Bohol, Philippines	MG970787					
	3396	Bohol, Philippines	MG970788					
	3401	Bohol, Philippines	MG970789					
	3427	Bohol, Philippines	MG970790					
	5763	Bohol, Philippines	MG970791			MG971049	MG971122	
***W. ater*** unit #1	2157	Sulawesi, Indonesia	MG970792					
	2164	Sulawesi, Indonesia	MG970793	MG970907	MG970973	MG971050	MG971123	MG971181
	2170	Sulawesi, Indonesia	MG970794					
	2221	Sulawesi, Indonesia	MG970795			MG971051	MG971124	MG971182
	2283	Sulawesi, Indonesia	MG970796					
	2330	Sulawesi, Indonesia	MG970797			MG971052	MG971125	
	2727	Ambon, Indonesia	MG970798			MG971053	MG971126	
	2939	Kei, Indonesia	MG970799	MG970908	MG970974	MG971054	MG971127	MG971183
***W. ater*** unit #1	2966	Lombok, Indonesia	MG970800					
	2970	Lombok, Indonesia	MG970801			MG971055	MG971128	
	2974	Lombok, Indonesia	MG970802					
	2978	Lombok, Indonesia	MG970803					
	3130	Bali, Indonesia	MG970804	MG970909	MG970975	MG971056	MG971129	MG971184
	3210	Luzon, Philippines	MG970805					
	3215	Luzon, Philippines	MG970806					
	3265	Bohol, Philippines	MG970807			MG971057	MG971130	
	3266	Bohol, Philippines	MG970808			MG971058	MG971131	
	3272	Bohol, Philippines	MG970809	MG970910	MG970976	MG971059	MG971132	MG971185
	3373	Bohol, Philippines	MG970810					
	3393	Bohol, Philippines	MG970811			MG971060	MG971133	
	3404	Bohol, Philippines	MG970812					
	3405	Bohol, Philippines	MG970813					
	3406	Bohol, Philippines	MG970814					
	3624	Bohol, Philippines	MG970815					
	3629	Bohol, Philippines	MG970816					
	3634	Bohol, Philippines	MG970817					
	5057	Halmahera, Indonesia	MG970818					
	5078	Halmahera, Indonesia	MG970819					
	5121	Halmahera, Indonesia	MG970820	MG970911	MG970977	MG971061	MG971134	MG971186
***W. ater*** unit #2	2145	Sulawesi, Indonesia	MG970821					
	2177	Sulawesi, Indonesia	MG970822	MG970912	MG970978			
	2220	Sulawesi, Indonesia	MG970823					
	2228	Sulawesi, Indonesia	MG970824	MG970913	MG970979			
	2986	Lombok, Indonesia	MG970825					
	3132	Bali, Indonesia	MG970826			MG971062	MG971135	MG971187
	3137	Bali, Indonesia	MG970827	MG970914	MG970980	MG971063	MG971136	MG971188
	3591	Bali, Indonesia	MG970828					
	3212	Luzon, Philippines	MG970829			MG971064	MG971137	MG971189
	3270	Bohol, Philippines	MG970830	MG970915	MG970981			
	3271	Bohol, Philippines	MG970831					
	3277	Bohol, Philippines	MG970832					
	3370	Bohol, Philippines	MG970833970833					
	3408	Bohol, Philippines	MG970834					
	3420	Bohol, Philippines	MG970835			MG971065	MG971138	MG971190
	5125	Halmahera, Indonesia	MG970836	MG970916	MG970982			
***W. graniferum*** unit #1	3163	Luzon, Philippines	MG970837	MG970917	MG970983	MG971066	MG971139	MG971191
	3635	Bohol, Philippines	MG970838	MG970918	MG970984	MG971067	MG971140	MG971192
	3636	Bohol, Philippines	MG970839	MG970919	MG970985	MG971068	MG971141	MG971193
	5760	Bohol, Philippines	MG970840	MG970920	MG970986	MG971069	MG971142	MG971194
	5761	Bohol, Philippines	MG970841	MG970921	MG970987	MG971070	MG971143	MG971195
	S211	China (21°06'N)	JN543166					
	S221	China (21°06'N)	JN543167					
***W. graniferum*** unit #2	3638	Bohol, Philippines	MG970842	MG970922	MG970988			
	5762	Bohol, Philippines	MG970843	MG970923	MG970989	MG971071	MG971144	MG971196
	5902	Timor, Indonesia	MG970844	MG970924	MG970990			
***W. achleitneri***	2645	Queensland, Australia	MG970845	MG970925	MG970991	MG971072	MG971145	MG971197
	3534 H	Queensland, Australia	MG970846	MG970926	MG970992	MG971073	MG971146	MG971198
	3535	Queensland, Australia	MG970847	MG970927	MG970993	MG971074	MG971147	MG971199
***W. comendadori***	2315	Sulawesi, Indonesia	MG970848	MG970928	MG970994			
	2725	Ambon, Indonesia	MG970849			MG971075	MG971148	MG971200
	3539	Ambon, Indonesia	MG970850	MG970929	MG970995	MG971076	MG971149	MG971201
	2983	Lombok, Indonesia	MG970851					
	2985	Lombok, Indonesia	MG970852	MG970930	MG970996			
	3131	Bali, Indonesia	MG970853					
	3133	Bali, Indonesia	MG970854	MG970931	MG970997			
	3626 H	Bohol, Philippines	MG970855	MG970932	MG970998	MG971077	MG971150	MG971202
	3385	Bohol, Philippines	MG970856					
	3400	Bohol, Philippines	MG970857	MG970933	MG970999	MG971078	MG971151	MG971203
	3627	Bohol, Philippines	MG970858			MG971079	MG971152	
	3630	Bohol, Philippines	MG970859			MG971080	MG971153	
***W. melanesiensis*** unit #1	2202	Sulawesi, Indonesia	MG970860					
	2215	Sulawesi, Indonesia	MG970861	MG970934	MG971000			
	2732	Ambon, Indonesia	MG970862	MG970935	MG971001			
	2733	Ambon, Indonesia	MG970863					
	2735	Ambon, Indonesia	MG970864	MG970936	MG971002			
	5065	Halmahera, Indonesia	MG970865	MG970937	MG971003			
	5026	Halmahera, Indonesia	MG970866	MG970938	MG971004			
	5131	Halmahera, Indonesia	MG970867					
	5132	Halmahera, Indonesia	MG970868					
	5133	Halmahera, Indonesia	MG970869					
	5417 H	Papua New Guinea	MG970870	MG970939	MG971005	MG971081	MG971154	MG971204
	5421	Papua New Guinea	MG970871			MG971082	MG971155	
	5446	Papua New Guinea	MG970872	MG970940	MG971006	MG971083	MG971156	MG971205
	5483	Vanuatu	MG970873	MG970941	MG971007	MG971084	MG971157	MG971206
	5484	Vanuatu	MG970874	MG970942	MG971008	MG971085	MG971158	MG971207
	6089	New Ireland, PNG	MG970875					
	6090	New Ireland, PNG	MG970876			MG971086	MG971159	
***W. melanesiensis*** unit #2	2963	Lombok, Indonesia	MG970877	MG970943	MG971009	MG971087	MG971160	MG971208
*Platevindex luteum*		Singapore	MG958714	MG958716	MG971010	MG971088	MG958718	MG958888
*Peronina tenera*		Peninsular Malaysia	MG958740	MG958796	MG971011			
*Onchidina australis*		New South Wales, Australia	KX179548	KX179561	MG971012	MG971089	MG958719	MG971209
*Onchidium typhae*		Peninsular Malaysia	KX179509	KX179525	MG971013	MG971090	MG958720	MG971210
*Onchidium stuxbergi*		Vietnam	KX179520	KX179537	MG971014	MG971091	MG958721	MG971211
*Peronia* sp.		Okinawa, Japan	HQ660043	HQ659911	MG971015			
*Peronia* sp.		Hawaii, USA	HQ660038	HQ659906	MG971016	MG971092	MG958722	MG971212
*Onchidella floridana*		Tobago	HQ660035	HQ659903	MG971017			
*Onchidella celtica*		France	MG958715	MG958717	MG971018			
*Onchidella patelloide*		New South Wales, Australia	MG970878	MG970944	MG971019			

Four independent sets of phylogenetic analyses were performed: 1) Maximum Likelihood and Bayesian analyses with just COI sequences, performed with 169 individuals (not counting outgroups); 2) Maximum Likelihood and Bayesian analyses with concatenated COI, 16S, and 12S sequences, performed with 65 individuals (not counting outgroups); 3) Maximum Likelihood, Bayesian, and Maximum Parsimony analyses with concatenated ITS1, ITS2 and 28S sequences, performed with 48 individuals (not counting outgroups); and 4) Maximum Parsimony analyses with concatenated ITS1 and ITS2 sequences, performed with 68 individuals (not counting outgroups).

Prior to phylogenetic analyses, the best-fitting evolutionary model for each marker was selected using the Model Selection option from TOPALi v2.5 (Milne et al. 2004). A GTR + G model was selected independently for the mitochondrial markers and 28S, and a HKY + G model for the nuclear markers. Maximum Likelihood analyses were performed using PhyML (Guindon and Gascuel 2003) as implemented in TOPALi v2.5. Other (unpublished) analyses were performed using different models, which all yielded identical results. Node support was evaluated using bootstrapping with 100 replicates. Bayesian analyses for mitochondrial loci were performed using MrBayes v3.1.2 (Ronquist and Huelsenbeck 2003) as implemented in TOPALi v2.5, with five simultaneous runs of 1.5 × 10^6^ generations each, sample frequency of 100, and burn-in of 25% (and posterior probabilities were also calculated). Maximum Parsimony analyses were conducted in PAUP v4.0 (Swofford 2002), with gaps coded as a 5^th^ character state (because insertions and deletions are the most informative characters for ITS1 and ITS2) and 100 bootstrap replicates conducted using a full heuristic search. All analyses were run several times and yielded the same result. Ten other onchidiid species and their corresponding sequences were selected from previous studies from our lab as out-groups (Dayrat et al. 2011, 2016, 2017; Dayrat and Goulding 2017): *Onchidella floridana* (Dall, 1885), *Onchidella celtica* (Cuvier *in* Audouin and Milne-Edwards, 1832), *Onchidella patelloide* (Quoy & Gaimard, 1832), *Onchidina australis* (Semper, 1880), *Platevindex luteum* (Semper, 1880), *Peronina tenera* (Stoliczka, 1869), *Peronia* sp. (Okinawa), *Peronia* sp. (Hawaii), *Onchidium stuxbergi* (Westerlund, 1883), and *Onchidium typhae* Buchannan, 1800. Finally, pairwise genetic distances between COI sequences were calculated in MEGA 7 (Kumar et al. 2016).

## Phylogenetic results

### Molecular phylogenetic analyses (Figs 1–5)

DNA sequences were used to test species limits, species phylogenetic relationships and the monophyly of *Wallaconchis*. More specifically, mitochondrial and nuclear sequences were used independently to determine whether different markers may yield different results.

The monophyly of *Wallaconchis* is strongly supported in all analyses (Figs 1–5). Two monophyletic groups within *Wallaconchis* are also strongly supported in all analyses: clade A includes two species and clade B includes eight species (Figs 1–5).

**Figure 1.**
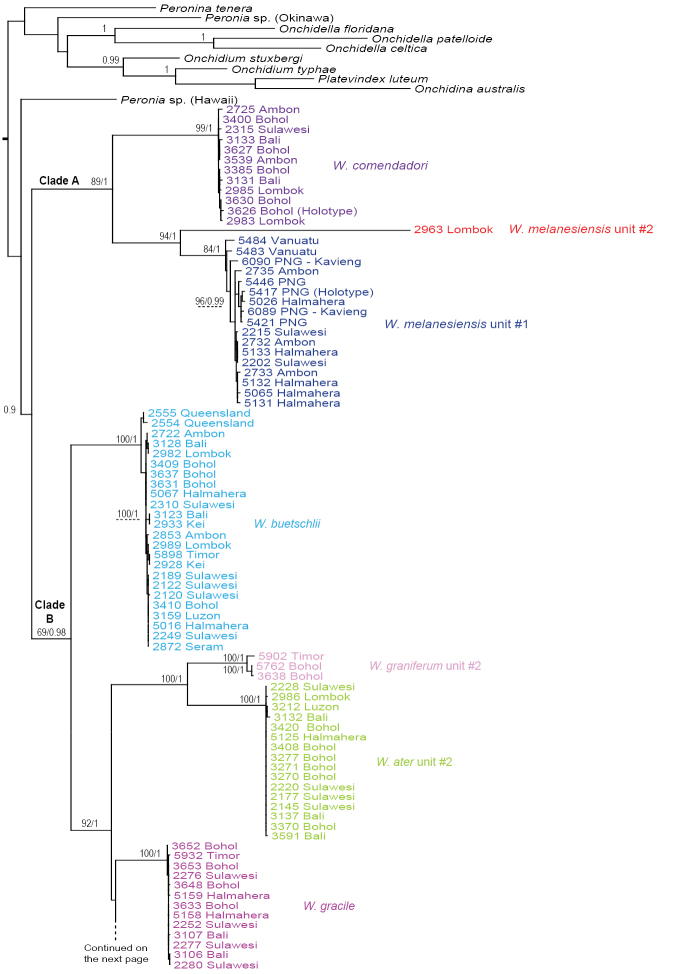
Phylogenetic tree based on mitochondrial COI sequences. Numbers by branches show bootstrap values (Maximum Likelihood analysis) and posterior probabilities (Bayesian analysis). Only significant numbers (>80% and >0.9) are indicated. *Onchidella*, *Peronia*, *Peronina*, *Platevindex*, *Onchidium*, *Onchidina*, and *Melayonchis* sequences serve as outgroups. Numbers for each individual correspond to unique identifiers used for DNA extraction. All sequences of *Wallaconchis* specimens are new, except two sequences obtained from GenBank (from specimens from China). Information on individually-identified specimens can be found in the lists of material examined and in Table 1. The color used for each species is the same as the color used in the other trees (Figs 2–5) and on the map of species distribution (Fig. 6).

**Figure 1. Continued.**
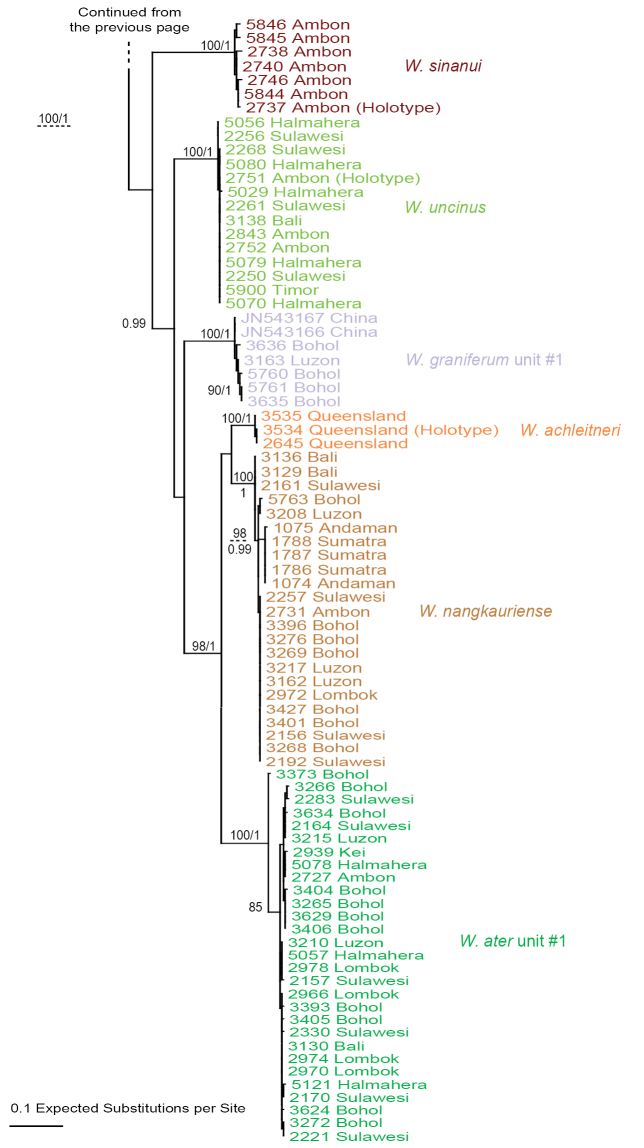


**Figure 2.**
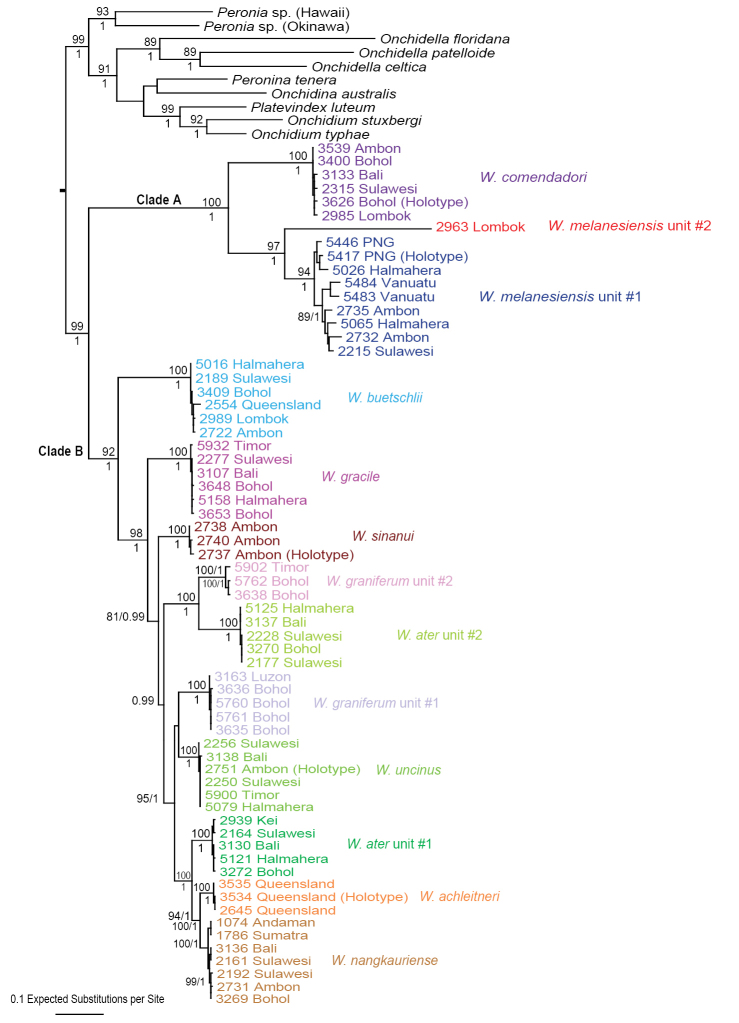
Phylogenetic tree based on concatenated mitochondrial COI, 16S, and 12S sequences. Numbers by branches show bootstrap values (Maximum Likelihood analysis) and posterior probabilities (Bayesian analysis). Only significant numbers (>80% and >0.9) are indicated. All sequences of *Wallaconchis* specimens are new. Information on individually-identified specimens can be found in the lists of material examined and in Table 1. The color used for each species is the same as the color used in the other trees (Figs 1, 3–5) and on the map of species distribution (Fig. 6).

Analyses of COI sequences yielded 13 least-inclusive molecular units, all reciprocally monophyletic and highly supported, with bootstrap values above 99 and posterior probabilities of 1 (Fig. 1). The support for *W. melanesiensis* unit #1 is slightly lower (bootstrap value of 84, and posterior probability of 1) due to divergent and basal sequences from Vanuatu. Three of those 13 units are within clade A and ten of them within clade B. The multilocus mitochondrial analyses (COI, 16S, and 12S) yielded the same 13 units as the COI analyses, all being highly supported (Fig. 2).

**Figure 3.**
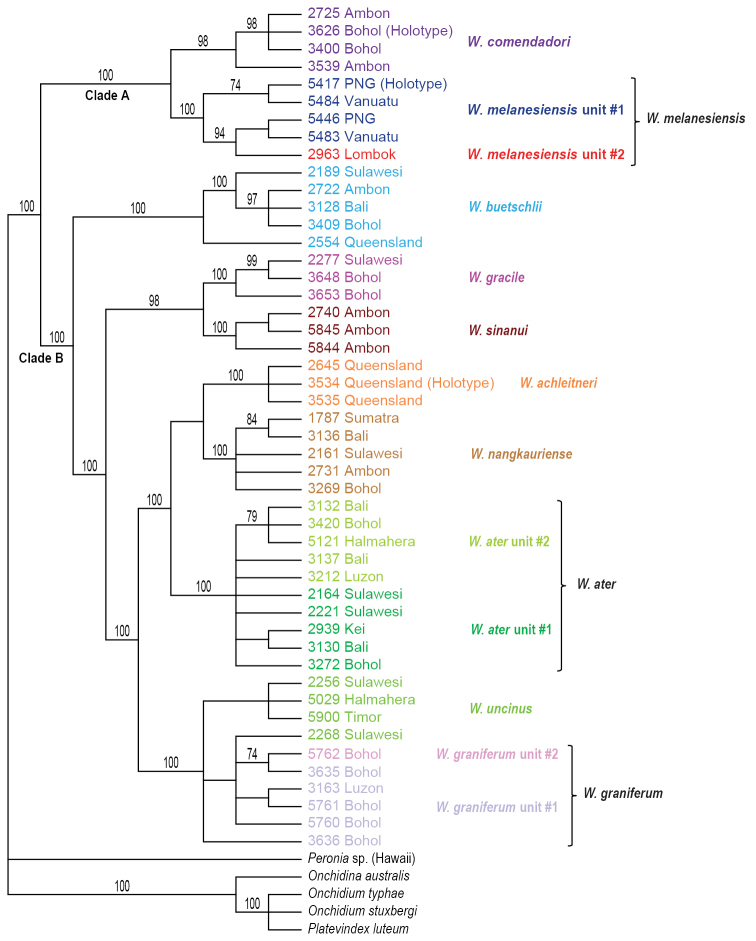
Maximum parsimony bootstrap consensus tree based on concatenated nuclear ITS1, ITS2, and 28S sequences. Only bootstrap values >70% are indicated. All sequences of *Wallaconchis* specimens are new. Information on individually-identified specimens can be found in the lists of material examined and in Table 1. The color used for each species is the same as the color used in the other trees (Figs 1–2, 4–5) and on the map of species distribution (Fig. 6).

**Figure 4.**
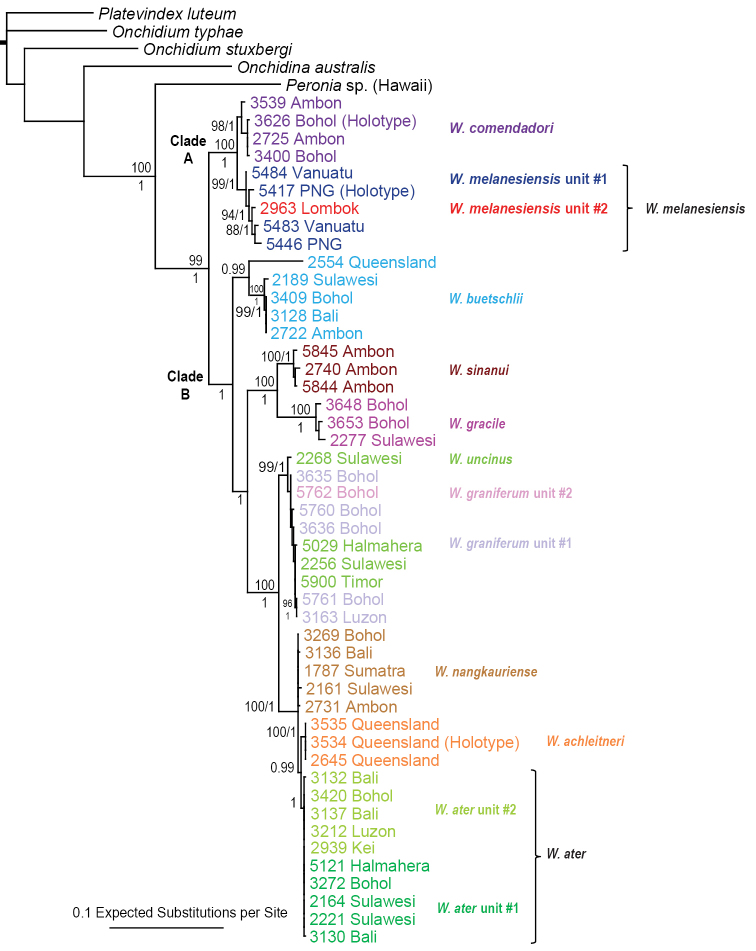
Maximum Likelihood phylogenetic tree based on concatenated nuclear ITS1, ITS2, and 28S sequences. Numbers by branches show bootstrap values (only numbers >80% are indicated). All sequences of *Wallaconchis* specimens are new. Information on individually-identified specimens can be found in the lists of material examined and in Table 1. The color used for each species is the same as the color used in the other trees (Figs 1–3, 5) and on the map of species distribution (Fig. 6).

**Figure 5.**
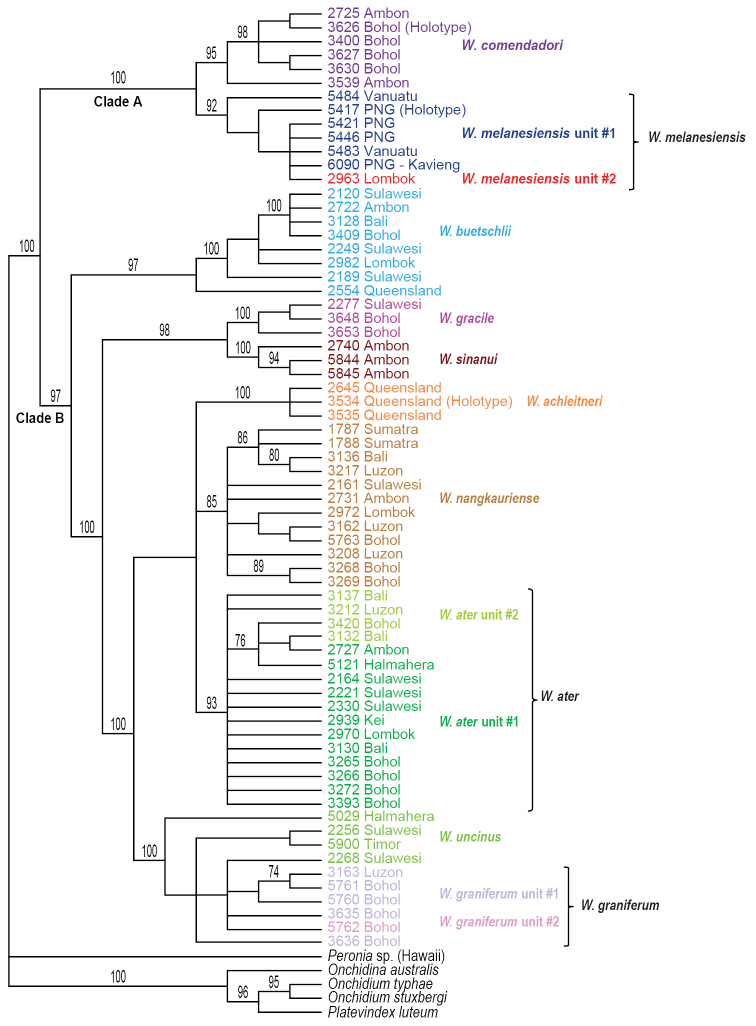
Maximum parsimony bootstrap consensus tree based on concatenated nuclear ITS1 and ITS2 sequences. Only bootstrap values >70% are indicated. All sequences of *Wallaconchis* specimens are new. Information on individually-identified specimens can be found in the lists of material examined and in Table 1. The color used for each species is the same as the color used in the other trees (Figs 1–4) and on the map of species distribution (Fig. 6).

Analyses based on nuclear sequences, whether with all three markers (ITS1, ITS2, 28S) (Figs 3–4) or only with ITS 1 and ITS 2 (Fig. 5), yielded fewer molecular units than the mitochondrial sequences. Indeed, four of the 13 mitochondrial units (*W. ater* unit #2, *W. graniferum* unit #2, *W. melanesiensis* unit #2, and *W. uncinus*) are not recovered as distinct, reciprocally-monophyletic, least-inclusive units with nuclear loci. For instance, individuals from *W. ater* unit #2 are mixed in the same nuclear unit as individuals from *W. ater* unit #1. In the case of *W. melanesiensis* units #1 and #2, there is no phylogenetic incongruence between mitochondrial and nuclear results: *Wallaconchis melanesiensis* unit #2 is mixed with *W. melanesiensis* unit #1 based on nuclear sequences (Figs 3–5) and both units are sister-group according to mitochondrial results (Fig. 2). In the case of *W. uncinus*, there is no incongruence between mitochondrial and nuclear results: it is closely-related to *W. graniferum* unit #1 based on mitochondrial sequences (Fig. 2) while specimens of both units are mixed together according to nuclear results (Figs 3–5). However, there is some phylogenetic incongruence between the nuclear and mitochondrial phylogenetic relationships regarding *W. ater* unit #2 and *W. graniferum* unit #2: based on mitochondrial sequences, *W. ater* unit #1 is closely related (with high support) to *W. achleitneri* and *W. nangkauriense*, and *W. graniferum* unit #2 seems closely related (with low support) to *W. ater* unit #2 (Figs 1–2); while, based on nuclear sequences, individuals from *W. graniferum* unit #2 are mixed with individuals from *W. graniferum* unit #1 and *W. uncinus*, and individuals from *W. ater* unit #2 are mixed with individuals from *W. ater* unit #1 (Figs 3–5). This incongruence, which does not affect species delineation (see below), is addressed in detail in the general discussion.

In the Maximum Likelihood analyses based on the three (ITS1, ITS2, and 28S) concatenated nuclear markers (Fig. 4), the monophyly of *W. nangkauriense* is not supported. It likely is due to the fact that insertions and gaps are very informative characters for ITS sequences which are not considered by non-parsimony methods. However, it is important to note that individuals of *W. nangkauriense* form a group of unresolved branches basal to *W. achleitneri* and *W. ater* (Fig. 4), which is fully compatible with a monophyly of *W. nangkauriense* in other trees (Figs 1–3, 5).

**Pairwise genetic divergences** (Table 2). Pairwise genetic distances between COI sequences strongly suggest 13 molecular units of *Wallaconchis*. Genetic divergences between units are always minimally 10.6%, except between *W. nangkauriense* and *W. achleitneri* (between which divergences vary from 6.1 to 7.6%). Also, divergences within units are always very low (below 3.2%), except within *W. melanesiensis* unit #1 in which they reach 5.5%. There is a wide barcode gap between intra- and inter-unit distances. The barcode gap between closely-related units is minimally 4.4% (because the divergences within *W. nangkauriense* are up to 1.7% and the divergences between *W. nangkauriense* and *W. achleitneri* are minimally 6.1%). However, in most cases, the gap between intra- and inter-unit divergences in *Wallaconchis* is more than 10%.

**Table 2.** Pairwise genetic distances of COI sequences within and between mitochondrial molecular units. Ranges of minimum to maximum distances are indicated (in percentage). For instance, within *W. ater* unit #1, individual sequences are between 0% and 2.8% divergent, but sequences of *W. ater* unit # 1 and *W. ater* unit #2 are between 25.7% and 28.9% divergent.

Species	1	2	3	4	5	6	7	8	9	10	11	12	13
**1.** *W. uncinus*	0.0–0.4												
**2.** *W. buetschlii*	22.1–22.6	0.0–3.2											
**3.** *W. gracile*	14.9–16.4	19.8–22.0	0.0–1.1										
**4.** *W. nangkauriense*	13.1–15.3	21.2–23.6	14.2–16.3	0.0–2.1									
**5.** *W. ater* unit #1	14.6–17.1	23.1–26.8	16.0–19.1	10.4–13.1	0.0–2.8								
**6.** *W. ater* unit #2	21.4–23.1	25.4–28.2	22.5–24.6	23.4–24.9	25.7–28.9	0.2–1.3							
**7.** *W. graniferum* unit #1	11.0–12.4	20.6–24.4	15.0–16.4	12.8–15.2	14.3–17.0	24.1–25.3	0.0–1.1						
**8.** *W. graniferum* unit #2	19.8–21.4	23.5–26.6	19.8–21.4	20.2–22.3	20.9–24.5	17.9–19.5	21.8–22.6	0.2–2.8					
**9.** *W. sinanui*	14.5–16.5	20.2–23.6	15.4–16.9	14.7–17.0	19.1–21.8	23.3–25.4	15.2–17.2	21.3–23.9	0.2–1.7				
**10.** *W. achleitneri*	14.4–15.3	21.9–24.0	14.5–15.6	6.1–7.5	10.4–12.6	26.4–27.1	13.4–14.6	20.6–21.2	15.0–16.0	0.0–0.2			
**11.** *W. comendadori*	26.8–29.2	23.5–26.6	24.2–26.5	26.1–29.0	29.6–32.3	32.7–34.9	28.2–29.7	31.9–36.1	26.5–29.6	27.6–29.3	0.2–1.9		
**12.** *W. melanesiensis* unit #1	26.0–28.6	27.8–32.9	27.1–31.8	24.3–28.3	25.2–28.4	33.6–37.9	27.9–31.2	32.8–36.9	27.4–30.5	27.5–29.6	23.5–27.4	0.2–5.6	
**13.** *W. melanesiensis* unit #2	32.9–33.9	34.8–36.9	35.2–36.8	36.5–38.1	38.7–40.3	39.5–40.3	36.7–37.2	40.3–41.6	31.3–33.6	37.3–37.7	30.3–33.0	26.3–27.8	–

The divergences between *W. ater* unit #1 and *W. ater* unit #2, between *W. graniferum* unit #1 and *W. graniferum* unit #2, and between *W. melanesiensis* unit #1 and *W. melanesiensis* unit #2 are all extremely high: minimally 21.8% (between the two *W. graniferum* units) to 27.8% (between the two *W. melanesiensis* units). However, nuclear DNA sequences and morphology show that these three pairs of units with such widely divergent haplotypes actually correspond to only three species instead of six (see below). The mitochondrial divergences likely are related to the mode of inheritance and evolution of the mitochondrial genome combined with recent population isolation due to sea level changes (see the Discussion).

**Species delineation.** Determining the species diversity of *Wallaconchis* requires integrating mitochondrial and nuclear data with morphology. Overall, ten *Wallaconchis* species are recognized here: two in clade A and eight in clade B (Figs 1–5). For the most part, nuclear DNA sequences and morphology are congruent. Based on the anatomy of the reproductive system, there are ten species in *Wallaconchis*, each of which are characterized by very distinctive male copulatory organs. Eight of these morphology-based units correspond perfectly to eight of the nuclear molecular units (*W. achleitneri*, *W. ater*, *W. buetschlii*, *W. comendadori*, *W. gracile*, *W. melanesiensis*, *W. nangkauriense*, and *W. sinanui*), and two of them (*W. uncinus* and *W. graniferum*) are mixed together (mostly with unresolved branching) into the ninth nuclear unit (Figs 3–5). Nuclear sequences are likely not variable enough to separate *W. uncinus* and *W. graniferum* of which the copulatory organs are completely distinct. Note that *W. uncinus* and *W. graniferum* are distinct according to mitochondrial sequences too. The very divergent mitochondrial pairs of haplotypes (*W. ater* units #1 and #2, *W. graniferum* units #1 and #2, and *W. melanesiensis* units #1 and #2) are not regarded as pairs of cryptic species because individuals within each species are anatomically indistinguishable: e.g., individuals of *W. ater* unit #1 cannot be distinguished at all from the individuals of *W. ater* unit #2 (see the Discussion).

## Systematics and anatomical descriptions

### Family Onchidiidae Rafinesque, 1815

#### Genus Wallaconchis

Taxon classificationAnimaliaSystellommatophoraOnchidiidae

Goulding & Dayrat gen. n.

http://zoobank.org/35C66C82-140F-4CF8-85A0-C32A4C5F55F8

##### Type species.

*Wallaconchis sinanui*, designated here.

##### Etymology.

Combination of Wallace, for Alfred Russel Wallace, one of the first naturalists to widely study the fauna of the Indo-Malay Archipelago, and *Onchis*, one of the names used to refer to onchidiid slugs. The core of the geographic distribution of this genus in Indonesia (Bali, Lombok, Sulawesi, and eastern Indonesia) corresponds to a region sometimes referred to as Wallacea, also after A. R. Wallace, which makes it especially fitting here.

##### Gender.

Masculine, gender of *Onchis*.

##### Diagnosis.

Body not flattened. Dorsal gills absent. Dorsal eyes present on notum, mostly in groups of three or four eyes. Central dorsal papilla bearing eyes present and retractable, but usually not raised above the dorsal surface. Eyes at the tip of short ocular tentacles. Male opening below the right ocular tentacle. Oral lobes typically grey or dark brown. Pneumostome median. Foot wide. Visceral cavity pigmented (in black), which varies in intensity between individuals. Intestinal loops of type I. Rectal gland absent. Accessory penial gland absent. Penial morphology highly diverse, from short and straight to long and coiled. Penial hooks absent or present.

##### Remarks.

Every available genus name was carefully evaluated when searching for a name for this genus, which included examination of the type specimens of the type species of all existing genera, as well as the analysis of all the original descriptions. Nine existing species names are transferred to *Wallaconchis*, five of which are valid, one of which is a synonym, and three of which are *nomina dubia*. Historically, these five valid species names have been placed in three different genera: *Onchidium* Buchannan, 1800, *Paraoncidium* Labbé, 1934, and *Scaphis* Labbé, 1934. However, all five species were originally described in *Onchidium* (sometimes with the unjustified emendation *Oncidium*), traditionally used by default for many unrelated onchidiids (Dayrat 2009): *Onchidium ater* Lesson, 1830; *Onchidium graniferum* Semper, 1880; *Oncidium nangkauriense* Plate, 1893; *Oncidium buetschlii* Stantschinsky, 1907; and *Oncidium gracile* Stantschinsky, 1907. The one species name which is a synonym, *Oncidium keiense* Hoffmann, 1926, and the three species names which are *nomina dubia* (*Onchidium ovale* Semper, 1880; *Oncidium simrothi* Plate, 1893; *Oncidium fungiforme* Stantschinsky, 1907), were also originally created in the genus *Onchidium*. From this point forward, the unjustified emendation *Oncidium* is not repeated throughout the paper and is systematically replaced by the correct spelling *Onchidium*. The genus *Onchidium* actually refers to a different taxon including only three species and characterized by features (e.g., large dorsal conical papillae, rectal gland, accessory penial gland) that are absent in the species described here (see Dayrat et al. 2016). Hoffmann (1926) decided not to create a new genus for *Onchidium keiense*, despite the distinctive morphology of the copulatory parts: “The peculiarity of the penis could almost suggest the idea that we are dealing here with a whole new genus. In the genus *Onchidium*, however, there are already species which differ quite considerably from each other, so that I have no reason to create a new genus for my species.” (1926: 24, translated from German).

Hoffmann (1928) briefly mentioned the morphological similarity of six *Onchidium* species (*Onchidium papuanum* Semper, 1880, *Onchidium palaense* Semper, 1880, *Onchidium ovale*, *O. buetschlii*, *O. nangkauriense*, and *Onchidium chameleon* Brazier, 1886) which all lack an accessory penial gland, and five of which also lack a rectal gland: “The following six species are quite close to one another, and there are usually very few characteristic features which can be used for differentiation.” (1928: 81) Hoffmann (1928) also mentioned that some of those six names would likely become synonyms as new material became available, but none of these names has been proposed as synonymous. Three of these names are transferred to *Wallaconchis* here (*O. ovale*, *O. buetschlii*, and *O. nangkauriense*). *Onchidium chameleon* belongs to the genus *Onchidina* Semper, 1882 (Dayrat and Goulding 2017). *Onchidium palaense* and *O. papuanum* are poorly known because their type material is lost. However, Semper’s description of the position of the male aperture as “between the two very small eye tentacles” in *O. palaense* (1882: 276) and “exactly in the center line between the tentacles and mouth” in *O. papuanum* (1882: 277) indicates that they belong to another genus. A few years later, Labbé (1934) created the genus name *Paraoncidium* for eleven *Onchidium* species with no accessory penial gland, including the six aforementioned species considered morphologically similar by Hoffmann (1928). However, *P. chameleon*, the type species of *Paraoncidium*, belongs to the genus *Onchidina* and *Paraoncidium* is a junior synonym of *Onchidina* (Dayrat and Goulding 2017). The genus *Onchidina*, which so far is monotypic, differs greatly from *Wallaconchis* (e.g., dorsal eyes absent, male aperture on right lateral side of right ocular tentacle, rectal gland present).

Finally, a new combination was proposed by Labbé (1934) for *Onchidium ater* as *Scaphis atra*. However, *Scaphis* Starobogatov, 1976 is a synonym of *Peronia* Fleming, 1822 which refers to a different group of onchidiids characterized by dorsal gills, which are lacking in all the species described here.

Overall, none of the three genus names historically used in binomials for the species described here (*Onchidium*, *Paraoncidium*, *Scaphis*) could apply to our new genus.

##### Geographic distribution

**Figure 6.**
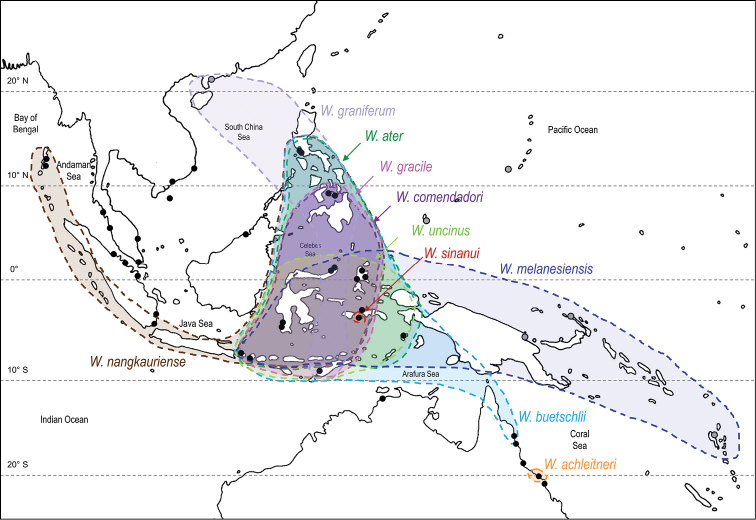
Geographic distribution of *Wallaconchis* species. The colors used for each species are the same as those used in phylogenetic trees (Figs 1–5), but note that for *W. ater*, *W. graniferum*, and *W. melanesiensis*, both units #1 and #2 are included within the distribution of the species and that only the color of unit #1 is being used. Black dots show sites sampled by the authors, and grey dots (in Papua New Guinea and the Pacific) show localities where samples were borrowed from a museum collection.

(Fig. 6). *Wallaconchis* species are distributed from the Andaman Islands (India) in the Bay of Bengal to Vanuatu in the western Pacific. Nine out of ten species are sympatric over at least part of their distribution. Eight out of ten species are found widely within the Coral Triangle. Two species are known from only one station each, which suggests that they are endemic to a small region and specialized to a particular habitat. All species are found in tropical waters.

##### Habitat

(Table 3). *Wallaconchis* slugs are found in various intertidal habitats. Two new species (*W. comendadori* and *W. melanesiensis*) live predominantly in the rocky intertidal, with large rocks covered in thin layers of algae, which may or may not be adjacent to mangrove trees. Two species (*W. nangkauriense* and *W. ater*) are commonly found in coral rubble habitats, where *Avicennia* trees often grow. Three species (*W. buetschlii*, *W. uncinus*, and *W. sinanui*) are found on firm mud (i.e., mud which is not deep and not saturated in water). Two species (*W. gracile* and *W. buetschlii*) are found on sandy mud, two species (*W. achleitneri* and *W. buetschlii*) on coarse sand, and one species (*W. graniferum*) on fine sand. Many *Wallaconchis* species can be found together on diverse substrates at stations with various habitats. For instance, at our station 198, on the southern coast of Bohol, Philippines, six species were present without any other onchidiids on just a few square meters of fine sand mixed with coral rubble and muddy sand.

**Table 3.** Habitats where *Wallaconchis* species occur.

Species	Mud	Coarse sand	Coral rubble	Rocks	Sandy mud	Fine sand
*W. sinanui*	✓					
*W. uncinus*	✓					
*W. buetschlii*	✓	✓			✓	
*W. gracile*					✓	
*W. nangkauriense*			✓			
*W. ater*			✓			
*W. graniferum*						✓
*W. achleitneri*		✓				
*W. comendadori*				✓		
*W. melanesiensis*				✓		

##### Distinctive diagnostic features.

A unique combination of characters can be used to determine whether a specimen is part of a *Wallaconchis* species: no dorsal gills, intestinal loops of type I, no rectal gland, and no accessory penial gland. This combination is not found in any other genus and these anatomical features can be easily and quickly observed through dissection. It is more challenging to identify *Wallaconchis* slugs using external characters, but it is not impossible. *Wallaconchis* slugs exhibit the highest degree of color variation in the Onchidiidae. In some species, the dorsal notum can be red, yellow, green, orange, brown, grey, black, etc., when most onchidiids are usually just brown, marbled with light to dark brown. Such bright dorsal colors are also found in *Peronia*, but *Peronia* is easily distinguished from *Wallaconchis* (the dorsal notum of *Peronia* bears gills that are absent in *Wallaconchis*). However, the three *Wallaconchis* species which are found on mud inside and around mangroves only occasionally exhibit patches of bright colors, and thus, can be easily mistaken for other genera found in mangroves. The male opening can also help identify *Wallaconchis* slugs at the generic level. In other genera, the male opening may be to the left of the right ocular tentacle (e.g., *Melayonchis*, *Onchidium*), or to the right of the right tentacle (e.g., *Peronina*, *Onchidina*), while in *Wallaconchis* it is directly below the right ocular tentacle (Dayrat et al. 2016, 2017, Goulding et al. in press). Another character which may be observed in *Wallaconchis* by naturalists in the field, although difficult to appreciate, is that live animals produce a sticky mucus from the ventral surface, which is most noticeable with large animals. Other onchidiids also produce some mucus, but that mucus is usually wet, while the mucus of *Wallaconchis* can be likened to drying glue.

#### Wallaconchis sinanui

Taxon classificationAnimaliaSystellommatophoraOnchidiidae

Goulding & Dayrat sp. n.

http://zoobank.org/DF209468-F30C-41CD-982A-1A0A23485AA1

**Figure 7.**
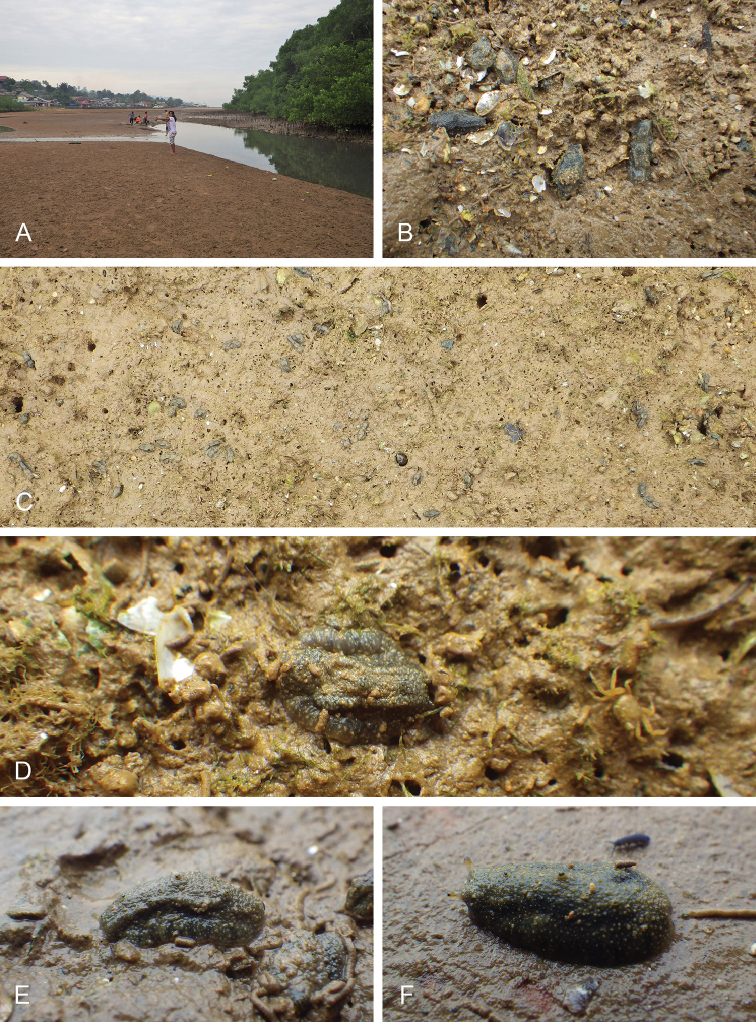
Habitats and live specimens, *Wallaconchis sinanui*, Indonesia, Ambon, Lateri. **A** Type locality, mudflat adjacent to a shallow river through a mangrove (st 128) **B** Close up of mud with a few individuals of *W. sinanui* (same locality as A) **C** Wider view of the mud with many individuals of *W. sinanui* (same locality as A) **D** Dorsal view, 9 mm long [2746] (UMIZ 00059) **E** Holotype, dorsal view, 9 mm long [2737] (UMIZ 00058) **F** Dorsal view, 9 mm long [2740] (UMIZ 00059).

**Figure 8.**
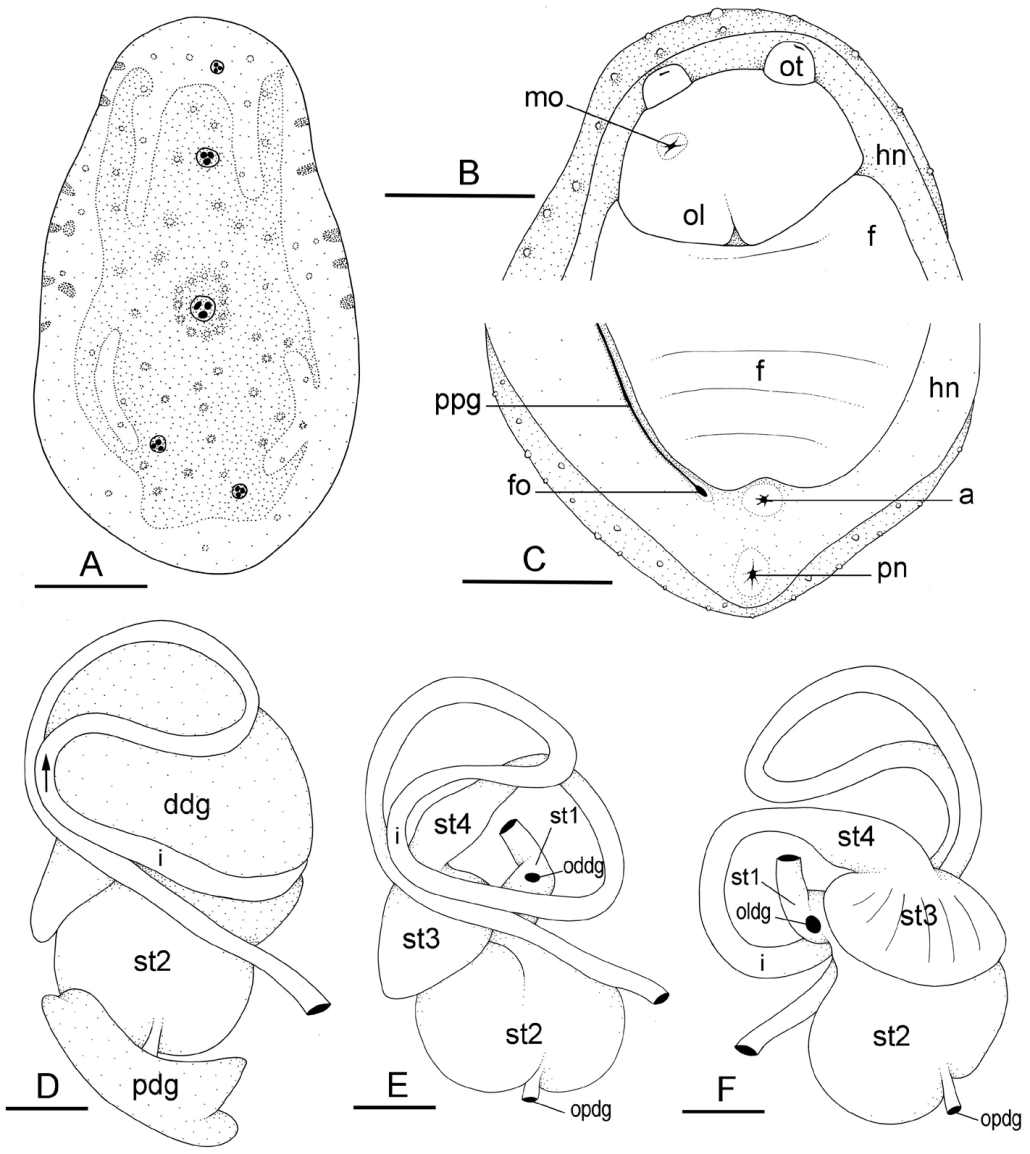
External morphology and digestive system, *Wallaconchis sinanui*, Indonesia, Ambon (**A–C**) holotype [2737] (UMIZ 00058) (**D–F**) [2738] (UMIZ 00059). **A** Dorsal notum **B** Anterior region, ventral view **C** Posterior region, ventral view **D** Digestive system, dorsal view **E** Digestive system, dorsal view (digestive gland removed) **F** Digestive system, ventral view (digestive gland removed). Abbreviations: **a** anus **ddg** dorsal digestive gland **f** foot **fo** female opening **hn** hyponotum **i** intestine **mo** male opening **oddg** opening of the dorsal lobe of the digestive gland **oldg** opening of the lateral lobe of the digestive gland **opdg** opening of the posterior lobe of the digestive gland **ol** oral lobe **ot** ocular tentacle **pn** pneumostome **pdg** posterior lobe of the digestive gland **ppg** peripodial groove **st1** first stomach chamber **st2** second stomach chamber **st3** third stomach chamber **st4** fourth stomach chamber. Scale bars: 2 mm (**A, B, C**); 1 mm (**D, E, F**).

**Figure 9.**
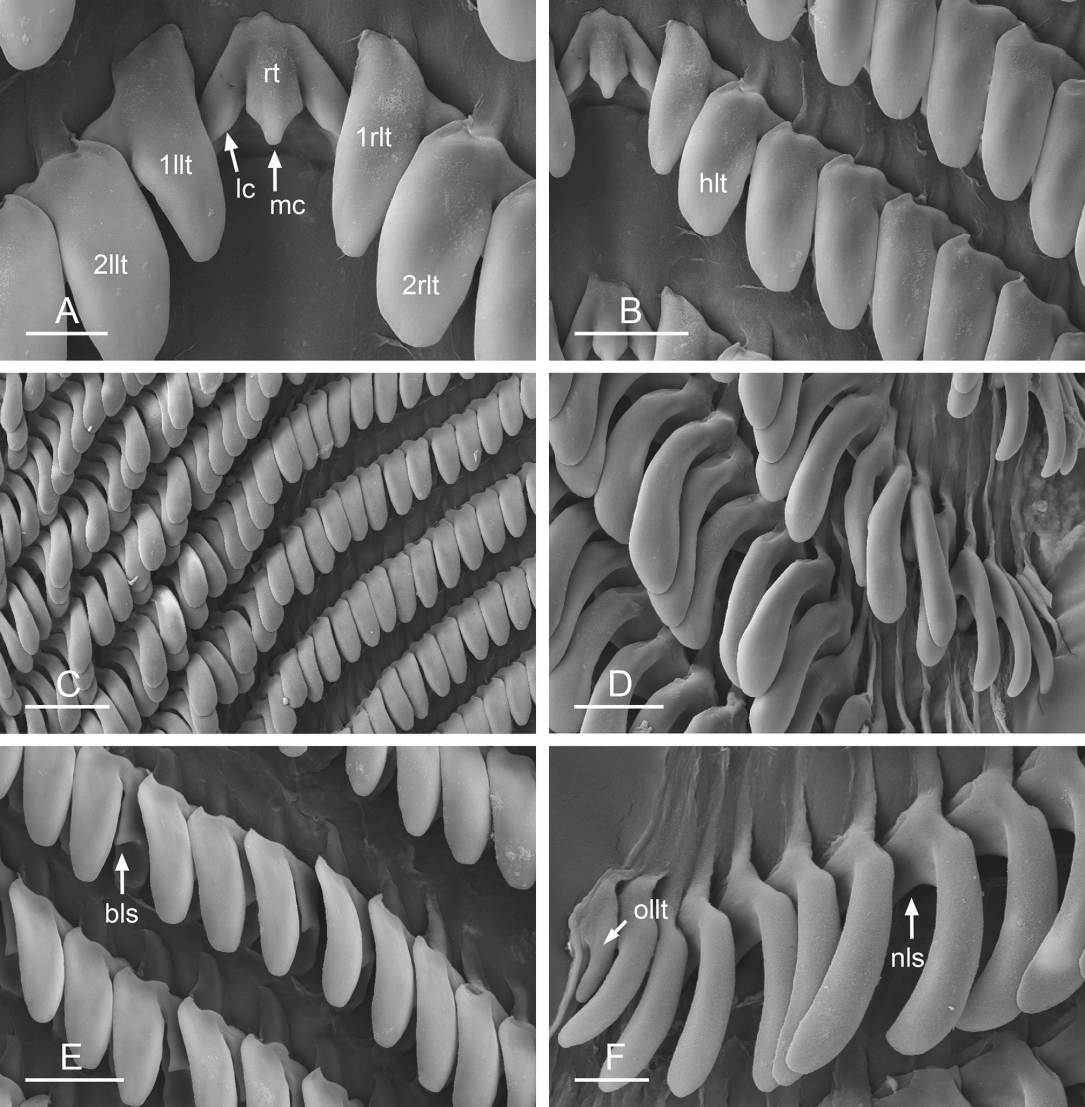
Radula, *Wallaconchis sinanui*, Indonesia, Ambon [5845] (UMIZ 00059). **A** Rachidian and innermost lateral teeth **B** Rachidian and innermost lateral teeth**C** Transition between inner lateral teeth and outer lateral teeth **D** Outermost lateral teeth **E** Right lateral teeth with basal lateral spine **F** Outermost lateral teeth. Abbreviations: **rt** rachidian tooth **mc** median cusp **lc** lateral cusp **1llt** first left lateral tooth **1rlt** first right lateral tooth **2llt** second left lateral tooth **2rlt** second right lateral tooth **bls** basal lateral spine (of lateral tooth) **nls** no lateral spine **hlt** hook of lateral tooth **ollt** outermost left lateral tooth. Scale bars: 10 μm (**A**); 20 μm (**B, D, E**); 40 μm (**C**), 10 μm (**F**).

**Figure 10.**
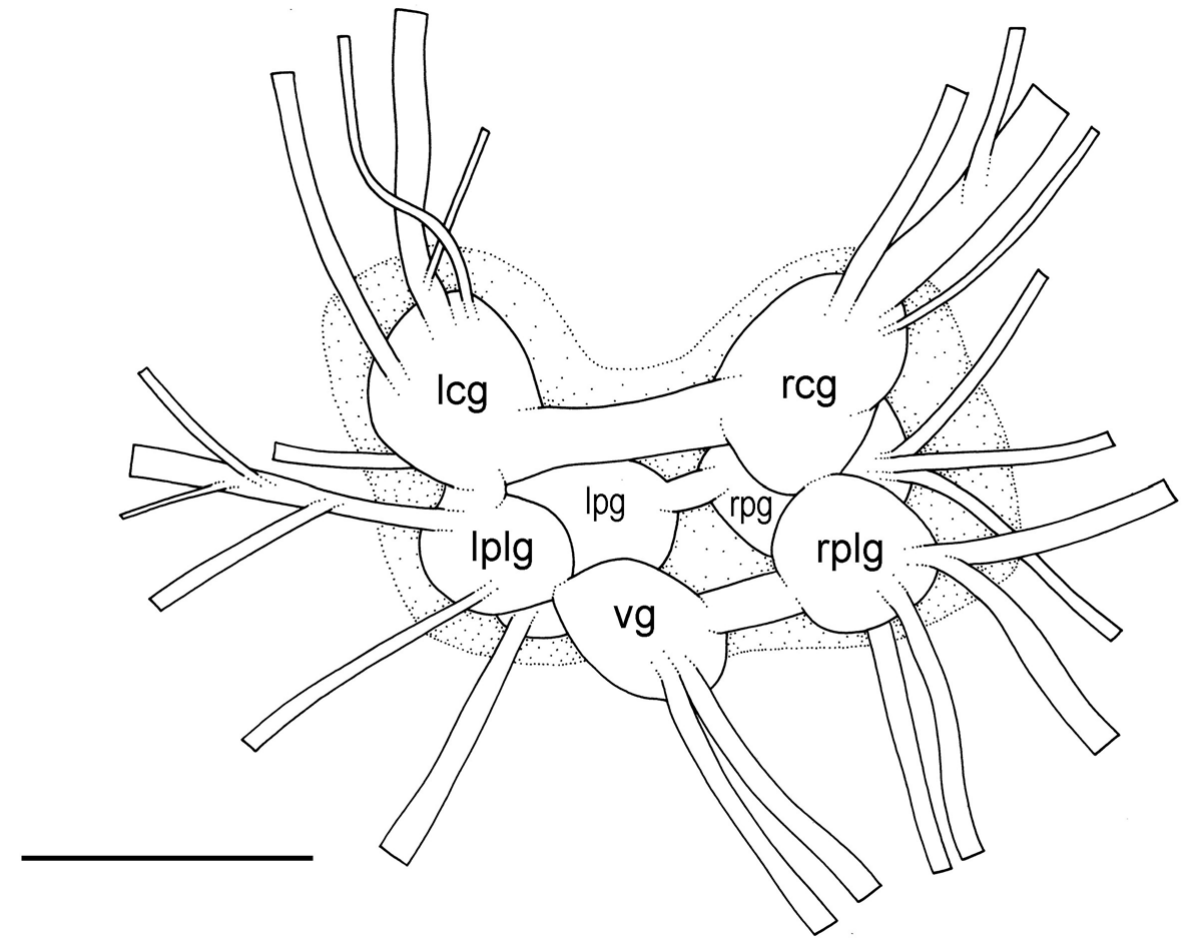
Nervous system, *Wallaconchis sinanui*, holotype, Indonesia, Ambon [2737] (UMIZ 00058) scale bar 0.5 mm. Abbreviations: **lcg** left cerebral ganglion **lpg** left pedal ganglion **lplg** left pleural ganglion **rcg** right cerebral ganglion **rpg** right pedal ganglion **rplg** right pleural ganglion **vg** visceral ganglion.

**Figure 11.**
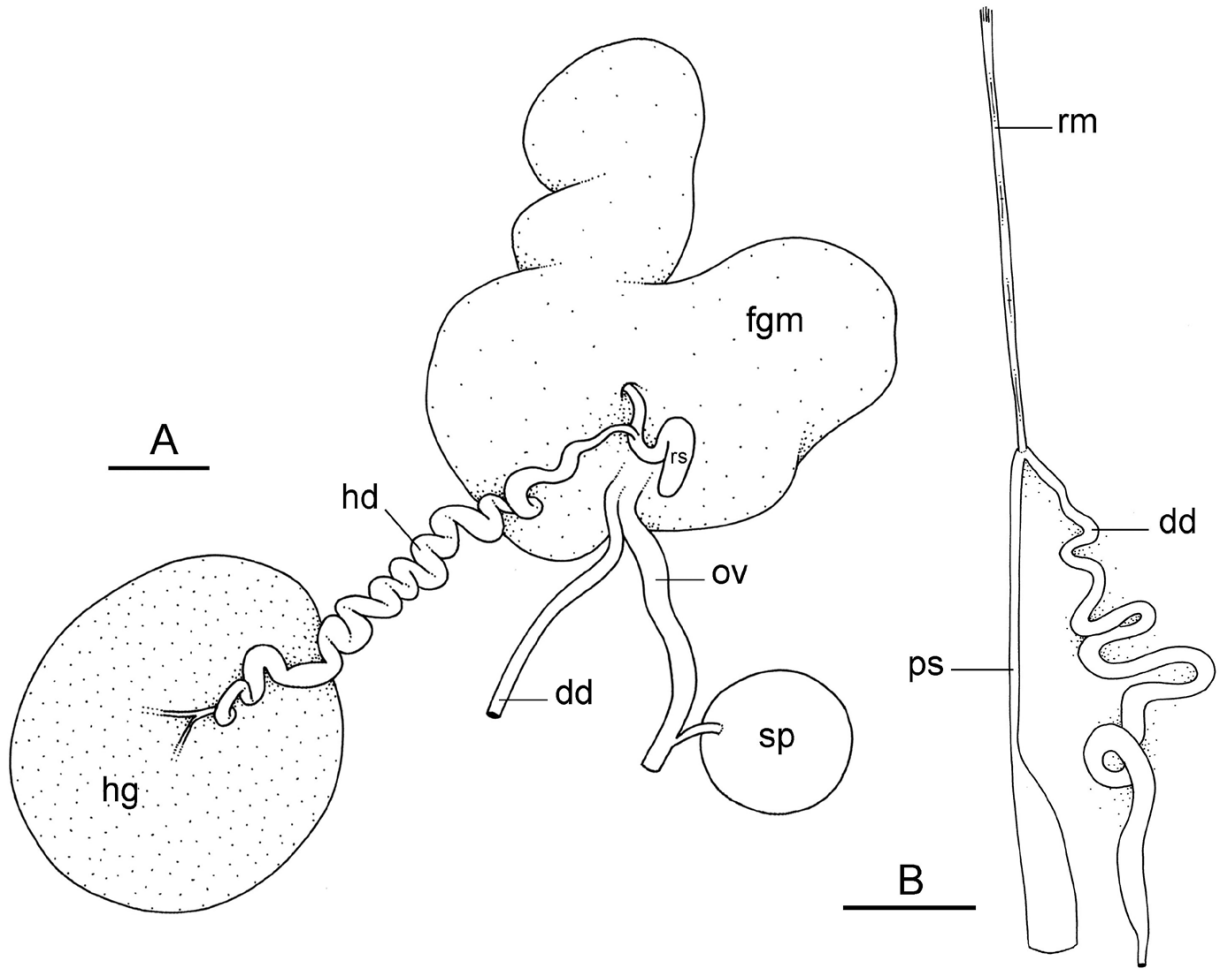
Reproductive system, *Wallaconchis sinanui*, holotype, Indonesia, Ambon [2737] (UMIZ 00058). **A** Hermaphroditic (female), posterior parts, scale bar 0.8 mm **B** Anterior, male copulatory parts, scale bar 2 mm. Abbreviations: **dd** deferent duct **fgm** female gland mass **hd** hermaphroditic duct **hg** hermaphroditic gland **ov** oviduct **ps** penial sheath **rm** retractor muscle **rs** receptaculum seminis **sp** spermatheca **v** vestibule.

**Figure 12.**
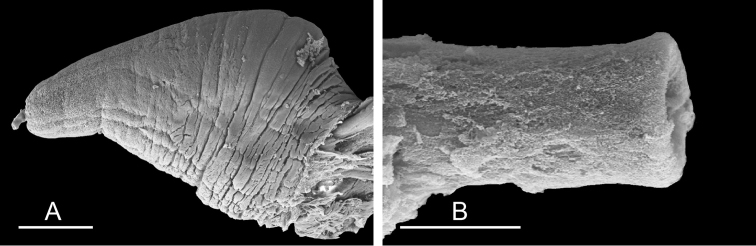
Penis, *Wallaconchis sinanui*, Indonesia, Ambon. **A** Penis evaginated outside the male opening, scale bar 200 μm [5846] (UMIZ 00059) **B** Penis removed from vestibule, scale bar 20 μm [5844] (UMIZ 00059).

Figs 7 , 8 , 9 , 10 , 11 , 12

##### Type locality.

Indonesia, Ambon, Lateri, 03°38.26'S, 128°14.72'E, st 128, mudflat next to small creek in mangrove preserve.

##### Type material.

Holotype, 9/5 mm [2737], designated here (UMIZ 00058).

##### Additional material examined.

**Indonesia**, Ambon, Lateri, 03°38.26'S, 128°14.72'E, 6 specimens 11/6 mm [5844], 10/4 mm [2738], 9/6 mm [5845], 9/4 mm [2746], 9/4 mm [2740], and 7/5 mm [5846], st 128, mudflat next to small creek in mangrove preserve (UMIZ 00059).

##### Distribution.

Indonesia: Ambon (type locality).

##### Habitat

(Fig. 7A–C, Table 3). *Wallaconchis sinanui* was found in a low intertidal mudflat, by a mangrove. The mud was firm, mixed with small pieces of broken shells, and threads of microalgae. The area in which *W. sinanui* was found was only about 200 square meters, but the density of slugs was extremely high (dozens of slugs per square meter). This species may need this highly specific substrate because it was not found at any other locality in Ambon or in the nearby island of Seram (Indonesia).

##### Etymology.

*Wallaconchis sinanui* is dedicated to Dominggus Ledrick Sinanu, the guard of the protected mangrove forest in Lateri who advocated for its conservation. This mangrove forest is one of the few left in Ambon, and the only locality where this species was found.

##### Diagnosis

(Table 5). Externally, *Wallaconchis sinanui* has been observed with a unique dorsal color pattern. However, samples from additional populations are needed to determine if this dorsal coloration is diagnostic of the species or varies between populations. Additionally, *W. sinanui* is much smaller than most other *Wallaconchis* species, with the exception of *W. achleitneri* (which is endemic to Queensland). Internally, the combination of a smooth, short, and narrow penis, a free oviduct (not attached to the body wall by fibers), and a spherical spermatheca distinguishes *W. sinanui* from other *Wallaconchis* species.

##### Color and morphology of live animals

(Fig. 7D–F). Live animals are usually not covered with mud and the color patterns on their dorsum are mostly visible. The head is small and remains covered by the dorsal notum as the animal crawls. The body is not flattened, although some animals appear flattened when crawling. The dorsal notum is oval, and not hard. Its surface, covered with small papillae of variable sizes and colors, is granular and not smooth.

The dorsal notum is mostly brown, usually with two additional dark stripes or a longitudinal dark band. The ocular tentacles are light brown. The hyponotum and the foot are brownish-grey.

##### External morphology

(Fig. 8A–C). Dorsal gills are absent. Dorsal papillae with so-called ‘dorsal eyes’ are present, with typically three or four eyes per papilla (Fig. 8A). There usually are four or five papillae with dorsal eyes, even though their exact number is difficult to determine because they can be retracted within the notum. The papillae with dorsal eyes are located on the center of the notum. There is a retractable papilla with eyes in the center of the dorsal notum, which is not raised above the other papillae. The foot is wide relative to the hyponotum (between 1/2 and 3/4 of the total width), while in relaxed individuals the foot may even be as wide as the animal. The pneumostome is median (i.e., in line with the anus) and close to the edge of the pedal sole (Fig. 8C). Its position on the hyponotum relative to the notum margin and the edge of the pedal sole varies among individuals between the middle and closer to the notum margin. The anus is posterior, median, and very close to the lateral wall of pedal sole (Fig. 8C). On the right side (to the left in ventral view), a peripodial groove is present at the junction between the pedal sole and the hyponotum, running longitudinally from the buccal area to the posterior end, and ending with the female opening. The female opening is posterior, located approximately 0.5 – 1 mm from the anus depending on the size of the animal (farther from the anus in larger animals). The male aperture (opening of the penis) is anterior, located below the right ocular tentacle (Fig. 8B). The position of the male aperture varies little between individuals.

##### Visceral cavity and pallial complex.

Marginal glands (found in *Onchidella* Gray, 1850) are absent. The visceral cavity is not divided: the heart is not separated from the visceral organs by a thick, muscular membrane (as in *Hoffmannola* Strand, 1932). The heart is enclosed in the pericardium, in the posterior half of the right side of the visceral cavity. The large, anterior, ventricle becomes a large aorta that branches into smaller vessels delivering blood to the visceral organs. The auricle, significantly smaller than the ventricle, is posterior. The pericardium communicates through a small hole with the right portion of the renal-pulmonary complex. The kidney is intricately attached to the pulmonary cavity and is slightly asymmetrical (the right part being slightly larger than the left part).

##### Digestive system

(Figs 8D–F, 9, Table 4). There are no jaws. The left and right salivary glands on either side of the esophagus are heavily branched, joining the buccal mass dorsally. The radula is located between two large postero-lateral muscular masses. Each radular row contains a rachidian tooth and two half rows of lateral teeth. Examples of radular formulae are presented in Table 4. The half rows of lateral teeth form an angle of approximately 45° with the rachidian axis. The rachidian teeth are tricuspid: the median cusp is always present (Fig. 9A); the two lateral cusps, on the lateral sides of the base of the rachidian tooth, are small and inconspicuous (Fig. 9A). The lateral aspect of the base of the rachidian teeth is straight. The length of the main cusp of the rachidian teeth is approximately 15 µm, significantly smaller than that of the lateral teeth. The lateral teeth are unicuspid with a flattened and curved hook (Fig. 9B). The length of the hook of the lateral teeth gradually increases from 25 to 35 µm, along the half row from the inner teeth to the outer teeth, excluding the innermost lateral tooth and several outermost lateral teeth (Fig. 9D, F) which are significantly smaller. The inner and outer lateral aspects of the hook of the lateral teeth are straight (i.e., not wavy and not forming any protuberance). Along a half row, the lateral teeth transition in shape from the inner region, in which the lateral teeth are wide (Fig. 9C), to the outer region, in which the lateral teeth are narrow and closely packed (Fig. 9D, F). The wide lateral teeth of the inner third to half of each half row bear a spine on the lateral expansion of the base, while the narrow lateral teeth in the outer region of each half row lack a basal lateral spine (Fig. 9E, F). In most cases, the basal lateral spine cannot be observed because it is hidden below the hook of the next, outer lateral tooth.

**Table 4.** Radular forumulae in *Wallaconchis* species.

Species	Radular formula	Specimen size (length, in mm)	Catalog Numbers	DNA extraction number
*W. sinanui*	36 × (50-1-50)	11	UMIZ 00059	5844
	34 × (50-1-50)	9	UMIZ 00059	5845
	37 × (50-1-50)	7	UMIZ 00059	5846
*W. uncinus*	59 × (85-1-85)	28	UMIZ 00007	2843
	48 × (65-1-65)	15	UMIZ 00008	3138
	74 × (95-1-95)	40	UMIZ 00011	5079
*W. buetschlii*	58 × (85-1-85)	31	UMIZ 00021	2120
	71 × (105-1-105)	42	MTQ st100	2554
	42 × (70-1-70)	16	MTQ st100	2555
*W. gracile*	50 × (85-1-85)	22	UMIZ 00056	N/A
	44 × (75-1-75)	17	UMIZ 00056	3106
	51 × (65-1-65)	24	PNM 041231	3652
*W. nangkauriense*	74 × (100-1-100)	42	UMIZ 00012	N/A
	59 × (80-1-80)	18	UMIZ 00020	3129
	74 × (105-1-105)	28	PNM 041206	3276
*W. ater* unit #1	71 × (113-1-113)	33	UMIZ 00038	2283
	67 × (96-1-96)	25	UMIZ 00039	2330
	43 × (55-1-55)	17	UMIZ 00041	2939
*W. ater* unit #2	65 × (90-1-90)	29	UMIZ 00050	2220
	55 × (80-1-80)	22	UMIZ 00053	3137
	60 × (90-1-90)	30	PNM 041222	3270
*W. graniferum* unit #1	64 × (105-1-105)	46	PNM 041227	3163
	60 × (95-1-95)	28	PNM 041228	3636
	59 × (95-1-95)	18	PNM 041228	5760
*W. graniferum* unit #2	60 × (85-1-85)	26	PNM 041228	3638
	58 × (85-1-85)	16	PNM 041228	5762
*W. achleitneri*	37 × (70-1-70)	14	MTQ st117	N/A
	38 × (65-1-65)	8	MTQ st117	3535
	36 × (60-1-60)	7	MTQ st117	3534
*W. comendadori*	47 × (95-1-95)	7	UMIZ 00061	3539
	52 × (115-1-115)	18	UMIZ 00063	2985
	51 × (105-1-105)	22	PNM 041234	3385
*W. melanesiensis* unit #1	58 × (130-1-130)	13	UMIZ 00068	2733
	68 × (175-1-175)	43	UMIZ 00068	2735
	62 × (105-1-105)	23	UMIZ 00069	5132
*W. melanesiensis* unit #2	47 × (150-1-150)	27	UMIZ 00070	2963

The esophagus is narrow and straight; its internal folds cannot be seen externally. The esophagus enters the stomach anteriorly. The stomach is located on the left dorsal side of the visceral mass. In dorsal view, only a portion of its posterior aspect can be seen because it is partly covered by the lobes of the digestive gland. The dorsal lobe is mainly on the right, the left, lateral lobe is mainly ventral, and the posterior lobe covers the posterior aspect of the stomach. The stomach is a U-shaped sac divided into four chambers (Fig. 8E, F). The first chamber, just distal to the esophagus, is delimited by a thin layer of tissue, and receives the ducts of the dorsal and left lateral lobes of the digestive gland. The second chamber is delimited by a thick, muscular layer of tissue, and receives the duct of the posterior lobe of the digestive gland. In the third chamber of the stomach are thick ridges which extend towards the middle of the chamber. The fourth chamber is externally similar to the third chamber but is characterized by much lower and thinner internal ridges. The intestine is long and narrow, with loops of type I (Fig. 8D–F). No rectal gland is present.

##### Nervous system

(Fig. 10). The circum-esophageal nerve ring is post-pharyngeal and pre-esophageal. The cerebral commissure between the two cerebral ganglia is short but its length varies among individuals. Pleural and pedal ganglia are also all distinct. The visceral commissure is short but distinctly present and the visceral ganglion is approximately median. Cerebro-pleural and pleuro-pedal connectives are very short and pleural and cerebral ganglia touch each other. Nerves from the cerebral ganglia innervate the buccal area and the ocular tentacles, and, on the right side, the penial complex. Nerves from the pedal ganglia innervate the foot. Nerves from the pleural ganglia innervate the lateral and dorsal regions of the mantle. Nerves from the visceral ganglia innervate the visceral organs.

##### Reproductive system

(Fig. 11A). Sexual maturity is correlated with animal length. Mature individuals have large, fully-developed, female and male parts. Immature individuals may have small, inconspicuous, or simply no female organs, and rudimentary male parts.

The hermaphroditic gland is a single mass. A hermaphroditic duct conveys the eggs and the autosperm from the hermaphroditic gland to the fertilization chamber, which connects to a large, elongate, usually bent receptaculum seminis (caecum). The shape and size of the receptaculum seminis vary between individuals. The female gland mass contains various glands (mucus and albumen) of which the exact connections remain uncertain. The spermoviduct (for the autosperm, the exosperm, and the eggs) is embedded within the female gland mass, at least proximally. Distally, the spermoviduct branches into the deferent duct (which conveys the autosperm to the anterior region, running through the body wall) and the oviduct. The latter conveys the eggs up to the female opening and the exosperm. The oviduct is short (approximately the same length as the free deferent duct) and almost as narrow as the deferent duct. The spherical spermatheca connects to the narrow distal portion of the oviduct through a short duct.

##### Copulatory apparatus

(Figs 11B, 12). The male anterior organs include the penial complex (penis, vestibule, deferent duct, and retractor muscle). There is no penial accessory gland. The penis (from 55 to 70 µm long) is narrow and smooth with no hooks (Fig. 12). The vestibule is much wider than the narrow penial sheath (Fig. 11B). The distal end of the penis is within the base of the vestibule. The beginning of the retractor muscle for the penis marks the separation between the deferent duct (proximal) and the penial sheath (distal). The deferent duct is approximately the same width as the penis proximally but becomes larger distally. The deferent duct is convoluted but short. The retractor muscle is approximately the same length as the penial sheath, and inserts posteriorly, on the body wall, near the rectum.

#### Wallaconchis uncinus

Taxon classificationAnimaliaSystellommatophoraOnchidiidae

Goulding & Dayrat sp. n.

http://zoobank.org/7AD571DD-A286-4979-90DA-35DFDEFE52B8

**Figure 13.**
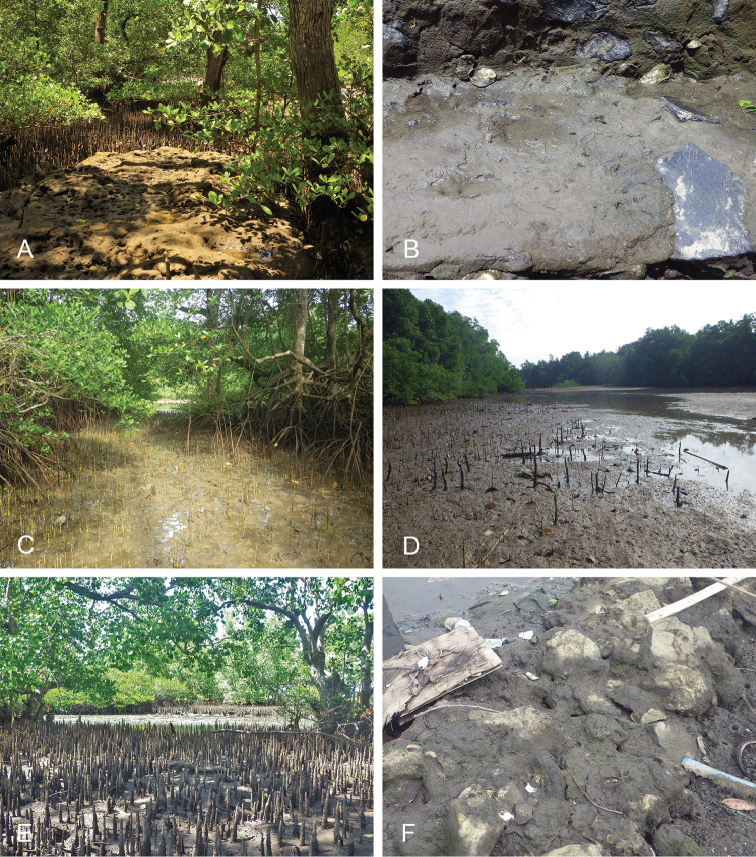
Habitats, *Wallaconchis uncinus*, Indonesia. **A, B** Sulawesi, Wori, tall mangrove forest of *Sonneratia* and some *Avicennia* with a rocky area and dead logs (st 90) **C** Ambon, Lateri, muddy mangrove with *Rhizophora* and *Avicennia* (st 131) **D** Type locality, Ambon, Lateri, mudflat adjacent to a shallow river through a mangrove (st 128) **E** Halmahera, Sofifi, mangrove with *Sonneratia* trees, dense roots and hard mud (st 205) **F** Ternate, Bastiong, muddy rocks nearby a mangrove patch (st 203).

**Figure 14.**
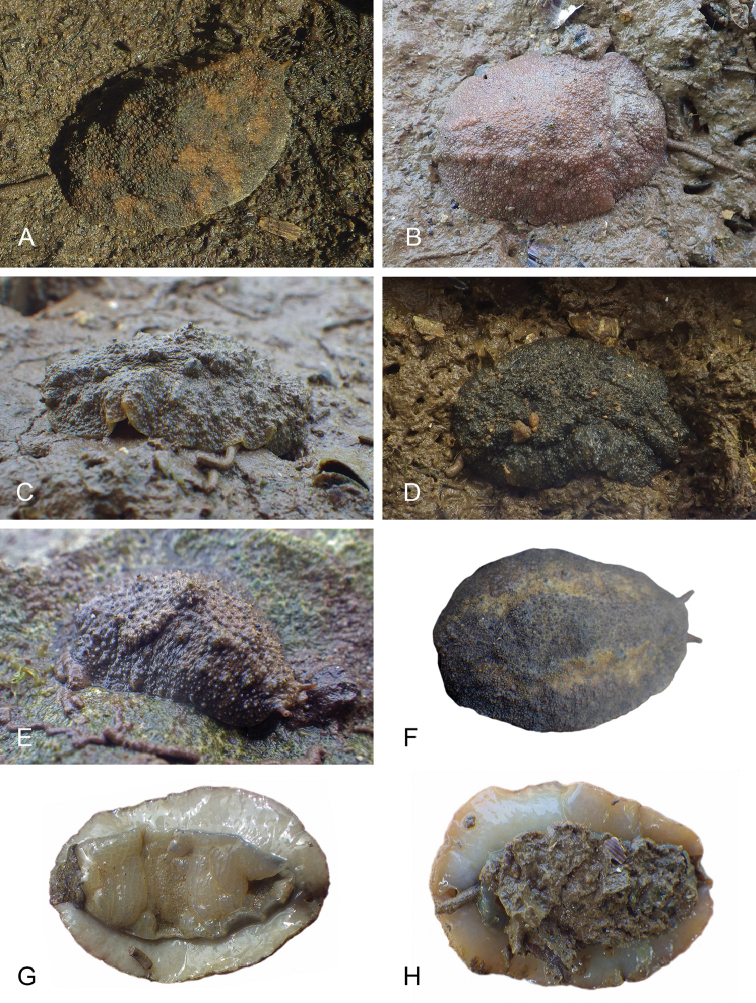
Live specimens, *Wallaconchis uncinus*, Indonesia. **A** Dorsal view, 25 mm long [5070], Halmahera (UMIZ 00010) **B** Holotype, dorsal view, 22 mm long [2751], Ambon (UMIZ 00004) **C** Dorsal view, 30 mm long [2752], Ambon (UMIZ 00006) **D** Dorsal view, 28 mm long [2843], Ambon (UMIZ 00008) **E** Dorsal view, 15 mm long [3138], Bali (UMIZ 00005) **F** Dorsal view, 25 mm long [5080], Halmahera (UMIZ 00011) **G** Ventral view, same as F **H** Ventral view, same as B.

**Figure 15.**
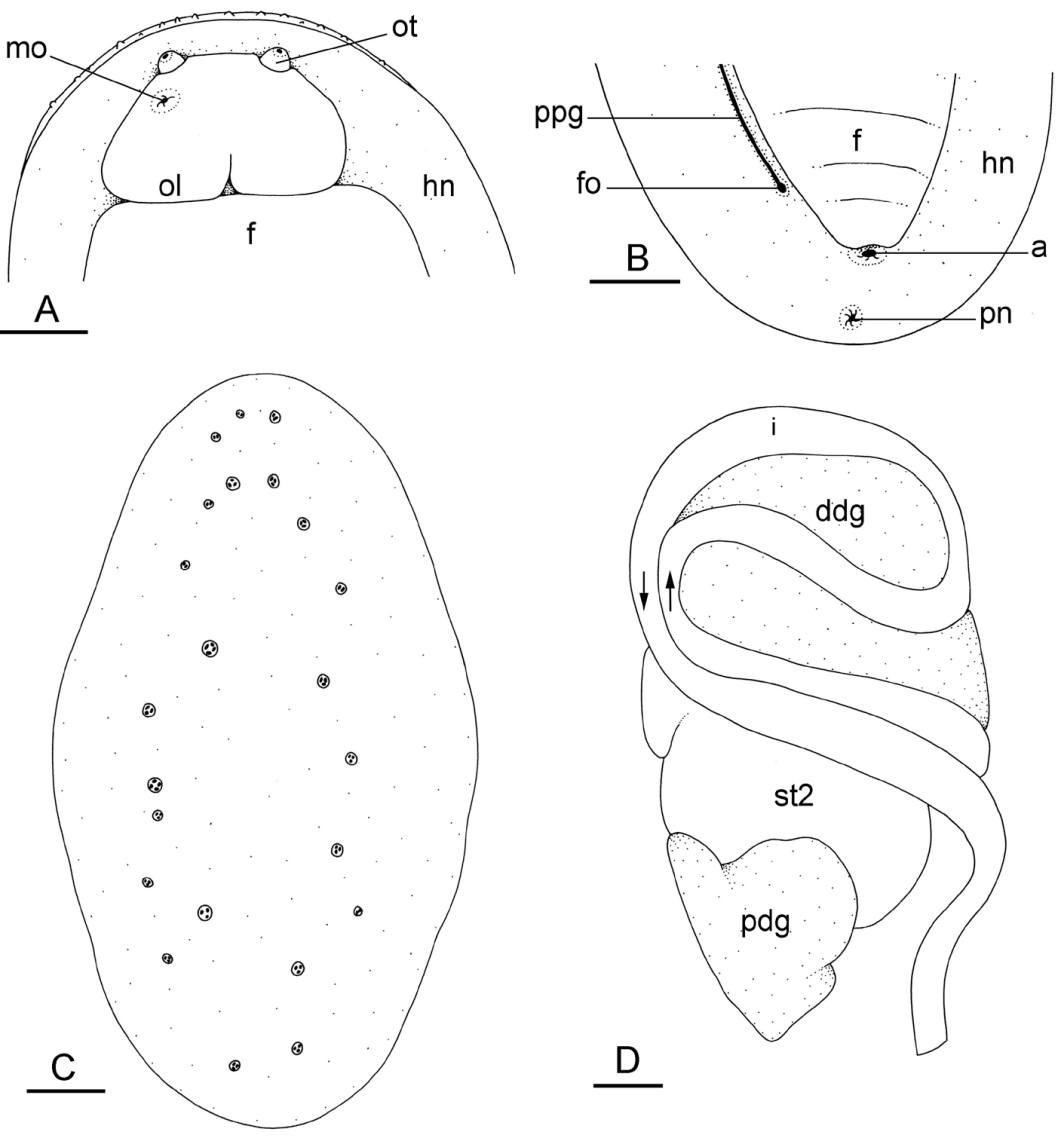
External morphology and digestive system, *Wallaconchis uncinus*, Indonesia. **A** Anterior region, ventral view, Sulawesi, scale bar 2 mm [2268] (UMIZ 00005) **B** Posterior region, ventral view, Sulawesi, scale bar 2 mm [2268] (UMIZ 00005) **C** Dorsal notum (papillae with dorsal eyes), Halmahera, scale bar 2.6 mm [5056] (UMIZ 00009) **D** Digestive system, dorsal view, Sulawesi, scale bar 1 mm [2268] (UMIZ 00005). Abbreviations: **a** anus **ddg** dorsal lobe of digestive gland **f** foot **fo** female opening **hn** hyponotum **i** intestine **mo** male opening **ol** oral lobe **ot** ocular tentacle **pdg** posterior lobe of the digestive gland **pn** pneumostome **ppg** peripodial groove **st2** stomach chamber 2.

**Figure 16.**
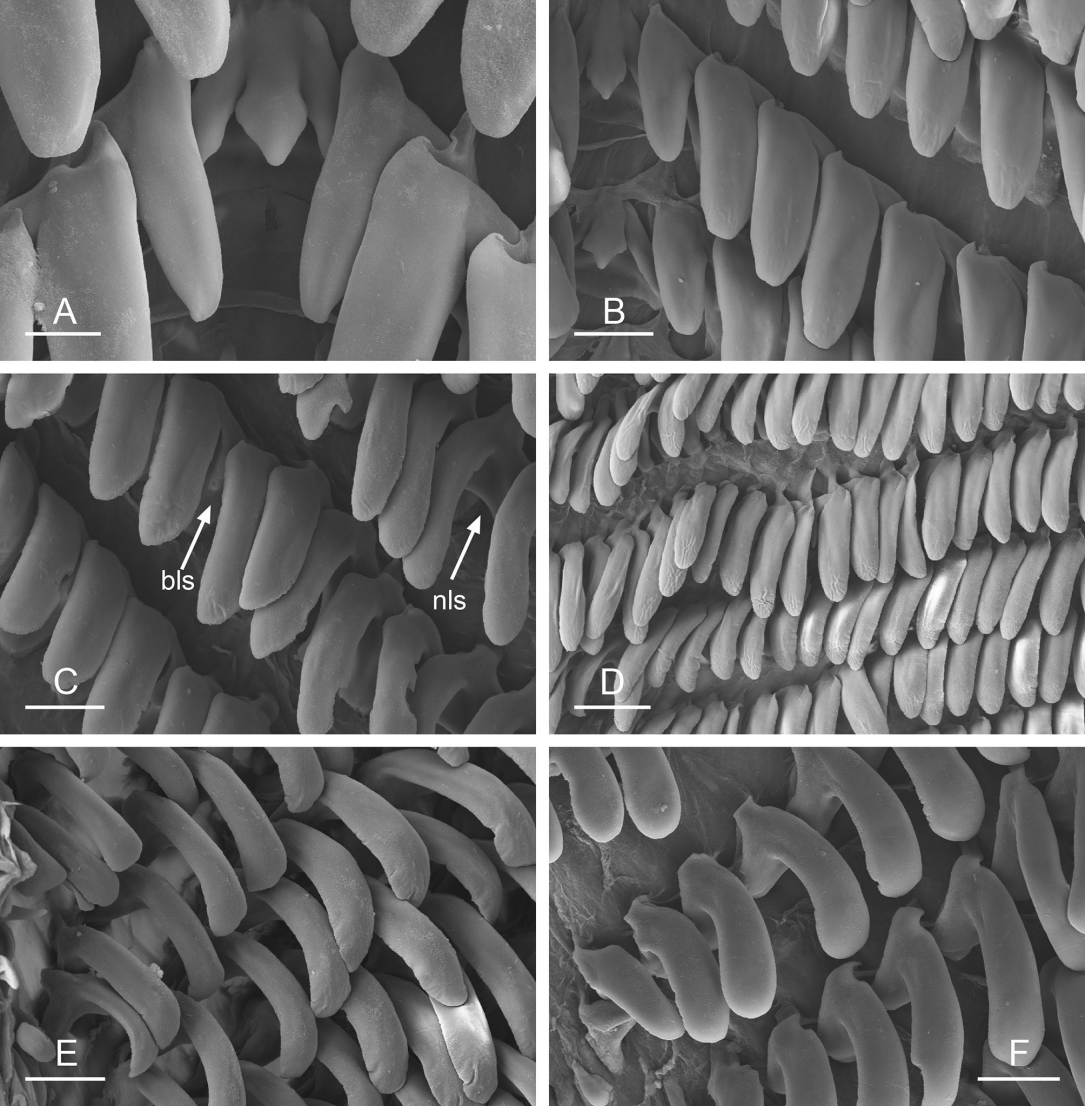
Radula, *Wallaconchis uncinus*, Indonesia. **A** Rachidian and innermost lateral teeth, Bali, scale bar 10 μm [3138] (UMIZ 00008) **B** Rachidian and innermost lateral teeth, Halmahera, scale bar 20 μm [5079] (UMIZ 00011) **C** Transition from inner to outer lateral teeth, scale bar 20 μm, same as B **D** Outer lateral teeth, scale bar 40 μm, same as B **E** Outermost lateral teeth, scale bar 20 μm, same as B **F** Outermost lateral teeth, Ambon, scale bar 20 μm [2843] (UMIZ 00007). Abbreviations: **bls** basal lateral spine (of lateral tooth) **nls** no lateral spine.

**Figure 17.**
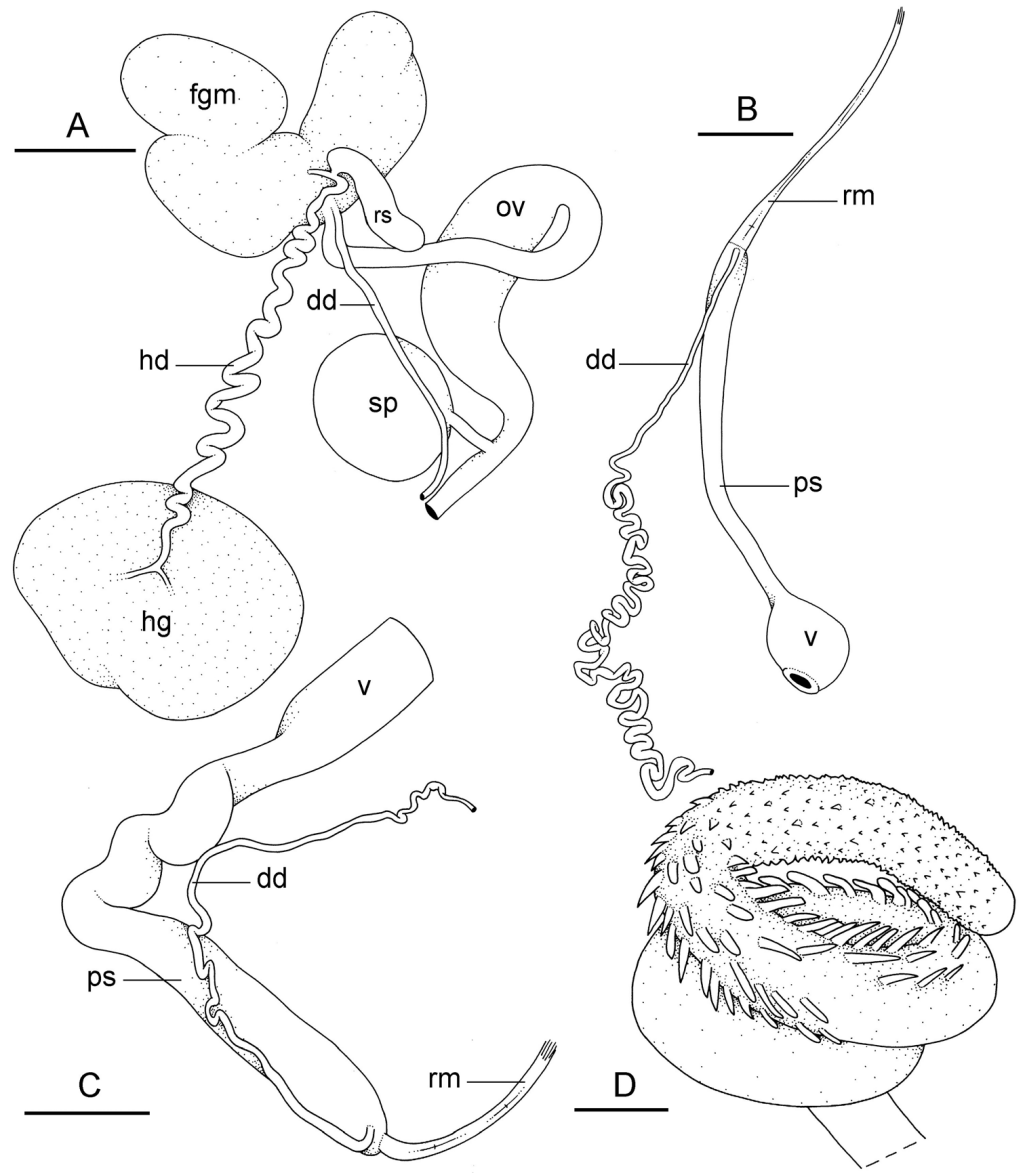
Reproductive system, *Wallaconchis uncinus*, Indonesia. **A** Hermaphroditic (female), posterior parts, Sulawesi, scale bar 2 mm [2268] (UMIZ 00005) **B** Anterior, male copulatory parts, Halmahera, scale bar 3 mm [5079] (UMIZ 00011) **C** Anterior, male copulatory parts, Bali, scale bar 1 mm [3138] (UMIZ 00008) **D** Penis (fully evaginated), Halmahera, scale bar 0.5 mm [5079] (UMIZ 00011). Abbreviations: **dd** deferent duct **fgm** female gland mass **hd** hermaphroditic duct **hg** hermaphroditic gland **ov** oviduct **ps** penial sheath **rm** retractor muscle **rs** receptaculum seminis **sp** spermatheca **v** vestibule.

**Figure 18.**
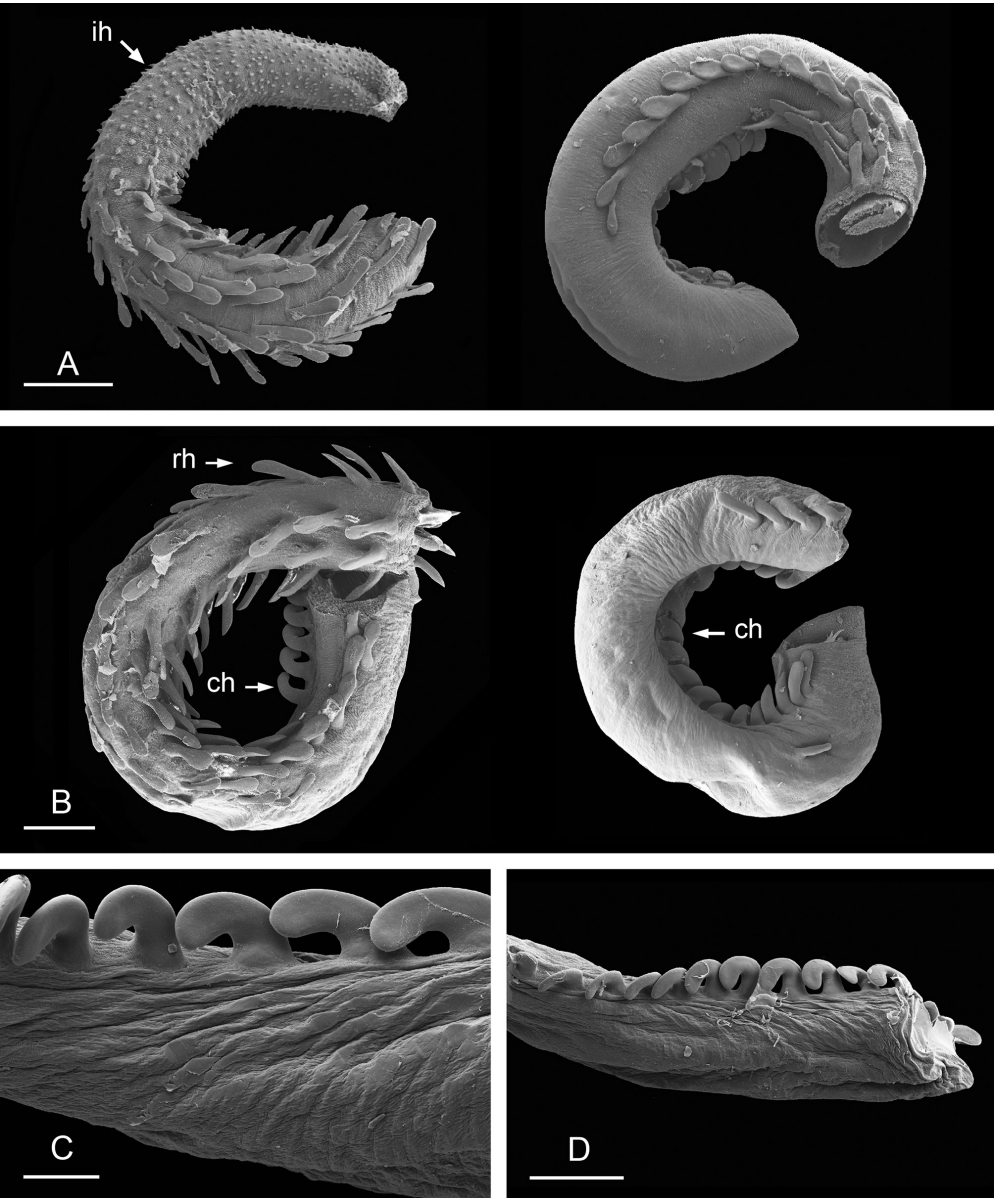
Penis, *Wallaconchis uncinus*, Indonesia. **A** Two most distal loops of fully evaginated penis (the loop on the right is proximal to the loop on the left), Halmahera, scale bar 0.5 mm [5079] (UMIZ 00011) **B** Two most distal loops of (nearly fully evaginated) penis (the loop on the right is proximal to the loop on the left), Ambon, scale bar 300 μm [2843] (UMIZ 00007) **C** Proximal region of penis, detail view of curved hooks, Sulawesi, scale bar 100 μm [2268] (UMIZ 00005) **D** Distal region of penis which is not fully evaginated, scale bar 300 μm, same as C. Abbreviations: **ch** curved hooks **ih** immature hooks **rh** rounded hooks.

Figs 13 , 14 , 15 , 16 , 17 , 18

##### Type locality.

Indonesia, Ambon, Lateri, 03°38.26'S, 128°14.72'E, st 128, mudflat next to small creek in the low intertidal of mangrove preserve.

##### Type material.

Holotype, 22/17 mm [2751], designated here (UMIZ 00004).

##### Additional material examined.

**Indonesia**, North Sulawesi, Wori, 01°36.06'N, 124°51.73'E, 4 specimens 22/11 mm [2256], 22/16 mm [2250], 18/7 mm [2268], 17/7 mm [2261], st 90, old *Avicennia*, *Sonneratia*, *Rhizophora* mangrove forest with, rocks and dead logs (UMIZ 00005); Ambon, Lateri, 03°38.26'S, 128°14.72'E, 1 specimen 30/21 mm [2752], st 128, mudflat next to small creek and mangrove (UMIZ 00006); Ambon, Lateri, 03°38.24'S, 128°14.78'E, 1 specimen 28/17 mm [2843], st 131, muddy *Rhizophora* mangrove (UMIZ 00007); Bali, Pemuteran, Labuhan Lalang Harbor, 08°08.61'S, 114°32.33'E, 1 specimen 15/13 mm [3138], st 157, coral rubble, rocks and a few *Avicennia* (UMIZ 00008); North Maluku, Ternate, Bastiong, 00°46.41'N, 127°22.76'E, 1 specimen (24/11 mm [5056]), st 203, muddy rocks near a mangrove (UMIZ 00009); Halmahera, Sofifi, 00°45.47'N, 127°35.90'E, 2 specimens 25/16 mm [5070] and 22/16 mm [5029], st 205, *Sonneratia* mangrove (UMIZ 00010); Halmahera, Akelamo, 01°01.33'N, 127°39.09'E, 2 specimens 40/25 mm [5079] and 35/30 mm [5080], st 207, sandy-muddy beach at margin of mangrove (UMIZ 00011); Timor, Oesapa, Kupang City, 10°08.73'S, 123°38.10'E, 1 specimen 29/18 mm [5900], st 250, open *Sonneratia* mangrove (UMIZ 00071).

##### Distribution.

Indonesia: Ambon (type locality), Bali, Halmahera, northern Sulawesi, and Timor.

##### Habitat

(Fig. 13, Table 3). *Wallaconchis uncinus* predominantly lives on firm mud, in and around mangroves. It can also sometimes be found on rocks inside or adjacent to muddy mangroves. It lives in the low to mid-intertidal zone (i.e., not in higher and dry areas only submerged at the highest tides) but is not found on water-saturated mud (unlike onchidiid species in other genera). *Wallaconchis uncinus* frequently co-occurs with *W. buetschlii*. It was also found in the same microhabitat as *W. sinanui* but in much lower abundance (one individual of *W. uncinus* was found on a patch of mudflat amongst dozens of individuals of *W. sinanui*).

##### Etymology.

From the Latin adjective *uncinus* meaning “hooked”, to refer to the distinctive hooks that the penis bears.

##### Diagnosis

(Table 5). *Wallaconchis uncinus* cannot be distinguished from other *Wallaconchis* species based on external features. Red individuals occur in at least three other species (i.e., *W. nangkauriense*, *W. buetschlii*, and *W. ater*). Individuals of *W. uncinus* with orange and yellow bands could be confused with *W. gracile* or *W. buetschlii*, and these color patterns also occasionally occur in onchidiid species of other genera (personal observation). Brown individuals of *W. uncinus* cannot be distinguished from brown individuals of other *Wallaconchis* species. Internally, the flattened hooks (both the curved hooks and the rounded hooks) can be used for identification because they are not present in any other known onchidiid species.

**Table 5.** Summary of traits that can help identify *Wallaconchis* species. Observations between parentheses are less common. All traits may be subject to individual variation. Traits are described in detail in the corresponding species descriptions.

Species	Dorsal color	Hyponotum color	Penis	Oviduct
*W. sinanui*	Brown with dark brown mottling	Brownish-grey	Narrow, within penial sheath	Narrow
*W. uncinus*	Brown, mottle brown and orange (red, black)	Light grey, cream, light orange	Loops with curved hooks, in vestibule	Wide
*W. buetschlii*	Grey-brown (red, yellow-brown)	Dark grey, light grey, cream	No penial sheath. Internal longitudinal ridges	Narrow, in membrane attached to body wall
*W. gracile*	Brown (yellow, brown with patches of yellow, orange, or red)	Light yellow-orange	Narrow tube within penial sheath	Narrow, in membrane attached to body wall
*W. nangkauriense*	Extremely variable: grey, red, yellow, black, orange, brown, green	Light grey, cream (dark grey, white)	Narrow tube within vestibule	Extremely long, slightly narrow
*W. ater*	Extremely variable: grey, red, yellow, black, brown, green	Grey, cream, yellow-grey, yellow-orange	Loops form a coil in vestibule	Wide
*W. graniferum*	Brown, orange, yellow	Beige-orange, cream or white	Bears straight hooks, in vestibule	Wide
*W. achleitneri*	Brown-grey	Light yellow-grey	Narrow, within vestibule	Slightly narrow
*W. comendadori*	Brown, black (with patches brown, yellow, grey, red, black)	Yellow-orange, grey, or both	Narrow, within penial sheath	Narrow
*W. melanesiensis*	Grey, black (red-black), brown	Light grey, bluish-grey	Narrow, within penial sheath	Narrow

##### Color and morphology of live animals

(Fig. 14). The dorsal color is variable but is often completely brown or brown mottled with dark orange or bright yellow. Occasionally, specimens are entirely red or black. The color of the hyponotum ranges from light grey to cream and orange in color. The color of the foot is yellow-grey or light grey. The brownish-orange ocular tentacles are short and extend for only a few mm beyond the margin of the notum when the animal crawls undisturbed.

##### External morphology

(Fig. 15A–C). There usually are between six and 12 papillae with dorsal eyes (even though their exact number is difficult to determine because they can be retracted within the notum). Exceptionally, 23 papillae were observed in one preserved specimen from Halmahera (Fig. 15C). The female opening is posterior, located 3 – 5 millimeters from the anus (Fig. 15B) depending on the size of the animal (farther from the anus in larger animals). The male aperture is located below the right ocular tentacle (Fig. 15A)

##### Digestive system

(Figs 15D, 16, Table 4). Examples of radular formulae are presented in Table 4. The length of the main cusp of the rachidian teeth is approximately 20 µm, significantly smaller than that of the lateral teeth. The length of the hook of the lateral teeth gradually increases from 40 to 55 µm, excluding the innermost lateral tooth and several outermost lateral teeth, which are significantly smaller. The intestinal loops are of type I (Fig. 15D).

##### Reproductive system

(Fig. 17A). The middle portion of the oviduct is much wider than the proximal and distal ends (Fig. 17A). The spherical spermatheca connects to the narrow distal portion of the oviduct through a short duct.

##### Copulatory apparatus

(Figs 17B–D, 18). The penial sheath is long: the length varies between 1/2 of the length of the visceral cavity to nearly the full length of the cavity. The vestibule is approximately 3 mm in length (in mature specimens). The vestibule is wider than the penial sheath and appears cylindrical or spherical depending on the orientation of the penis inside (Fig. 17B). Inside the vestibule, the penis can form multiple circular loops (Fig. 17D), a single loop, or simply be U-shaped. In mature specimens, the penis bears many large flattened hooks of two types: rounded hooks which lay flat on the surface of the penis and curved hooks which project perpendicularly from the surface of the penis (see hook labels, Fig. 18B–D). In the distal region of the penis there may also be immature hooks which are not fully developed (Fig. 18A); these small hooks may also be seen in immature specimens, or they may be absent. The developed hooks differ between regions of the penis: in the distal region, the rounded hooks are spread around the penis; in the middle region, the rounded hooks form a single row (Fig. 18A–B), some of these hooks being adjacent to a row of curved hooks; and, in the proximal region, rounded hooks are generally absent, but the curved hooks may be present. The deferent duct is highly convoluted with many loops (Fig. 17B) (though the deferent duct is significantly less convoluted in immature specimens, see Fig. 17C). The retractor muscle inserts posteriorly, on the body wall, near the rectum.

#### Wallaconchis buetschlii

Taxon classificationAnimaliaSystellommatophoraOnchidiidae

(Stantschinsky, 1907) comb. n.

**Figure 19.**
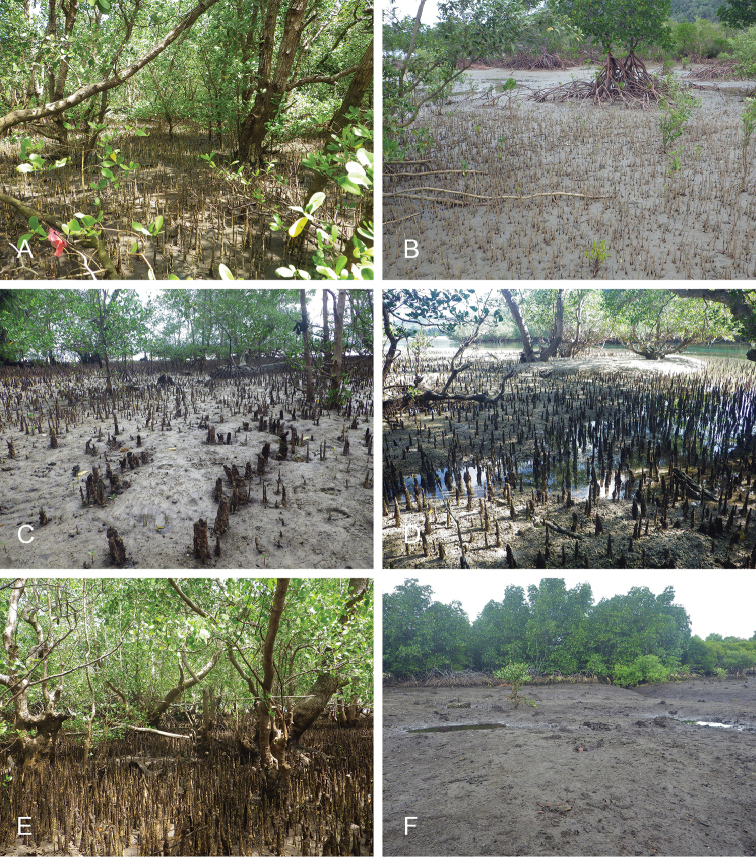
Habitats, *Wallaconchis buetschlii*. **A** Indonesia, Sulawesi, Wori, tall mangrove forest of *Sonneratia* and some *Avicennia* with a rocky area and dead logs (st 84) **B** Australia, Queensland, Yule Point, sandy area with a few *Rhizophora* and *Avicennia* trees (st 100) **C** Indonesia, Ambon, Wai, narrow band of old *Avicennia* trees on sandy mud, with old logs on ground (st 132) **D** Indonesia, Lombok, Don Don, old *Avicennia* forest, with coral rubble (st 149) **E** Indonesia, Sulawesi, Mantehage Island, rocks behind a mangrove of *Sonneratia* and *Rhizophora* (st 91) **F** Indonesia, Bali, Gilimanuk, sandy mudflat outside *Rhizophora* and *Avicennia* mangrove (st 156).

**Figure 20.**
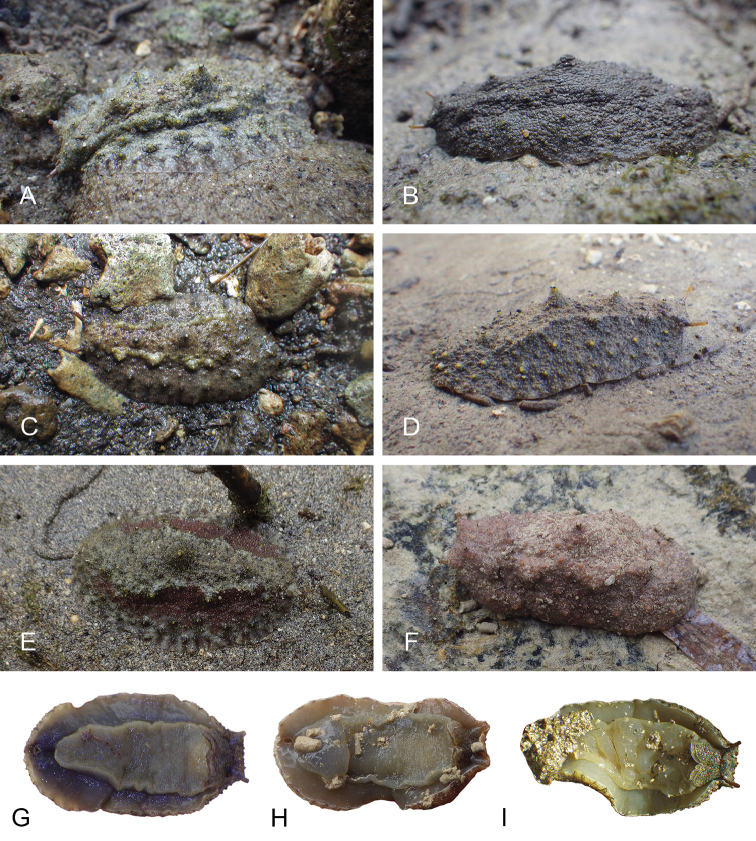
Live specimens, *Wallaconchis buetschlii*. **A** Dorsal view, 46 mm long [2722], Indonesia, Ambon (UMIZ 00026) **B** Dorsal view, 31 mm long [2853], Indonesia, Ambon (UMIZ 00027) **C** Dorsal view, 23 mm long [2249], Indonesia, Sulawesi (UMIZ 00024) **D** Dorsal view, 30 mm long [2928], Indonesia, Kei Islands (UMIZ 00029) **E** Dorsal view, 52 mm long [3159], Philippines, Luzon (PNM 041209) **F** Dorsal view, 36 mm long, [3409], Philippines, Bohol (PNM 041212) **G** Ventral view, 44 mm long [5898], Indonesia, Timor (UMIZ 00072) **H** Ventral view, same as F **I** Ventral view, 43 mm long [2982], Indonesia, Lombok (UMIZ 00030).

**Figure 21.**
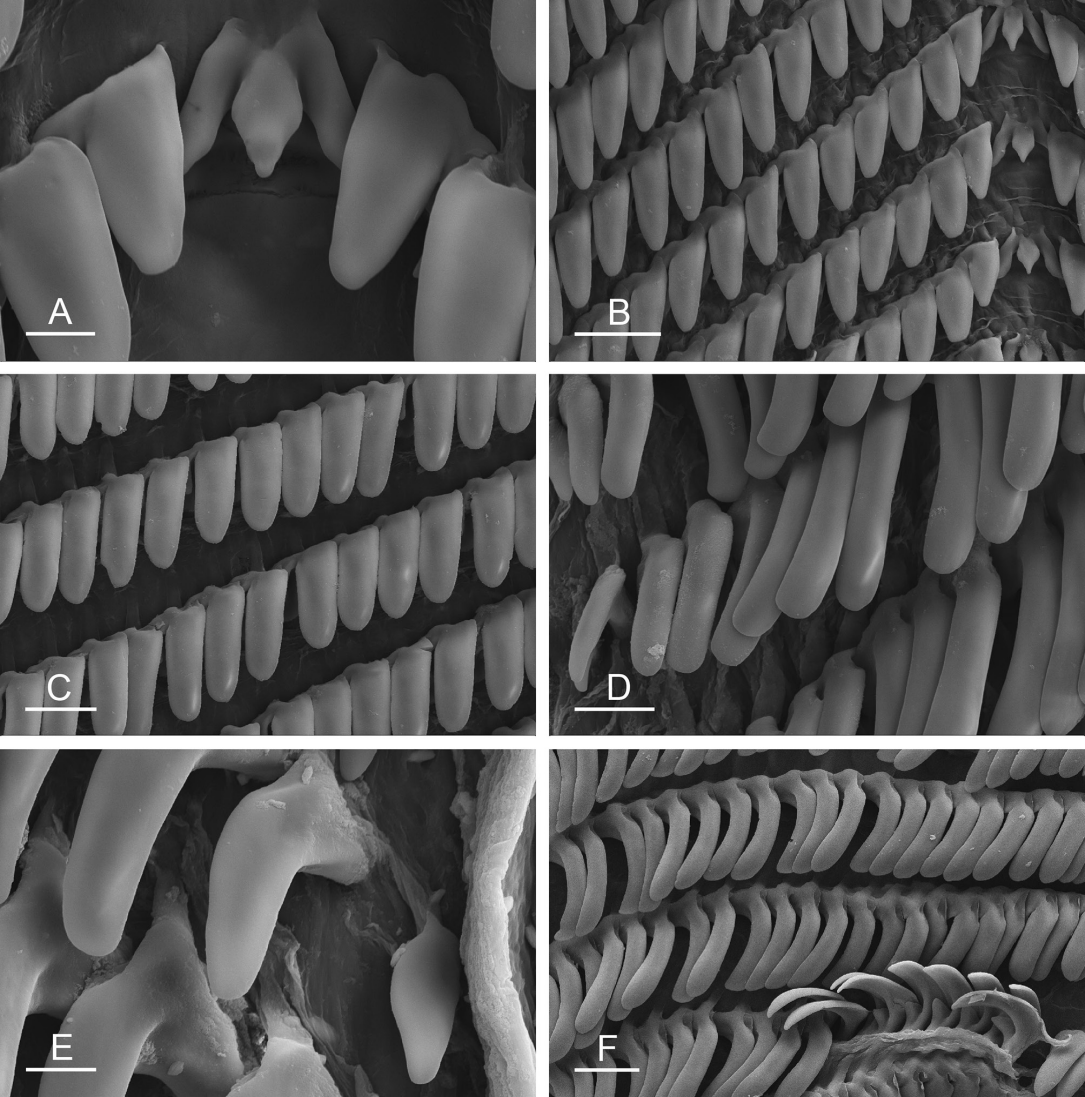
Radula, *Wallaconchis buetschlii* (A–D) Indonesia, Sulawesi [2120] (UMIZ 00021) (E) Australia, Queensland [2554] (MTQ st100) (F) Australia, Queensland [2555] (MTQ st100). **A** Rachidian and innermost lateral teeth, scale bar 10 μm **B** Rachidian and innermost lateral teeth, scale bar 40 μm **C** Inner lateral teeth, scale bar 30 μm **D** Outermost lateral teeth, scale bar 20 μm **E** Outermost lateral teeth, scale bar 10 μm **F** Transition between inner lateral teeth and outer lateral teeth, with the basal lateral spine revealed on the underside of the inner lateral teeth at the bottom, scale bar 30 μm.

**Figure 22.**
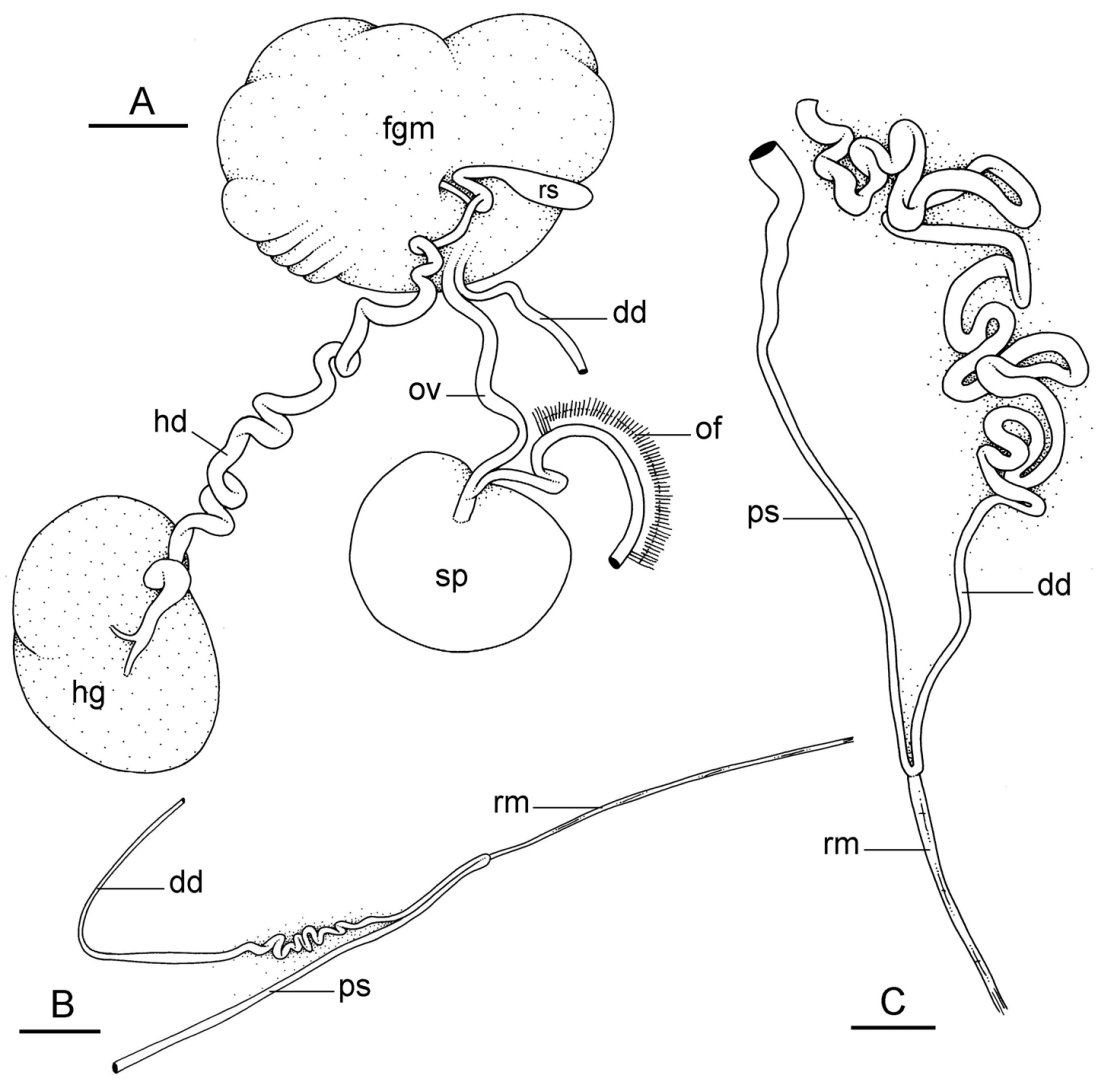
Reproductive system, *Wallaconchis buetschlii*. **A** Hermaphroditic (female), posterior parts, Indonesia, Sulawesi, scale bar 2 mm [2120] (UMIZ 00021) **B** Anterior, male copulatory parts, Australia, Queensland, scale bar 1 mm [2555] (MTQ st100) **C** Anterior, male copulatory parts, Indonesia, Kei, scale bar 1 mm [2933] (UMIZ 00029). Abbreviations: **dd** deferent duct **fgm** female gland mass **hd** hermaphroditic duct **hg** hermaphroditic gland **of** oviduct fibers attaching to the inner wall of the visceral cavity **ov** oviduct **ps** penial sheath **rm** retractor muscle **rs** receptaculum seminis **sp** spermatheca.

**Figure 23.**
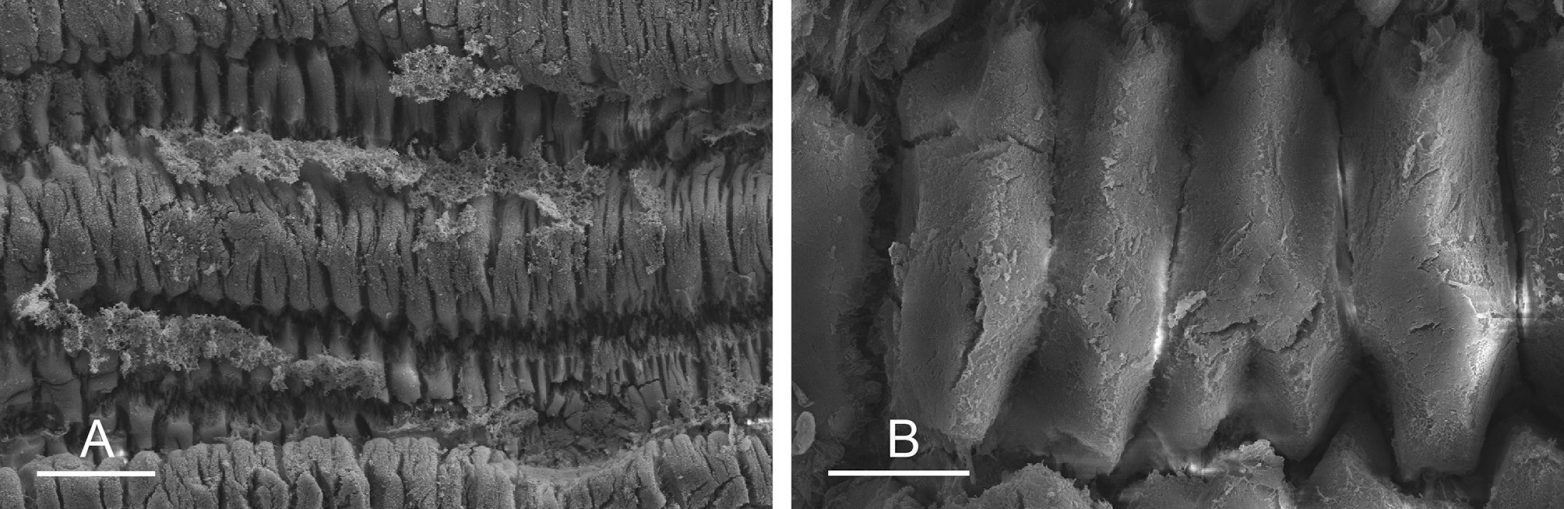
Penis, *Wallaconchis buetschlii*, Indonesia. **A** Longitudinal ridge on the internal surface of the penis, Kei Islands, scale bar 60 μm [2933] (UMIZ 00029) **B** Close-up of a groove between two longitudinal ridges showing smooth internal surface (no hooks), Sulawesi, scale bar 20 μm [2120] (UMIZ 00021).

Figs 19 , 20 , 21 , 22 , 23

Onchidium buetschlii Stantschinsky, 1907: 383–386, pl. 12, figs 10–12, pl. 13, figs 20a, 20b, 22, 35; Bretnall 1919: 316 (as Oncidium buetschlii).
Onchidium gracile : Stantschinsky 1907: pl. 13, fig. 34 [non Onchidium gracile Stantschinsky, 1907].

##### Type locality.

Australien: Queensland [Queensland, Australia]. Stantschinsky indicated that the syntypes were collected from the state of Queensland by Professor Simroth, but no detailed information about the locality is available in either the original description or the jar of the type material. Fresh material was collected from the type locality (see below, additional material).

##### Type material.

Lectotype, 19/17 mm, designated here (SMF 333595/2). The lectotype was dissected prior to the present study and its posterior end is missing. A lectotype is designated here because its remaining anterior, male parts can be used for species identification. The two other syntypes (30/22 mm and 30/29 mm) become paralectotypes (SMF 333595/2). Both paralectotypes were dissected prior to the present study and their male parts are missing. According to the original description, there were four syntypes, so one syntype has been lost or destroyed.

##### Additional material examined.

**Indonesia**, North Sulawesi, Wori, 01°36.06'N, 124°51.73'E, 2 specimens 31/14 mm [2120] and 27/14 mm [2122], st 84, old *Sonneratia* and *Avicennia* mangrove (UMIZ 00021); North Sulawesi, Bahoi, 01°43.36'N, 125°01.23'E, 1 specimen 22/18 mm [2189], st 85, sand and small rocks outside a mangrove (UMIZ 00022); North Sulawesi, Wori, 01°36.06'N, 124°51.73'E, 1 specimen 23/14 mm [2249], st 90, old mangrove forest with *Avicennia*, *Sonneratia*, and *Rhizophora*, with rocks (UMIZ 00024); North Sulawesi, Mantehage Island, 01°41.88'N, 124°46.74'E, 1 specimen 32/22 mm [2310], st 91, rocks behind a mangrove of *Sonneratia* and *Rhizophora* (UMIZ 00025); Ambon, Haruku Island, 03°36.52'S, 128°25.07'E, 1 specimen 46/21 mm [2722], st 127, rocky *Sonneratia* mangrove with coral rubble (UMIZ 00026); Ambon, Wai, 03°34.65'S, 128°19.53'E, 1 specimen 31/16 mm [2853], st 132, narrow band of old *Avicennia* trees on sandy mud, old logs on ground (UMIZ 00027); Seram, 02°58.24'S, 128°07.07'E, 1 specimen 21/17 mm [2872], st 135, mud next to a mangrove (UMIZ 00028); Maluku, Kei Islands, Tual City, Fiditan, 05°35.96'S, 132°45.11'E, 2 specimens 30/16 mm [2928] and 30/15 mm [2933], st 144, rocks behind muddy mangrove of *Rhizophora* (UMIZ 00029); Lombok, Don Don, 08°54.54'S, 116°21.50'E, 2 specimens 43/23 mm [2982] and 43/23 mm [2989] st 149, old, *Avicennia* forest with coral rubble (UMIZ 00030); Bali, Gilimanuk, 08°10.16'S, 114°26.65'E, 1 specimen 24/15 mm [3123], st 156, sandy mudflat outside *Rhizophora* and *Avicennia* mangrove (UMIZ 00032); Bali, Pemuteran, Labuhan Lalang Harbor, 08°08.61'S, 114°32.33'E, 1 specimen 27/17 mm [3128], st 157, coral rubble, rocks and mud with a few *Avicennia* (UMIZ 00033); North Maluku, Ternate, Bastiong, 00°46.41'N, 127°22.76'E, 1 specimen 32/19 mm [5016], st 203, muddy rocks near a mangrove (UMIZ 00034); Halmahera, Sofifi, 00°45.47'N, 127°35.90'E, 1 specimen 37/20 mm [5067], st 205, *Sonneratia* mangrove (UMIZ 00035); Timor, Oesapa, 10°08.73'S, 123°38.10'E, 1 specimen 44/26 mm [5898], st 250, sandy area with *Sonneratia* and *Avicennia* (UMIZ 00072). **Australia**, Queensland, Cairns, Yule Point, 16°34.23'S, 145°30.58'E, 2 specimens 43/23 mm [2554] and 16/10 mm [2555], st 100, sand flat outside mangrove with dense *Rhizophora* (st100, MTQ). **Philippines**, Mindanao, [no information on the collecting date], 1 specimen 28/20 mm, paralectotype of *W. gracile*, [no information on the collector] (ZMB 103082b); Luzon, Batangas, Lian, 13°59.76'N, 120°37.43'E, 1 specimen 52/34 mm [3159], st 181, sandy, open *Avicennia* forest (PNM 041209); Bohol, Guindulman, 09°44.06'N, 124°27.63'E, 1 specimen 31/25 mm [3631], st 197, rocks, coral rubble, and sand near a few *Avicennia* trees (PNM 041210); Bohol, Loay, 09°36.23'N, 123°59.72'E, 1 specimen 35/20 mm [3637], st 198, mostly sand, and a few *Avicennia* (PNM 041211); Bohol, Maribojoc, 09°44.28'N, 123°49.39'E, 2 specimens 36/17 mm [3409] and 18/13 mm [3410], st 202, coral rubble with sand and algae, near *Sonneratia* (PNM 041212).

##### Distribution.

Australia: Queensland (type locality). Indonesia: Ambon, Bali, Halmahera, Kei Islands, Lombok, Seram, Sulawesi, and Timor. Philippines: Bohol and Luzon. All records, except the type locality, are new.

##### Habitat

(Fig. 19, Table 3). *Wallaconchis buetschlii* is found on firm mud or coarse sand in mangroves (i.e., mud which is not saturated in water) along with *Wallaconchis uncinus*. It also lives on fine, sandy mud along with *W. gracile*.

##### Diagnosis

(Table 5). The slugs that are part of *Wallaconchis buetschlii* are the largest of the genus (the largest live animal measured 60 mm). However, species cannot reliably be distinguished based on individual length. Red individuals cannot be distinguished from several species (usually *W. nangkauriense* and *W. ater*), and grey or brown individuals cannot be distinguished from other species. Dorsal papillae with yellow tips can be prominent in *W. buetschlii*, but also can be visible in other *Wallaconchis* species (particularly in *W. melanesiensis*). In addition, the dorsal papillae with yellow tips found on the notum of *W. buetschlii* are nearly identical to those on the notum of several species in other genera. The color of the hyponotum is helpful to distinguish *W. buetschlii* (grey or yellow-grey hyponotum) from species in other genera (orange hyponotum) that live in the same habitat but is not fully reliable and should only be used with caution (i.e., the orange hyponotum in other onchidiids is occasionally a dull orange-grey similar to the yellow-grey hyponotum of *W. buetschlii*). As a result, *W. buetschlii* cannot be reliably distinguished externally from other onchidiid species.

Internally, the penis is diagnostic of *W. buetschlii*: it is not protected by a penial sheath or a vestibule and bears internal longitudinal ridges. The oviduct attached to the posterior wall of the visceral cavity is peculiar but it also occurs in *W. gracile*. However, the penis of *W. gracile* is protected by a penial sheath and extends into the vestibule.

##### Color and morphology of live animals

(Fig. 20). The dorsal coloration is variable. It is usually brown or grey, but occasionally brown with red or yellow patches, or even completely red. In some individuals, many small yellow dots are present on the dorsal notum. The color of the ocular tentacles also varies, and may be yellow-orange, brown, or reddish brown. The hyponotum color can be grey, light grey, or yellow-grey. The foot is also grey or yellow-grey. Crawling slugs tend to be elongated, but they contract when disturbed.

##### External morphology.

There are many papillae on the dorsal surface, which are usually bright yellow in color, or occasionally brown. Between six and 14 papillae bear eyes, with three or four eyes per papilla. There is a large, retractable papilla with eyes in the center of the dorsal notum, and it is often raised several millimeters above the dorsal surface.

##### Digestive system

(Fig. 21, Table 4). Examples of radular formulae are presented in Table 4. The length of the rachidian teeth is approximately 20 µm, significantly smaller than that of the lateral teeth. The length of the hook of the lateral teeth gradually increases, from 40 to 75 µm, from the inner to the outer teeth, excluding the innermost and outermost lateral teeth which are significantly smaller. The intestinal loops are of type I.

##### Reproductive system

(Fig. 22A). The distal region of the oviduct forms a loop, approximately 1/2 to 3/4 of a circle, attached by fibers of tissue to the inner wall of the visceral cavity (Fig. 22A). The oviduct is narrow and approximately of the same width as the deferent duct. The spermatheca is apple-shaped and joins the middle part of the oviduct through a short duct.

##### Copulatory apparatus

(Figs 22B, C, 23). The anterior, male copulatory apparatus exclusively consists of the deferent duct, the penis, and the retractor muscle of the penis. The penis, distal to the deferent duct, is a long and smooth tube, which can evaginate like a glove. It is not protected by a distinct penial sheath and, strictly speaking, there is no vestibule either. The penis is narrow proximally, widens gradually, with three longitudinal, internal ridges distally (one ridge shown in Fig. 23). In small individuals, the penis is narrow for its entire length. The relative length of the penis compared to that of the retractor muscle varies greatly, from both being of similar length to the penis being five times longer than the retractor muscle. The retractor muscle inserts near the rectum at the posterior end of the visceral cavity. In mature specimens, the deferent duct is thicker than the penis and convoluted distally (Fig. 22C). In immature individuals, the deferent duct is thinner, and less convoluted (Fig. 22B).

##### Remarks.

Stantschinsky’s *Onchidium buetschlii* refers to a *Wallaconchis* species because of the unique combination of characters of the lectotype (intestine of type I, no penial accessory gland, no rectal gland). Stantschinsky’s original description of the penis (confirmed by our examination of the lectotype) matches exactly the penial anatomy of the species described here. The unique attachment of the oviduct to the posterior body wall was not described by Stantschinsky but is clearly present in the lectotype as well. Because the male parts of the two paralectotypes of *W. buetschlii* are missing, it cannot be confirmed that they are part of *W. buetschlii*. The anatomy of the posterior (female) reproductive parts of the paralectotypes matches that of the lectotype (with an oviduct attached to posterior body wall), indicating that they could be part of either *W. buetschlii* or *W. gracile*.

The two syntypes used by Stantschinsky to describe *Onchidium gracile* are part of two distinct species; therefore, one specimen is designated as the lectotype (see *Wallaconchis gracile*). The paralectotype of *O. gracile* is part of *W. buetschlii*. Both species share the unique attachment of the oviduct to the posterior body wall and are anatomically extremely similar. However, the penis (of the lectotype) of *W. gracile* is within a penial sheath, while the penis (of the lectotype) of *W. buetschlii* lacks a penial sheath. The penis of the paralectotype of *W. gracile* matches perfectly that of (the lectotype of) *W. buetschlii*. The written description of *O. gracile* (Stantschinsky 1907: 380–383) is confusing because it likely is a mix of both *W. buetschlii* (paralectotype) and *W. gracile* (lectotype). However, Stantschinsky’s (1907: pl. 13, fig. 34) illustration of *O. gracile* matches the copulatory apparatus of the paralectotype and is thus an illustration of *W. buetschlii*.

Hoffmann (1928) commented that *O. buetschlii* is similar to *Onchidium palaense*, but he did not list any new material for either species, and he does not seem to have examined the type material (he does not mention that he examined it). Semper’s description of the position of the male opening in *O. palaense* as “between the two very small tentacles, almost in the middle between these” (1880: 276) differs from all *Wallaconchis* species and indicates that *O. palaense* does not belong to *Wallaconchis*. The application of the name *O. palaense* will be discussed in the revision of another onchidiid genus.

Labbé (1934) identified two specimens from New Guinea as *Paraoncidium buetschlii*, but he also indicated they might be part of *O. palaense*, both of which were originally described with no rectal gland. The lack of rectal gland combined with traits described by Labbé (intestinal loops of type I, no accessory penial gland) indicate that the specimens he examined are part of a *Wallaconchis* species. However, Labbé’s brief description does not provide enough information to determine whether the specimens he examined are part of *W. buetschlii* or another *Wallaconchis* species without penial hooks. The fact that Labbé did not see any dorsal eyes was most likely due to preservation because dorsal eyes are present in all known *Wallaconchis* species. Finally, Bretnall (1919) summarized Stantschinsky’s original description of *W. buetschlii* but did not examine any new material.

#### Wallaconchis gracile

Taxon classificationAnimaliaSystellommatophoraOnchidiidae

(Stantschinsky, 1907) comb. n.

**Figure 24.**
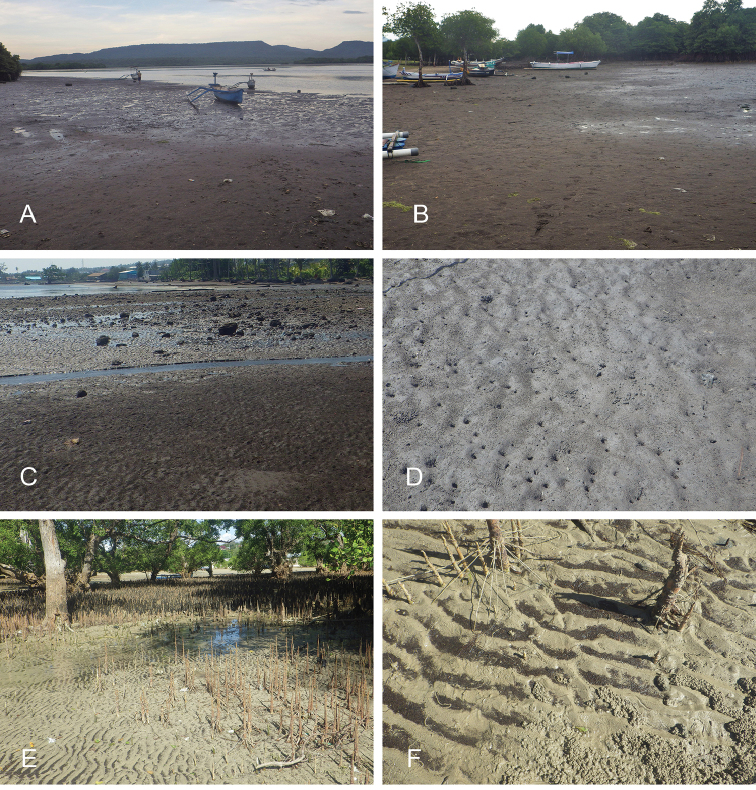
Habitats, *Wallaconchis gracile*, Indonesia. **A–B** Bali, Gilimanuk, muddy-sandy beach near a mangrove (st 155) **C–D** Ternate, sandy mudflat adjacent to a rocky beach (st 220) **E–F** Timor, sandy area of mangrove with ripples in the sand and near crab pellets (st254).

**Figure 25.**
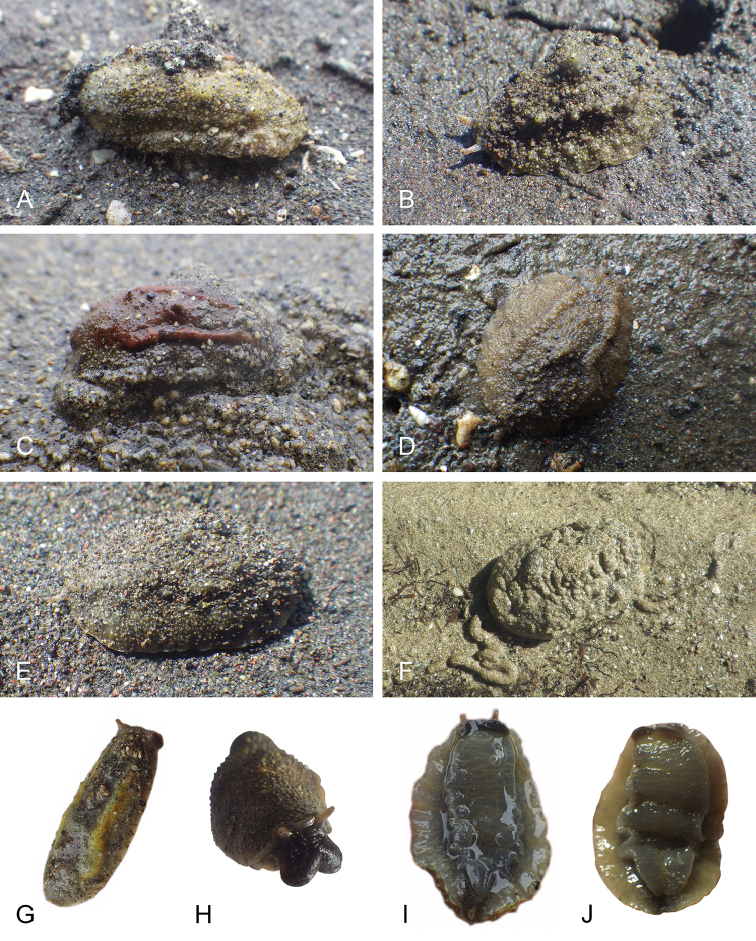
Live specimens, *Wallaconchis gracile*. **A** Dorsal view, 19 mm long [3107], Indonesia, Bali (UMIZ 00056) **B** Dorsal view, 15 mm long [5158], Indonesia, Halmahera (UMIZ 00057) **C** Dorsal view, 21 mm long [3648], Philippines, Bohol (PNM 041231) **D** Dorsal view, 19 mm long [2252], Indonesia, Sulawesi (UMIZ 00055) **E** Dorsal view, 15 mm long [5159], Indonesia, Halmahera (UMIZ 00057) **F** Dorsal view, 27 mm long [5932], Indonesia, Timor (UMIZ 00074) **G** Dorsal view, 25 mm long [3652], Philippines, Bohol (PNM 041231) **H** Frontal view, same as F **I** Ventral view, 25 mm long [3653], Philippines, Bohol (PNM 041231) **J** Ventral view, same as F.

**Figure 26.**
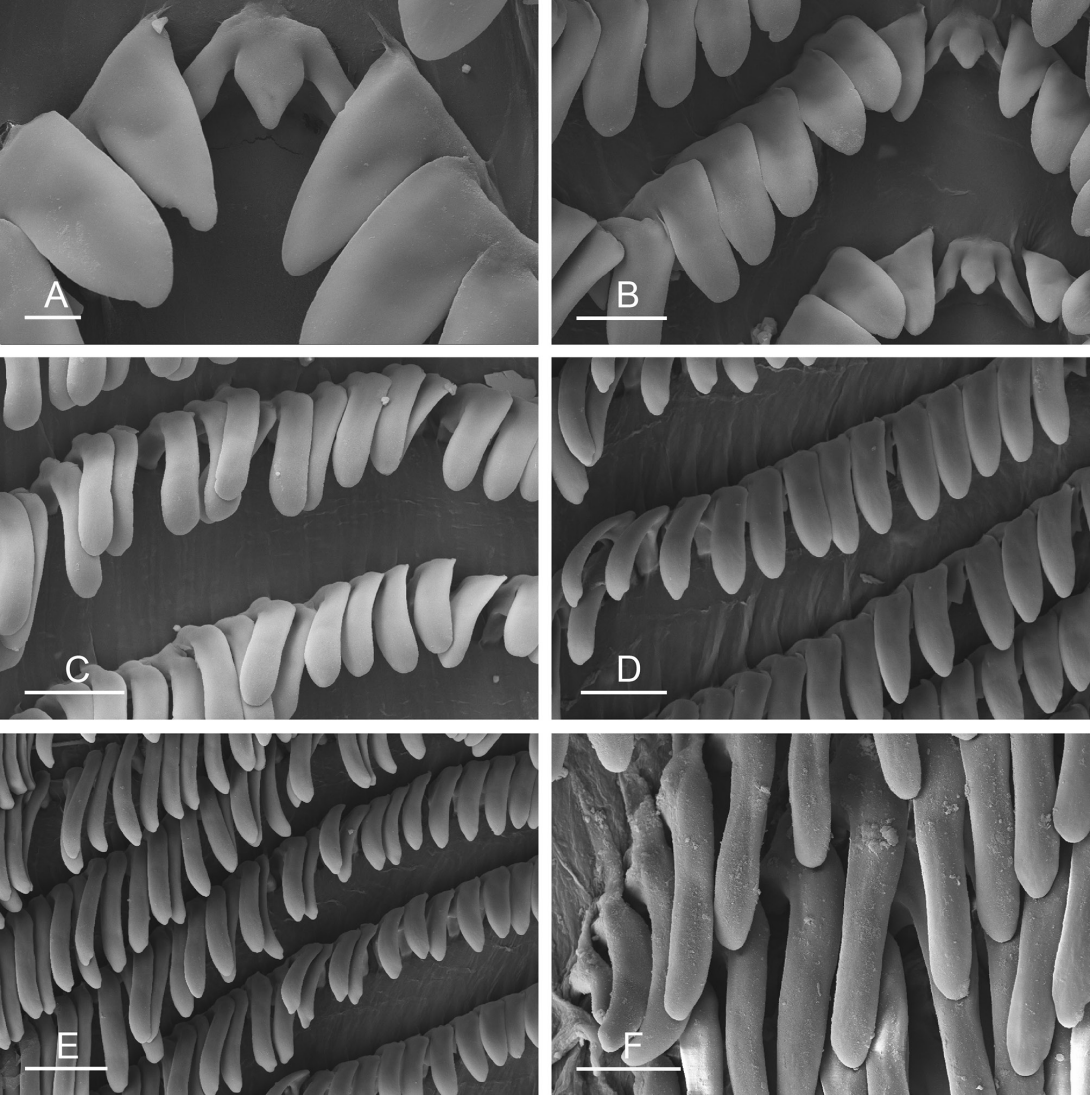
Radula, *Wallaconchis gracile*, Indonesia, Bali (**A–C**) [3106] (UMIZ 00056) (**D**) [spm #1] (UMIZ 00056). **A** Rachidian and innermost lateral teeth, scale bar 6 μm **B** Rachidian and innermost lateral teeth, scale bar 20 μm **C** Transition between inner lateral teeth and outer lateral teeth, scale bar 30 μm **D** Left lateral teeth, scale bar 30 μm **E** Left lateral teeth, gradually increasing in length towards the left, scale bar 40 μm **F** Outermost lateral teeth, scale bar 20 μm.

**Figure 27.**
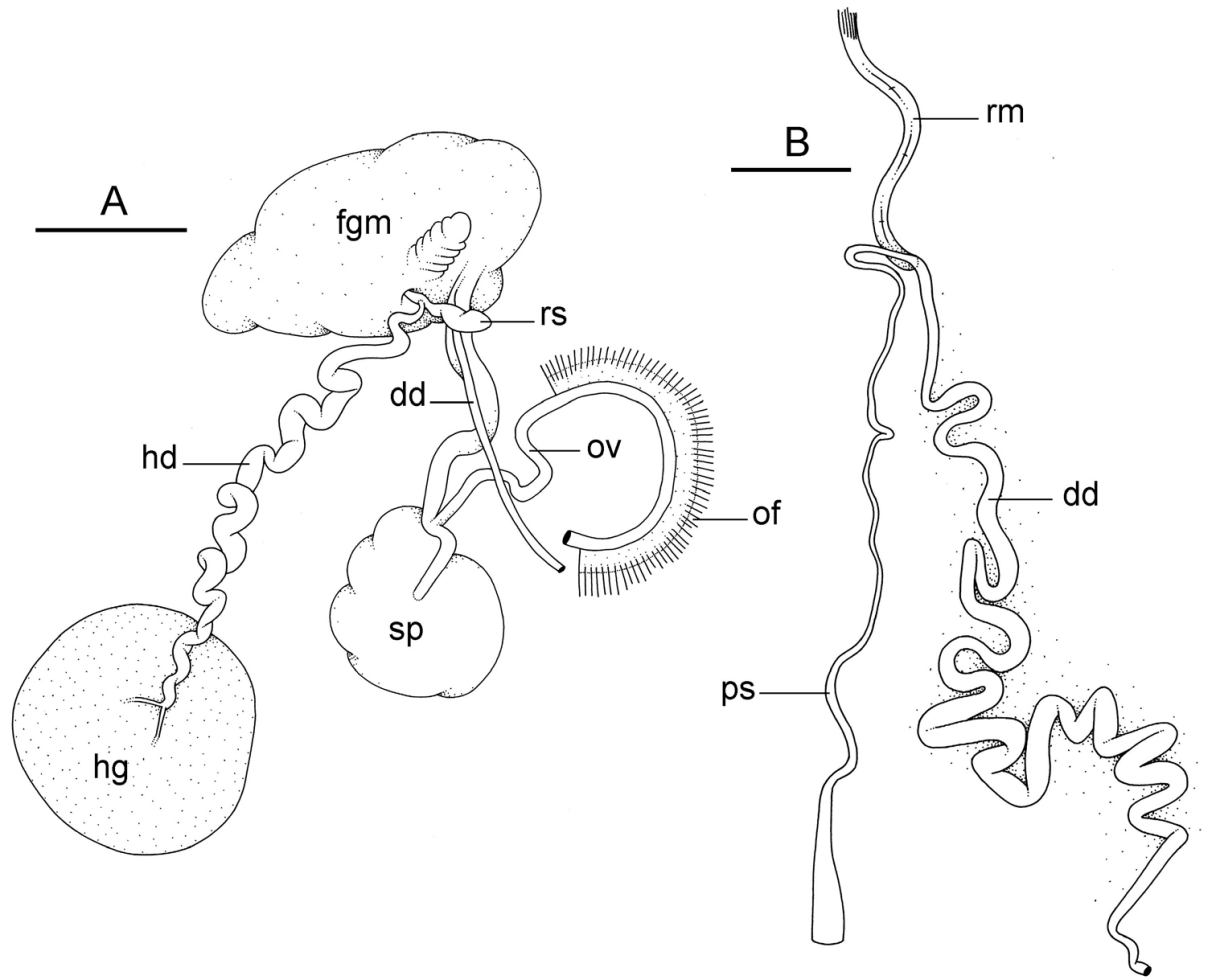
Reproductive system, *Wallaconchis gracile*, Indonesia. **A** Hermaphroditic (female), posterior parts, Sulawesi, scale bar 2 mm [2280] (UMIZ 00055) **B** Anterior, male copulatory parts, Bali, scale bar 2 mm [3106] (UMIZ 00056). Abbreviations: **dd** deferent duct **fgm** female gland mass **hd** hermaphroditic duct **hg** hermaphroditic gland **of** oviduct fibers attaching to the inner wall of the visceral cavity **ov** oviduct **ps** penial sheath **rm** retractor muscle **rs** receptaculum seminis **sp** spermatheca.

**Figure 28.**
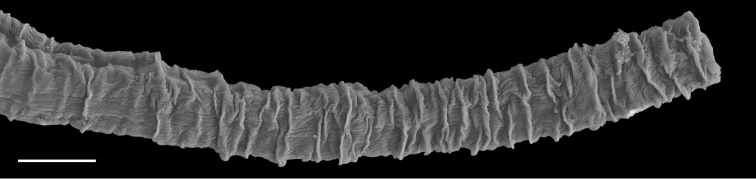
Penis, distal part free in the vestibule, *Wallaconchis gracile*, Indonesia, Bali, scale bar 40 μm [3106] (UMIZ 00056).

Figs 24 , 25 , 26 , 27 , 28

Onchidium gracile Stantschinsky, 1907: 380–383, pl. 12, figs 7–9, pl. 13, fig. 25 (as Oncidium gracile).

##### Type locality.

Mindanao, Philippines. No fresh material was collected from the type locality. However, fresh material was collected from Bohol (Philippines), just north of Mindanao, and from Halmahera (Indonesia), just south of Mindanao.

##### Type material.

Lectotype, 25/20 mm, designated here (ZMB 103082a). The lectotype was opened previously but all internal organs remain. The other syntype (28/20 mm), which becomes a paralectotype (ZMB 103082b) was dissected by Stantschinsky, but all of the internal organs remain. The paralectotype of *W. gracile* is part of *W. buetschlii*, which is not an issue because it is no longer a name-bearing type (see the remarks on both *W. gracile* and *W. buetschlii*).

##### Additional material examined.

**Indonesia**, North Sulawesi, Wori, 01°36.06'N, 124°51.73'E, 4 specimens 20/13 mm [2280], 19/15 mm [2252], 9/5 mm [2276], and 8/5 mm [2277], st 90, old *Avicennia*, *Sonneratia*, and *Rhizophora* mangrove with rocks (UMIZ 00055); Bali, Gilimanuk, 08°10.26'S, 114°26.61'E, 3 specimens 22/22 mm [#1], 19/9 mm [3107] and 17/10 mm [3106], st 155, muddy-sandy beach near a mangrove (UMIZ 00056); North Maluku, Ternate, 00°45.18'N, 127°20.17'E, 2 specimens 15/10 mm [5158] and 15/9 mm [5159], st 220, sandy beach behind a rock wall (UMIZ 00057); Timor, Oesapa, 10°08.69'S, 123°38.21'E, 1 specimen 27/18 mm [5932], st 254, mangrove with fine sand (UMIZ 00074). **Philippines**, Bohol, Loay, 09°36.23'N, 123°59.72'E, 1 specimen 10/5 mm [3633], st 198, mostly sand, and a few *Avicennia* (PNM 041230); Bohol, Loon, 09°49.91'N, 123°48.33'E, 3 specimens 25/10 mm [3653], 25/10 mm [3652] and 21/11 mm [3648], st 201, fine sand in front of *Rhizophora* forest (PNM 041231).

##### Distribution.

Indonesia: Bali, Halmahera, Sulawesi, and Timor. Philippines: Bohol and Mindanao (type locality). All records are new except for the type locality.

##### Habitat

(Fig. 24, Table 3). *Wallaconchis gracile* is found on muddy sand at the margin of mangroves, by the sea (just a few meters away from it). This type of muddy sand is not as saturated with water as a mudflat, nor as firm as a sandy beach. The slugs are sensitive to changes in the pressure on the muddy sand and usually dig in when approached. *Wallaconchis gracile* is specific to its habitat, and slugs are found only where patches of muddy sand are found.

##### Diagnosis

(Table 5). *Wallaconchis gracile* cannot be distinguished externally from other *Wallaconchis* species. Grey or brown individuals cannot be distinguished from any other *Wallaconchis* species. Even patches of color (i.e., red, yellow- orange, or brown-black) do not help distinguish it from similar species (*W. uncinus*, *W. nangkauriense*, *W. buetschlii*, *W. ater*, and *W. comendadori*).

Internally, the attachment of the oviduct to the body wall distinguishes *W. gracile* from the species with a similar penial anatomy (i.e., *W. sinanui*, *W. comendadori*, and *W. melanesiensis*), but is shared with *W. buetschlii*. The penial sheath enclosing the penis of *W. gracile* distinguishes it from *W. buetschlii*. Also, the retractor muscle is thicker in *W. gracile* than in *W. buetschlii*.

##### Color and morphology of live animals

(Fig. 25). The dorsal color is usually brown, greyish brown, or dark brown, occasionally with an orange or yellow hue. The color may be homogenous or with brown patterns. Longitudinal stripes or a patch of color (red, yellow-orange, or brown-black) are sometimes present. The hyponotum is light yellow-orange. The foot is light grey.

##### External morphology.

Between six and eight dorsal papillae bear eyes, with three or four per papilla. There is a retractable papilla with eyes in the center of the dorsal notum, which may be slightly raised above the dorsal surface.

##### Digestive system

(Fig. 26, Table 4). Examples of radular formulae are presented in Table 4. The length of the rachidian teeth is approximately 15 µm, significantly smaller than that of the lateral teeth. The length of the hook of the lateral teeth gradually increases (from 35 to 45 µm) from the inner teeth to the outer teeth, excluding the innermost and outermost lateral teeth which are significantly smaller. The intestinal loops are of type I.

##### Reproductive system

(Fig. 27A). The distal region of the oviduct forms a loop (approximately 1/2 to 3/4 of a circle) attached by fibers of tissue to the inner wall of the visceral cavity. The oviduct is narrow and approximately of the same width as the deferent duct. The surface of the spermatheca is marked by several lobes. A short duct connects the spermatheca to the middle part of the oviduct.

##### Copulatory apparatus

(Figs 27B, 28). The distal end of the penis lies free within the proximal part of a long, narrow vestibule, and the proximal end of the penis is protected within the penial sheath. The length of the penis varies between individuals, and ranges from approximately 1 – 3 mm. The penis is elongated, narrow, and does not bear hooks (Fig. 28). The narrow penial sheath measures approximately 6 – 8 mm long. Proximally, the vestibule is slightly wider than the penial sheath and gradually widens distally (Fig. 27B). The short retractor muscle is approximately 1.5 – 4 mm long and inserts at the posterior end of the visceral cavity near the rectum. In mature specimens, the deferent duct is convoluted and thicker than the penis.

##### Remarks.

*Onchidium gracile* refers to a *Wallaconchis* species due to a unique combination of characters (intestinal loops of type I, no accessory penial gland, and no rectal gland). Stantschinsky’s original description was based on two syntypes which are actually part of two species, hence the necessity of designating a lectotype. The anatomy of one of the former syntypes is in agreement with the species described here, so it is designated as the lectotype to clarify the application of the name *W. gracile*. The other former syntype, now a paralectotype without a name-bearing function, is anatomically indistinguishable from *W. buetschlii*. This nomenclatural decision helps avoid having to create a new species name for the species described here. *Wallaconchis gracile* and *W. buetschlii* are similar anatomically (Table 4) and it is understandable that Stantschinsky confused them. Stantschinsky’s original description and illustration (pl. 13, fig. 34) of the male parts of *W. gracile* were based on the paralectotype, and therefore correspond to *W. buetschlii*.

Stantschinsky (1907: 381, our translation from German) described *Onchidium gracile* as “very similar in appearance to *O. fungiforme*” and bearing “much resemblance with regard to internal structure.” Stantschinsky distinguished *O. gracile* from *O. fungiforme* externally by the mantle sculpture and the coloration, and internally by the anatomy of the buccal mass and the radular teeth. However, in our experience, all those characters are not informative for distinguishing species in *Wallaconchis* (or in any other onchidiid genus for that matter). The holotype (24/19 mm), by monotypy, of *O. fungiforme* (SMF 333604/1), was previously dissected by Stantschinsky. Although the digestive system and the female reproductive system are still present, the penial complex is missing. *Onchidium fungiforme* needs to be transferred to *Wallaconchis* because the holotype shares its unique combination of aforementioned characters given above (see diagnostic features of the genus *Wallaconchis*, p. 22). However, the name *W. fungiforme* is regarded here as a *nomen dubium* because its exact application is unclear. The female reproductive parts of the holotype of *W. fungiforme* bear the distinctive attachment of the oviduct to the body wall only known in *W. gracile* and *W. buetschlii*. Of these two species, Stantschinsky’s (1907: 380) description of the penis as “two sharply delineated portions, of which the longer posterior part passes, as a very thin tube, into a gradual thickening” distinguishes it from *W. buetschlii* and is only consistent with *W. gracile*. However, a species matching the anatomy of *W. fungiforme* was not found during extensive sampling in Queensland (29 stations) nor in the MNHN collections from Papua New Guinea. Therefore, it cannot be evaluated whether the male parts of *W. fungiforme* differ from those of *W. gracile* or not. Given the large distance (more than 2,500 kilometers) between our samples from the Coral Triangle and the type locality of Queensland, it is unclear whether the names *W. fungiforme* and *W. gracile* could apply to a single widely distributed species, or if *W. fungiforme* is distinct and endemic to Australia. Moreover, it is unclear where the holotype of *W. fungiforme* was collected exactly since its type locality is simply stated as “Queensland”.

Hoffmann (1928: 82) regarded *O. fungiforme* as a synonym of *Onchidium ovale*. However, *O. ovale* is regarded here as a *nomen dubium* because its type locality is also unknown. The holotype (22/14 mm) of *O. ovale* (ZMB 39045) was dissected by Semper. Its internal organs remain, with the exception of the male parts. *Onchidium ovale* clearly refers to a *Wallaconchis* species because intestinal loops of type I and a male opening below the right eye tentacle can still be observed in the holotype, and because Semper clearly mentioned the lack of penial accessory gland. The fact that the male parts of the holotype are missing and that they were never illustrated by Semper is yet another reason to regard *O. ovale* as a *nomen dubium*.

Hoffmann (1928: 82) regarded *O. gracile* as a synonym of *Onchidium palaense*. However, the position of the male opening in Semper’s description of *O. palaense* (between the ocular tentacles) indicates that *O. palaense* does not refer to a *Wallaconchis* species (see remarks on *W. buetschlii*).

#### Wallaconchis nangkauriense

Taxon classificationAnimaliaSystellommatophoraOnchidiidae

(Plate, 1893) comb. n.

**Figure 29.**
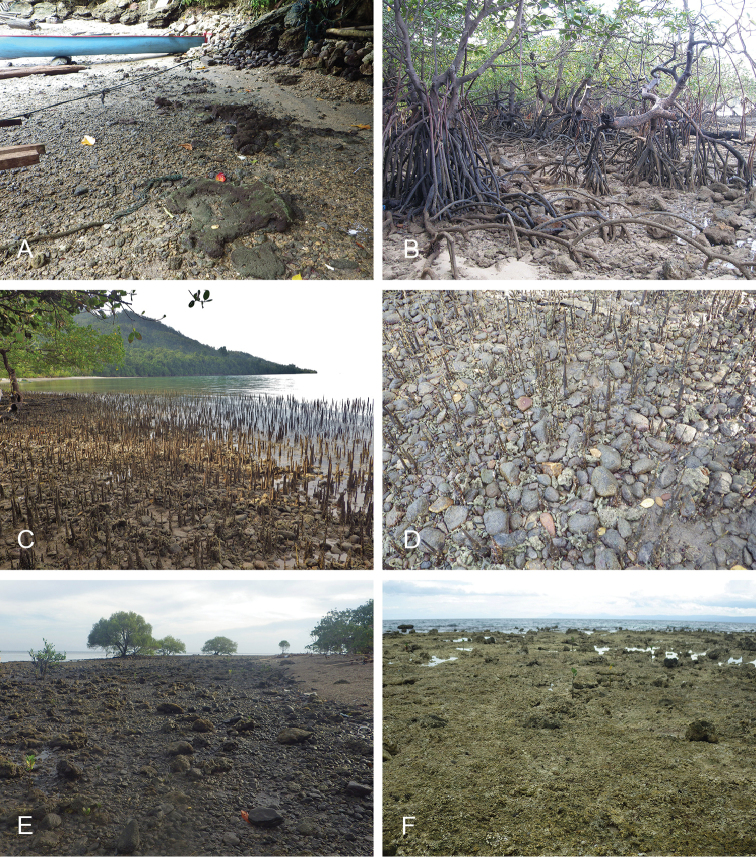
Habitats, *Wallaconchis nangkauriense*. **A** Indonesia, Sulawesi, sand, rocks, pieces of wood behind narrow coastal mangrove (st 85) **B** India, Andaman Islands, Burman Nala, slugs were on the stilt roots (st 53) **C** Indonesia, Ambon, Haruku Island, narrow mangrove of *Sonneratia* trees on rocky beach (st 127) **D** Close up (same locality as C) **E** Indonesia, Bali, Pemuteran, Labuhan Lalang Harbor, coral rubble, rocks and a few *Avicennia* (st 157) **F** Philippines, Bohol, Maribojoc, coral rubble of uplifted coral reef (st 191).

**Figure 30.**
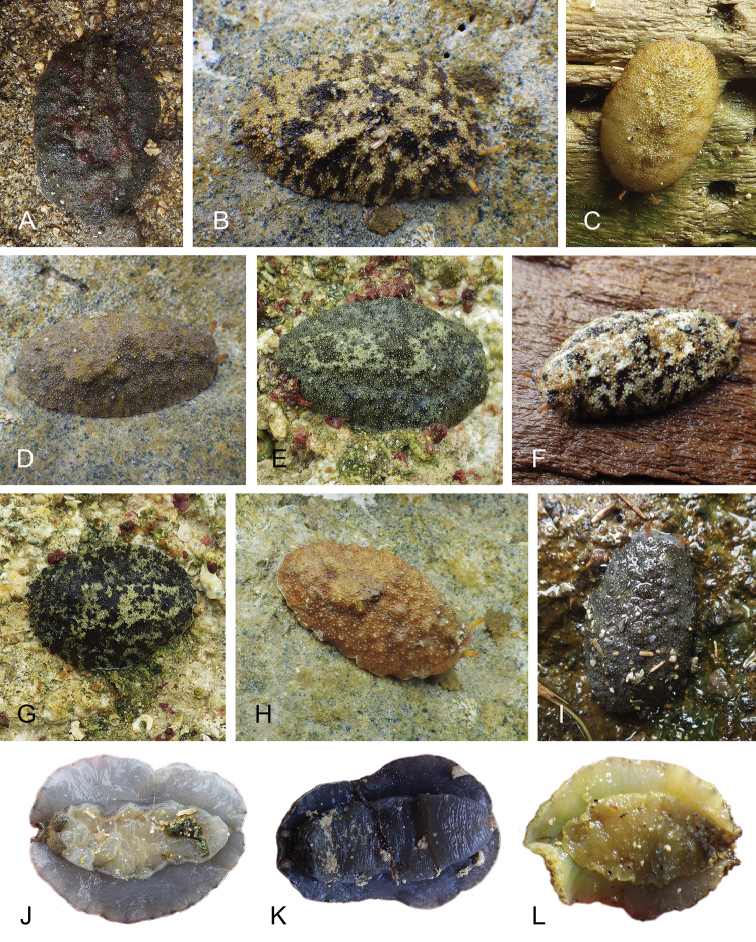
Live specimens, *Wallaconchis nangkauriense*. **A** Dorsal view, 28 mm long [3162], Philippines, Luzon (PNM 041204) **B** Dorsal view, 23 mm long [1786], Indonesia, Sumatra (UMIZ 00012) **C** Dorsal view, 18 mm long [2156], Indonesia, Sulawesi (UMIZ 00013) **D** Dorsal view, 23 mm long [1788], Indonesia, Sumatra (UMIZ 00012) **E** Dorsal view, 34 mm long [3269], Philippines, Bohol (PNM 041206) **F** Dorsal view, 24 mm long [2161], Indonesia, Sulawesi (UMIZ 00013) **G** Dorsal view, 32 mm long [3268], Philippines, Bohol (PNM 041206) **H** Dorsal view, 28 mm long [1787], Indonesia, Sumatra (UMIZ 00012) **I** Dorsal view, 24 mm long [3217], Philippines, Luzon (PNM 041205) **J** Ventral view, same as G **K** Ventral view, 28 mm long [3427], Philippines, Bohol (PNM 041208) **L** Ventral view, 22 mm long [2731], Indonesia, Ambon (UMIZ 00017).

**Figure 31.**
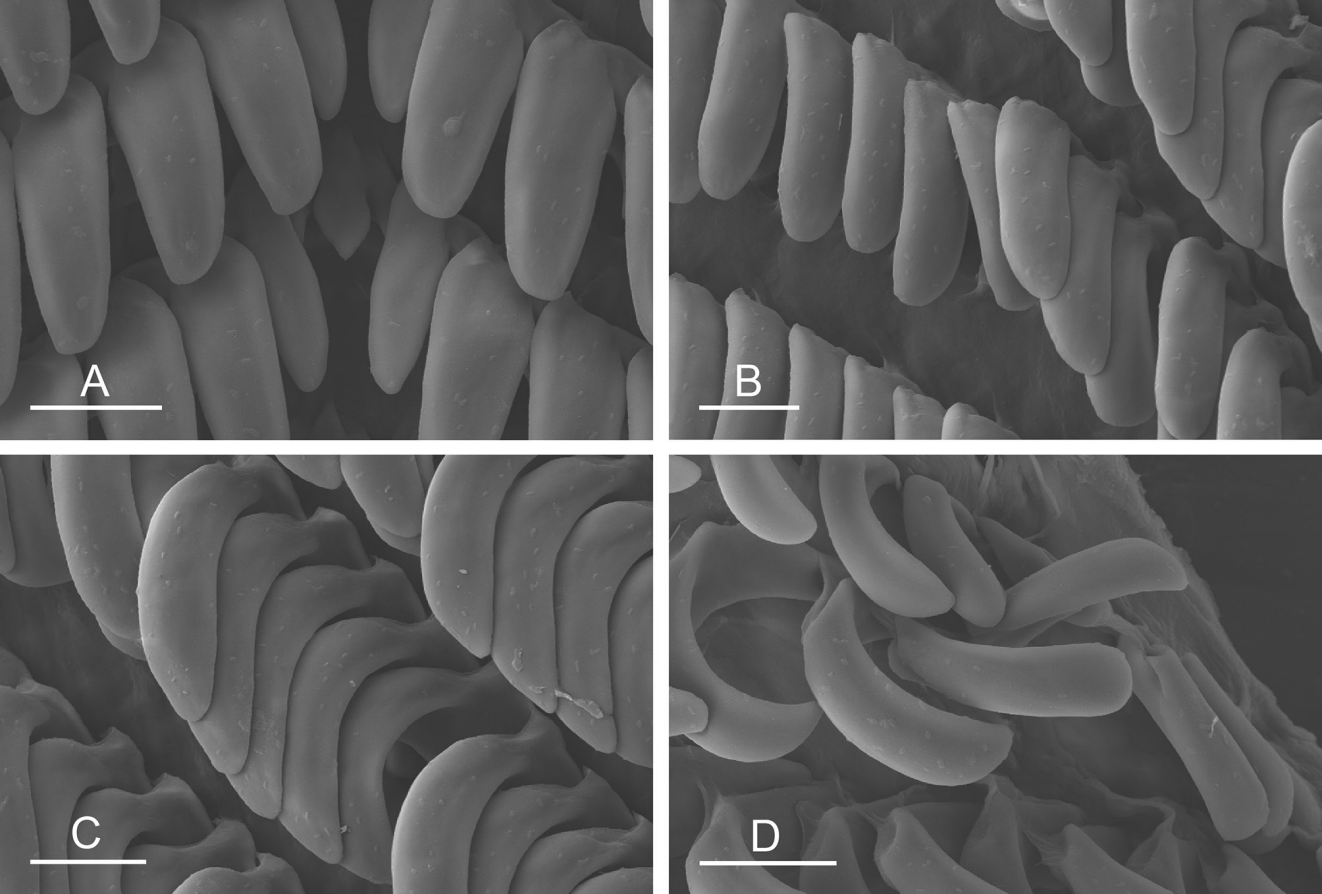
Radula, *Wallaconchis nangkauriense*, Indonesia, Bali [3129] (UMIZ 00020). **A** Rachidian and innermost lateral teeth, scale bar 20 μm **B** Innermost lateral teeth, scale bar 20 μm **C** Outer lateral teeth, scale bar 20 μm **D** Outermost lateral teeth, scale bar 20 μm.

**Figure 32.**
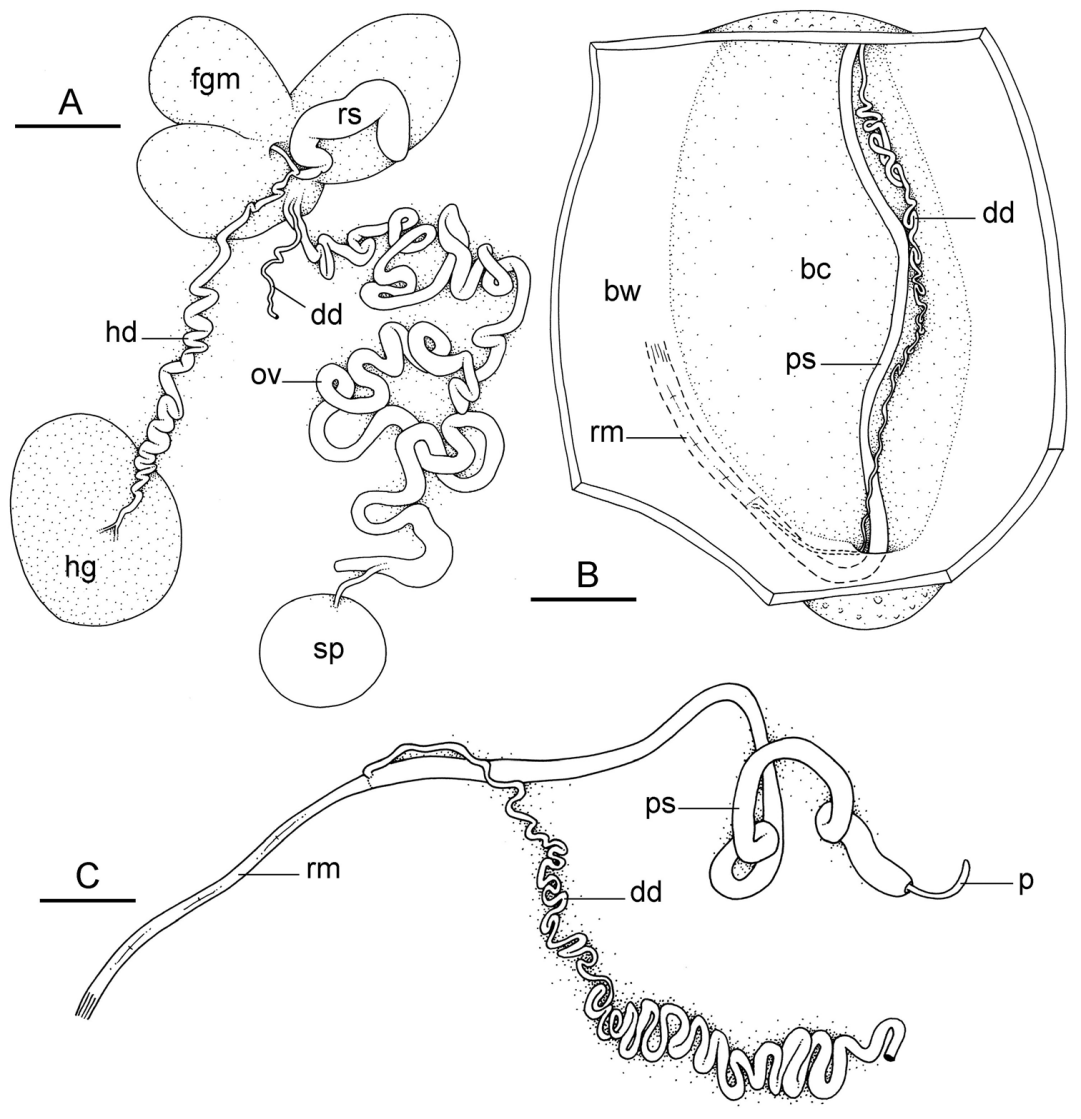
Reproductive system, *Wallaconchis nangkauriense*. **A** Hermaphroditic (female), posterior parts, Philippines, Bohol, scale bar 4 mm [3276] (PNM 041206) **B** Anterior, male copulatory parts, Indonesia, Sulawesi, scale bar 5 mm [2192] (UMIZ 00014) **C** Anterior, male copulatory parts (vestibule removed around the penis), Indonesia, Sumatra, scale bar 2 mm [1788] (UMIZ 00012). Abbreviations: **bc** body cavity **bw** lateral left body wall **dd** deferent duct **fgm** female gland mass **hd** hermaphroditic duct **hg** hermaphroditic gland **ov** oviduct **p** penis **ps** penial sheath **rm** retractor muscle **rs** receptaculum seminis **sp** spermatheca.

**Figure 33.**
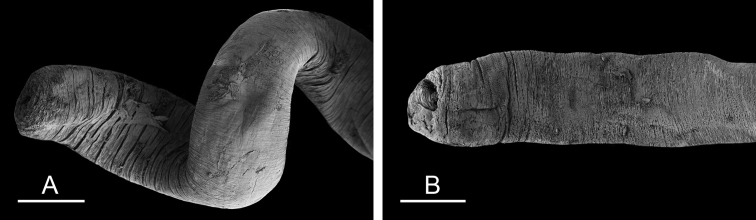
Penis, *Wallaconchis nangkauriense*, Philippines. **A** Luzon, scale bar 200 μm [3208] (PNM 041205) **B** Bohol, scale bar 200 μm [3268] (PNM 041206).

Figs 29 , 30 , 31 , 32 , 33

Onchidium nangkauriense Plate, 1893: 170–171, pl. 12, figs 84, 93–95 (as Oncidium nangkauriense).
Paraoncidium nangkauriense : Labbé 1934: 231.

##### Type locality.

Nangkauri, eine Insel der Nicobaren [Nancowry, Nicobar Islands, Indian Ocean]. No fresh material was collected from the type locality (access to the Nicobar Islands is extremely restricted). However, fresh material was collected from the Andaman Islands, just north of the Nicobar Islands (see below, additional material).

##### Type material.

Lectotype, 19/14 mm, designated here (ZMB 45659a). The lectotype was dissected prior to the present study but is well preserved, with all organs remaining except for the penis. The lectotype is designated because it still displays the diagnostic characters of the species (a retractor muscle inserted within the left side of the body wall). The paralectotype (22/15 mm) also was dissected prior to the present study (ZMB 45659b). A piece of the notum is missing, as well as the digestive glands, and a part of the female reproductive system.

##### Additional material examined.

**India**, Andaman Islands, South Andaman, Burman Nala, 11°33.23'N, 92°44.00'E, 2 specimens 25/12 mm [1074] and 24/10 mm [1075], st 53, rocky shore with a patch of *Rhizophora*, sand and coral rubble, but no mud. (BNHS 50). **Indonesia**, Sumatra, Lampung, Penegahan, 05°40.40'S, 105°33.76'E, 4 specimens 42/25 mm, 28/15 mm [1787], 23/15 mm [1788], and 23/15 mm [1786], st 78, coral rubble on beach exposed to estuary (UMIZ 00012); North Sulawesi, Bahoi, 01°43.36'N, 125°01.23'E, 2 specimens 25/14 mm [2161] and 18/13 mm [2156], st 85, sand and small rocks outside a mangrove (UMIZ 00013); North Sulawesi, Tamperong, 01°41.51'N, 125°00.80'E, 1 specimen 20/13 mm [2192], st 87, rocks behind mangrove of small *Rhizophora* trees (UMIZ 00014); North Sulawesi, Wori, 01°36.06'N, 124°51.73'E, 1 specimen 30/20 mm [2257], st 90, old mangrove forest with *Avicennia*, *Sonneratia*, and *Rhizophora*, with rocks (UMIZ 00016); Ambon, Haruku Island, 03°36.52'S, 128°25.07'E, 1 specimen 22/14 mm [2731], st 127, rocky *Sonneratia* mangrove with coral rubble (UMIZ 00017); Lombok, Seriwe Bay, 08°54.55'S, 116°22.22'E, 1 specimen 39/28 mm [2972], st 148, rocks with *Avicennia* trees (UMIZ 00018); Bali, Pemuteran, Labuhan Lalang Harbor, 08°08.61'S, 114°32.33'E, 2 specimens 20/12 mm [3136] and 18/12 mm [3129], st 157, coral rubble, rocks and a few *Avicennia* (UMIZ 00020). **Philippines**, Luzon, Batangas, Lian, 13°59.76'N, 120°37.43'E, 1 specimen 28/17 mm [3162], st 181, sandy, open *Avicennia* forest (PNM 041204); Luzon, Batangas, Calatagan, 13°55.32'N, 120°37.26'E, 2 specimens 30/17 mm [3208] and 24/14 mm [3217], st 183, rocks in *Avicennia* and *Rhizophora* mangrove (PNM 041205); Bohol, Maribojoc, 09°44.02'N, 123°47.45'E, 3 specimens 32/20 mm [3269], 32/23 mm [3268], and 28/20 mm [3276], st 191, coral rubble with sand (PNM 041206); Bohol, Loay, 09°36.23'N, 123°59.72'E, 1 specimen 21/14 mm [5763], st 198, mostly sand, and a few *Avicennia* (PNM 041229); Bohol, Maribojoc, 09°44.02'N, 123°47.45'E, 2 specimens 28/16 mm [3396] and 18/12 mm [3401], st 200, coral rubble with sand at night (PNM 041207); Bohol, Maribojoc, 09°44.28'N, 123°49.39'E, 1 specimen 28/16 mm [3427], st 202, coral rubble with sand and algae, near *Sonneratia* (PNM 041208).

##### Distribution.

India: Nicobar Islands (type locality) and Andaman Islands. Indonesia: Ambon, Bali, Lombok, Sulawesi, and southeastern Sumatra. Philippines: Bohol and Luzon. All records are new except for the type locality.

##### Habitat

(Fig. 29, Table 3). In the Andaman Islands, *W. nangkauriense* is found in rocky mangroves, on the roots of *Rhizophora* covered with algae (Fig. 29B). Throughout the rest of its range, *W. nangkauriense* is found predominantly on coral rubble or small stones, which may be mixed with mangrove trees (Fig. 29C, –D), adjacent to a mangrove (Fig. 29A) or a few isolated mangrove trees (Fig. 29E), or completely without mangrove trees (Fig. 29F). *Wallaconchis nangkauriense* frequently occurs with *W. ater*.

##### Diagnosis

(Table 5). *Wallaconchis nangkauriense* cannot be distinguished from other *Wallaconchis* species based on external features. Brightly-colored individuals (e.g., red, yellow, and orange) cannot be distinguished from *W. ater* and *W. graniferum*. Grey or brown specimens cannot be distinguished from any other *Wallaconchis* species. Internally, however, the extremely long oviduct is a diagnostic feature of *W. nangkauriense*. The long retractor muscle of the penis inside the posterior body wall is also distinctive (although it is not present in all individuals).

##### Color and morphology of live animals

(Fig. 30). In sandy habitats, sand grains may be stuck to the dorsal notum. The dorsal coloration is highly variable, often a mottling of two or three colors. The most common colors are grey, brown, black, and yellow; combinations of red, green and orange also occur. The colors of the hyponotum and foot are variable. The hyponotum is usually light grey or cream, occasionally dark grey or white. The foot is either cream, light grey or dark grey. The color of the ocular tentacles varies between yellow-orange, light brown and reddish brown.

##### External morphology.

The number of dorsal papillae with eyes (three or four per papilla) is between five and 15, but, exceptionally, 25 papillae were observed in one specimen. There is a retractable papilla with eyes in the center of the dorsal notum, which may be slightly raised above the dorsal surface.

##### Digestive system

(Fig. 31, Table 4). Examples of radular formulae are presented in Table 4. The length of the rachidian teeth is approximately 20 µm, significantly smaller than that of the lateral teeth. The length of the hook of the lateral teeth gradually increases, from 40 to 65 µm, along the half row from the inner teeth to outer teeth (excluding the innermost and outermost lateral teeth, which are significantly smaller). The intestinal loops are of type I.

##### Reproductive system

(Fig. 32A). Posteriorly, the oviduct is extremely long, convoluted, and slightly narrow. The spermatheca is spherical and joins the distal region of the oviduct through a thin, short duct.

##### Copulatory apparatus

(Figs 32B, C, 33). The distal end of the penis lies free within a long, narrow vestibule, and its proximal end is protected within the penial sheath (in Fig. 32C, distal part of penial sheath removed). The length of the penis varies between individuals and geographic localities and ranges from approximately 9–22 mm. In specimens from the Philippines, the penis tends to be longer and frequently folds back upon itself within the vestibule. The penis is elongated, narrow, and smooth with no hooks (Fig. 33). The length of the penial sheath varies from a few millimeters (in small individuals) to longer than the body length. In addition, the proximal end of the penial sheath may be partially hidden within the left posterior body wall (Fig. 32B). The deferent duct is highly convoluted with many loops; however, in immature specimens, the deferent duct is significantly less convoluted. The retractor muscle enters the body wall at the posterior end of the visceral cavity near the rectum and lies (partially or completely) hidden inside the body wall. The insertion of the retractor muscle varies. In large specimens, the retractor muscle may extend anteriorly inside the left side of the body wall and insert up to half way up the body; in smaller specimens, it inserts within the posterior region of the left body wall. Exceptionally, in large specimens from southeastern Sumatra and the Andaman Islands, the retractor muscle inserts on the posterior wall of the visceral cavity, near the rectum, and not within the body wall. Finally, in small specimens from the Andaman Islands, the retractor muscle inserts on the body wall, near the heart, on the right side of the visceral cavity.

##### Remarks.

*Onchidium nangkauriense* belongs to *Wallaconchis* because it shares its unique combination of characters (intestinal loops of type I, no rectal gland, no dorsal gills, no penial accessory gland). Also, there is strong evidence that the name *W. nangkauriense* applies to the species being described here. Indeed, Plate (1893: pl. 12, fig. 84) illustrated an extremely long oviduct, a character which matches exactly the species described here and has not been found in any other onchidiid species, as well as a long retractor muscle entering the body wall at the posterior end of the body cavity and extending anteriorly inside the left side of body wall, a peculiar character only known in this onchidiid species.

In the same publication, Plate also described *Onchidium simrothi* from the same type locality as *O. nangkauriense* (Nangkauri, Nicobar Islands, India). *Onchidium simrothi* is transferred here to *Wallaconchis* but is regarded as a *nomen dubium* because important characters are not mentioned in the original description and could not be observed in the type material. The two syntypes of *O. simrothi* are small (10/6 and 10/5 mm) and in very poor condition (they previously dried, and most of the organs are missing, with the exception of parts of the female reproductive system and of the digestive system). Plate does not indicate whether the rectal gland is present or absent, but the intestine of type I and the absence of an accessory penial gland indicate that the two syntypes of *O. simrothi* belong to a *Wallaconchis* species or a *Platevindex* species. However, the male opening of *O. simrothi* (below the right tentacle) and its central dorsal tubercle with multiple eyes are inconsistent with *Platevindex*. We therefore consider that *O. simrothi* belongs to *Wallaconchis*. Given that both *W. simrothi* and *W. nangkauriense* were described from the Nicobar Islands, that the Nicobar Islands are within the geographic range of only one *Wallaconchis* species (*W. nangkauriense*) and are very far from the limits of distribution of all other *Wallaconchis* species, it is most likely that both names are synonyms. According to Plate, the difference between *O. simrothi* and *O. nangkauriense* is the length of their penis (4 mm long and 55 mm, respectively). However, our observations show that the small specimens (< 20 mm long) of *W. nangkauriense* from the Andaman Islands (which are very close to the Nicobar Islands) have a smaller penis with a retractor muscle inserted halfway down the body, which could explain the shorter penis described by Plate in the tiny (10 mm long) syntypes of *O. simrothi*. However, strictly speaking, neither Plate’s original description of *O. simrothi* nor the dried remains of its type material can be used to apply this name to any species. Therefore, *O. simrothi* is transferred to *Wallaconchis* but is regarded as a *nomen dubium*.

*Onchidium nangkauriense* was transferred by Labbé (1934) to his genus *Paraoncidium* (because of the lack of penial accessory gland in *O. nangkauriense*), but he did not comment on the species itself. Labbé (1934) re-described *O. simrothi* based on new material from New Guinea, which is far from the type locality (Nangkauri, Nicobar Islands). However, Labbé’s specimens were not part of a *Wallaconchis* species (and thus could not be part of *O. simrothi*) because their median male opening is incompatible with *Wallaconchis*. Considering the lack of information about the reproductive anatomy in Plate’s original description of *O. simrothi*, Labbé likely applied *O. simrothi* arbitrarily to his specimens from New Guinea, based mostly on the preserved color and the width of the foot in relation to the hyponotum, aspects which do not suffice to distinguish species. Hoffmann (1928) included both *O. nangkauriense* and *O. simrothi* in his revision but did not examine any new material or add further comment.

#### Wallaconchis ater

Taxon classificationAnimaliaSystellommatophoraOnchidiidae

(Lesson, 1830) comb. n.

Figs 34 , 35 , 36 , 37 , 38 , 39 , 40 , 41 , 42

Onchidium ater Lesson, 1830: 300; Bretnall 1919: 327; Hoffmann 1928: 84–85.
Onchidium keiense Hoffmann, 1926: 18–24, figs 1–5; Hoffmann 1928: 81(as Oncidium keiense). **syn. n.**
Scaphis atra : Labbé 1934: fig. 30.
Paraoncidium keiense : Labbé 1934: 230.
Lessonina ferruginea : Labbé 1934: fig. 50 [non Onchidium ferrugineum Lesson, 1830].

##### Type locality

(*O. ater*). Havre de Doréry (for Dorey), à la Nouvelle Guinée [Manokwari, West Papua, Indonesia]. No fresh material was collected from the type locality. However, fresh material was collected from Halmahera, approximately 700 kilometers from the type locality in West Papua (see below, additional material).

**Type locality** (*O. keiense*). Toeal (Kei-Inseln) [Kei Islands, Moluccan Islands, Indonesia]. Fresh specimens were collected from the type locality (see below, additional material).

**Type material** (*O. ater*). Two syntypes: 30/17 and 28/15 mm (MNHN 22950). Both syntypes were previously dissected and some internal organs are missing. The posterior (female) reproductive parts remain in the smaller syntype and only the oviduct remains in the larger syntype. The male (anterior) parts are missing in both syntypes. The digestive system is present in both syntypes, but the intestinal loops are not preserved.

**Type material** (*O. keiense*). Lectotype, 32/21 mm, designated here (ZMUC). Thirty paralectotypes (35/24 to 11/8 mm) are well preserved (ZMUC), although several specimens were dissected prior to the present study and the male parts were previously removed (ZMUC). The lectotype was dissected for this study and all organs left in vials inside the jar. The species name on the label is *Onchidium mortenseni*. However, the rest of the information on the label (specimens collected by Mortensen from Toeal and studied by Hoffmann) is identical to the information in Hoffmann’s original description of *O. keiense* (the collector is not mentioned in the species description, but the title of the paper indicates the species are described based on collections from Mortensen’s expedition). The only other onchidiid species described by Hoffmann from the Kei Islands was *Onchidium verruculatum* Cuvier, 1830, which Hoffmann recognized thanks to the presence of gills on the posterior notum (*O. verruculatum* actually belongs to the genus *Peronia*). Gills are not visible on any of the syntypes labeled *O. mortenseni*, which strongly suggests that those specimens are the type material of *O. keiense* and that Hoffmann changed his mind for the specific name (i.e., he replaced *mortenseni* by *keiense*).

##### Additional material examined.

**Indonesia**, North Sulawesi, Bahoi, 01°43.36'N, 125°01.23'E, 5 specimens 37/15 mm [2170], 30/15 mm [2145], 26/16 mm [2164], 25/14 mm [2157] and 25/11 mm [2177], st 85, sand and small rocks outside a mangrove (UMIZ 00049); North Sulawesi, Bahoi, 01°43.36'N, 125°01.23'E, 3 specimens 30/18 mm [2221], 29/16 mm [2220] and 26/17 mm [2228], st 88, sand and small rocks outside a mangrove (UMIZ 00050); North Sulawesi, Wori, 01°36.06'N, 124°51.73'E, 1 specimen 33/22 mm [2283], st 90, old *Avicennia*, *Sonneratia*, *Rhizophora* mangrove (UMIZ 00038); North Sulawesi, Mantehage Island, 01°41.88'N, 124°46.74'E, 1 specimen 25/16 mm [2330], st 91, rocks behind a *Sonneratia* and *Rhizophora* mangrove (UMIZ 00039); Ambon, Haruku Island, 03°36.52'S, 128°25.07'E, 1 specimen 9/8 mm [2727], st 127, rocky *Sonneratia* mangrove with coral rubble (UMIZ 00040); Maluku, Kei Islands, Tual City, Fiditan, 5°35.96'S, 132°45.11'E, 1 specimen 17/9 mm [2939], st 144, rocks behind muddy mangrove of *Rhizophora* trees (UMIZ 00041); Lombok, Seriwe Bay, 08°51.70'S, 116°32.87'E, 1 specimen 18/10 mm [2966], st 147, small beach of coral rubble and rocks by bay (UMIZ 00042); Lombok, Seriwe Bay, 08°54.55'S, 116°22.22'E, 2 specimens 43/20 mm [2970] and 35/24 mm [2974], st 148, rocks behind mangrove with *Avicennia* trees (UMIZ 00043); Lombok, Don Don, 08°54.54'S, 116°21.50'E, 2 specimens 35/19 mm [2986] and 25/13 mm [2978] st 149, old *Avicennia* forest with coral rubble (UMIZ 00051); Bali, Gilimanuk, 08°10.26'S, 114°26.61'E, 1 specimen 18/14 mm [3591], st 155, large rocks near a patch of *Rhizophora* (UMIZ 00052); Bali, Pemuteran, Labuhan Lalang Harbor, 08°08.61'S, 114°32.33'E, 3 specimens 22/12 mm [3137], 20/11 mm [3132], and 14/8 mm [3130], st 157, coral rubble, rocks and a few *Avicennia* (UMIZ 00053); North Maluku, Ternate, Bastiong, 00°46.41'N, 127°22.76'E, 1 specimen 38/17 mm [5057], st 203, muddy rocks near a mangrove (UMIZ 00046); Halmahera, Akelamo, 01°01.33'N, 127°39.09'E, 1 specimen 35/20 mm [5078], st 207, sandy-muddy beach at margin of mangrove (UMIZ 00047); Halmahera, Foli, 01°14.66'N, 128°10.61'E, 1 specimen 28/16 mm [5121], st 217, rocky shore near a beach (UMIZ 00048); Halmahera, Foli, 01°14.66'N, 128°10.61'E, 1 specimen 25/10 mm [5125], st 217, rocky shore near a beach (UMIZ 00054). **Philippines**, Luzon, Batangas, Calatagan, 13°55.32'N, 120°37.26'E, 3 specimens 30/16 mm [3215], 29/16 mm [3212] and 26/12 mm [3210], st 183, rocks in *Avicennia* and *Rhizophora* mangrove (PNM 041221); Bohol, Maribojoc, 09°44.02'N, E 123°47.45'E, 6 specimens 36/20 mm [3266], 34/21 mm [3271], 33/18 mm [3265], 33/22 mm [3272], 32/20 mm [3277], and 30/17 mm [3270], st 191, uplifted coral rubble with sand (PNM 041222); Bohol, Mabini, 09°51.59'N, 124°34.16'E, 2 specimens 21/12 mm [3373] and 14/8 mm [3370], st 196, open *Avicennia* and *Sonneratia* mangrove with sand, and coral rubble (PNM 041223); Bohol, Guindulman, 09°44.06'N, 124°27.63'E, 2 specimens 38/15 mm [3624] and 22/13 mm [3629], st 197, rocks and coral rubble near a few *Avicennia* trees (PNM 041216); Bohol, Loay, 09°36.23'N, 123°59.73'E, 1 specimen 33/19 mm [3634], st 198, mostly sand, and a few *Avicennia* (PNM 041217); Bohol, Maribojoc, 09°44.02'N, 123°47.45'E, 2 specimens 29/16 mm [3393] and 19/12 mm [3404], st 200, coral rubble with sand, at night (PNM 041218); Bohol, Maribojoc, 09°44.28'N, 123°49.39'E, 4 specimens 37/17 mm [3420], 33/23 mm [3406], 26/16 mm [3405] and 21/11 mm [3408], st 202, uplifted coral rubble with sand and algae, near *Sonneratia* trees (PNM 041224).

##### Distribution.

Indonesia: Bali, Edam (near Jakarta), Halmahera, Kei Islands (type locality of *O. keiense*), Lombok, Seram, Sulawesi, and West Papua (type locality of *O. ater*). Philippines: Bohol, Luzon, and Mindanao. All records are new except for the type localities as well as the presence of *O. ater* in Edam and Mindanao (Hoffmann 1928: 81).

##### Habitat

**Figure 34.**
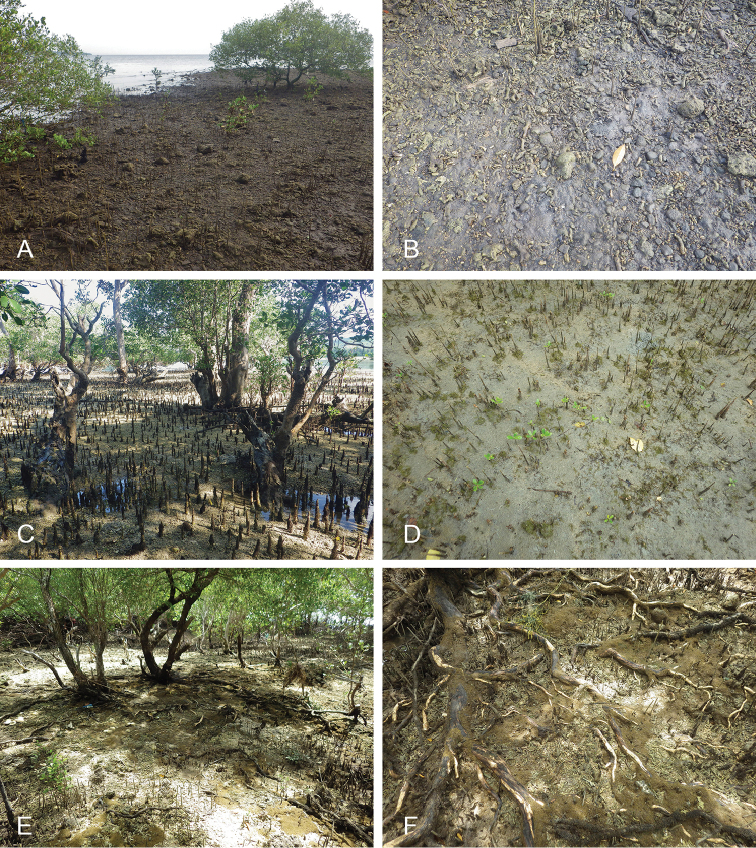
Mangrove habitats, *Wallaconchis ater*. **A–B** Indonesia, Bali, Pemuteran, Labuhan Lalang Harbor, coral rubble, rocks and a few *Avicennia* (st 157) **C–D** Indonesia, Lombok, Don Don, old *Avicennia* forest, with coral rubble (st 149) **E–F** Philippines, Bohol, Mabini, open *Avicennia* and *Sonneratia* mangrove with sand and coral rubble (st 196).

**Figure 35.**
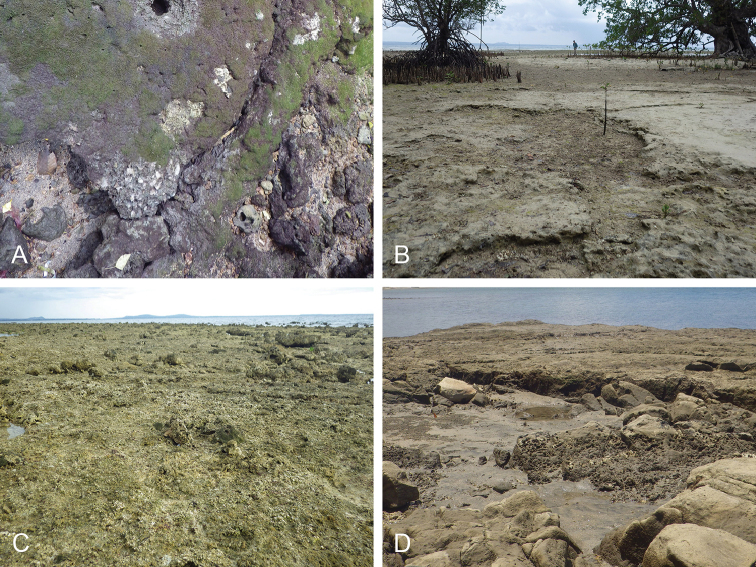
Coral rubble habitats, *Wallaconchis ater*. **A** Indonesia, Sulawesi, Bahoi, sand and small rocks outside a mangrove (st 88) **B** Philippines, Bohol, Maribojoc, uplifted, dead, coral flat covered with sand and algae adjacent to a few mangrove trees (st 202) **C** Philippines, Bohol, Maribojoc, coral rubble of uplifted coral reef (st 191) **D** Indonesia, Halmahera, Foli, rocky shore by beach (st 217).

(Figs 34–35, Table 3). *Wallaconchis ater* is found in the intertidal on coral rubble and small stones. It is often in patches of mangrove trees with coral rubble (Fig. 34), but may also be found in intertidal regions without mangrove trees (Fig. 35).

##### Diagnosis

(Table 5). *Wallaconchis ater* cannot be distinguished from other *Wallaconchis* species based on external features. Grey or brown specimens cannot be distinguished from any other species, and brightly colored specimens cannot be distinguished from *W. nangkauriense* or *W. graniferum*. Internally, the large, coiled penis of *W. ater* with projections at its tip is quite peculiar and is diagnostic.

##### Color and morphology of live animals

**Figure 36.**
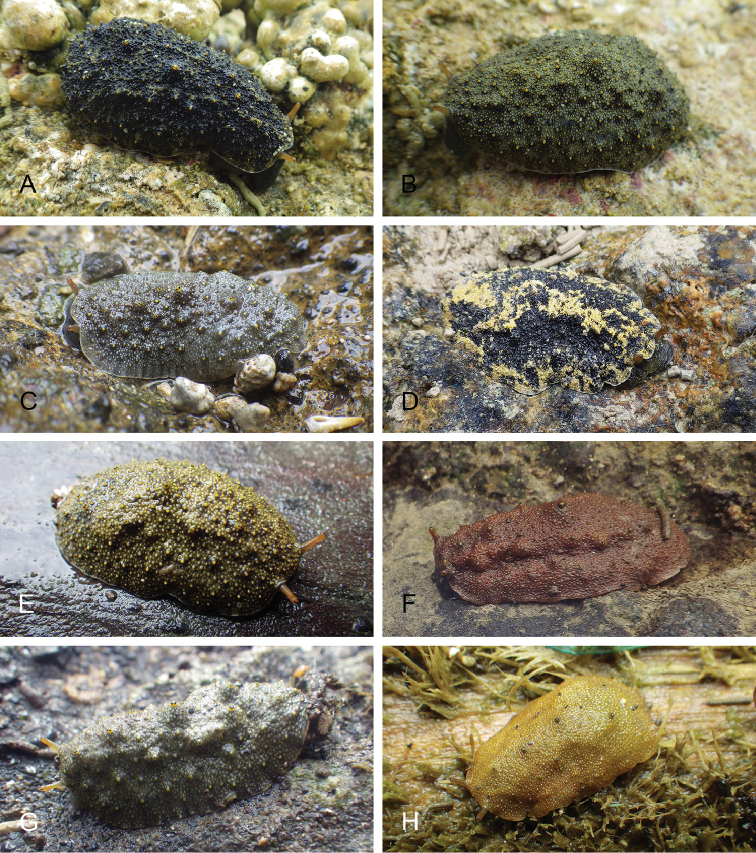
Live specimens, dorsal view, *Wallaconchis ater*. **A** 33 mm long [3265], Philippines, Bohol (PNM 041214) **B** Dorsal view, 34 mm long [3271], Philippines, Bohol (PNM 041222) **C** 26 mm long [3210], Philippines, Luzon (PNM 041213) **D** 26 mm long [3405], Philippines, Bohol (PNM 041219) **E** 26 mm long [2164], Indonesia, Sulawesi (UMIZ 00036) **F** 38 mm long [5057], Indonesia, Halmahera (UMIZ 00046) **G** Dorsal view, 22 mm long [3137], Indonesia, Bali (UMIZ 00053) **H** 25 mm long [2157], Indonesia, Sulawesi (UMIZ 00036).

**Figure 37.**
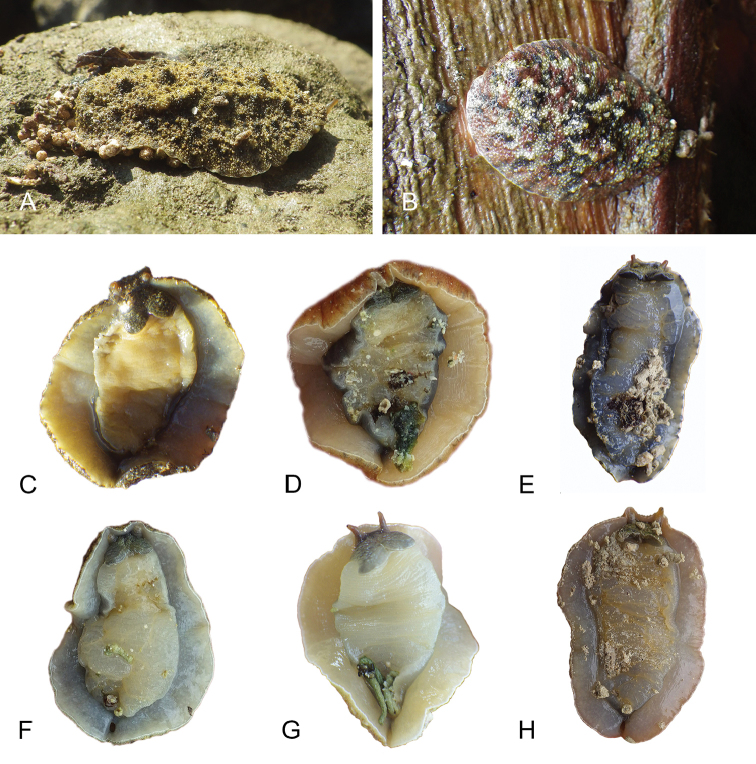
Live specimens, *Wallaconchis ater*. **A** Dorsal view, 43 mm long [2970], Indonesia, Lombok (UMIZ 00043) **B** Dorsal view, 37 mm long [2170], Indonesia, Sulawesi (UMIZ 00036) **C** Ventral view, 35 mm long [2974], Indonesia, Lombok (UMIZ 00043) **D** Ventral view, 36 mm long [3266], Philippines, Bohol (PNM 041214) **E** Ventral view, 26 mm long [3405], Philippines, Bohol (PNM 041219) **F** Ventral view, 33 mm long [3272], Philippines, Bohol (PNM 041214) **G** Ventral view, 32 mm long [3277], Philippines, Bohol (PNM 041222) **H** Ventral view, 37 mm long [3420], Philippines, Bohol (PNM 0412224).

(Figs 36–37). In sandy habitats, sand grains may be stuck to the dorsal notum. The dorsal coloration is highly variable, and often a mottling of two or three colors, of which the most common are brown, black, green, red, grey, and yellow. In some individuals, many small yellow dots are present on the dorsal notum. The color of the ocular tentacles may be yellow-orange, light brown or reddish brown. The hyponotum is cream colored, yellow-orange, yellow-grey or grey. The foot is yellow-grey, yellow-orange, or grey. The lateral sides of the foot may be the same color as the pedal sole (for example, Fig. 37H) or occasionally dark grey (Fig. 37D).

##### External morphology.

Between six and 18 papillae bear dorsal eyes (three or four per papilla). There is a retractable papilla with eyes in the center of the dorsal notum, which may be slightly raised above the dorsal surface.

##### Digestive system

**Figure 38.**
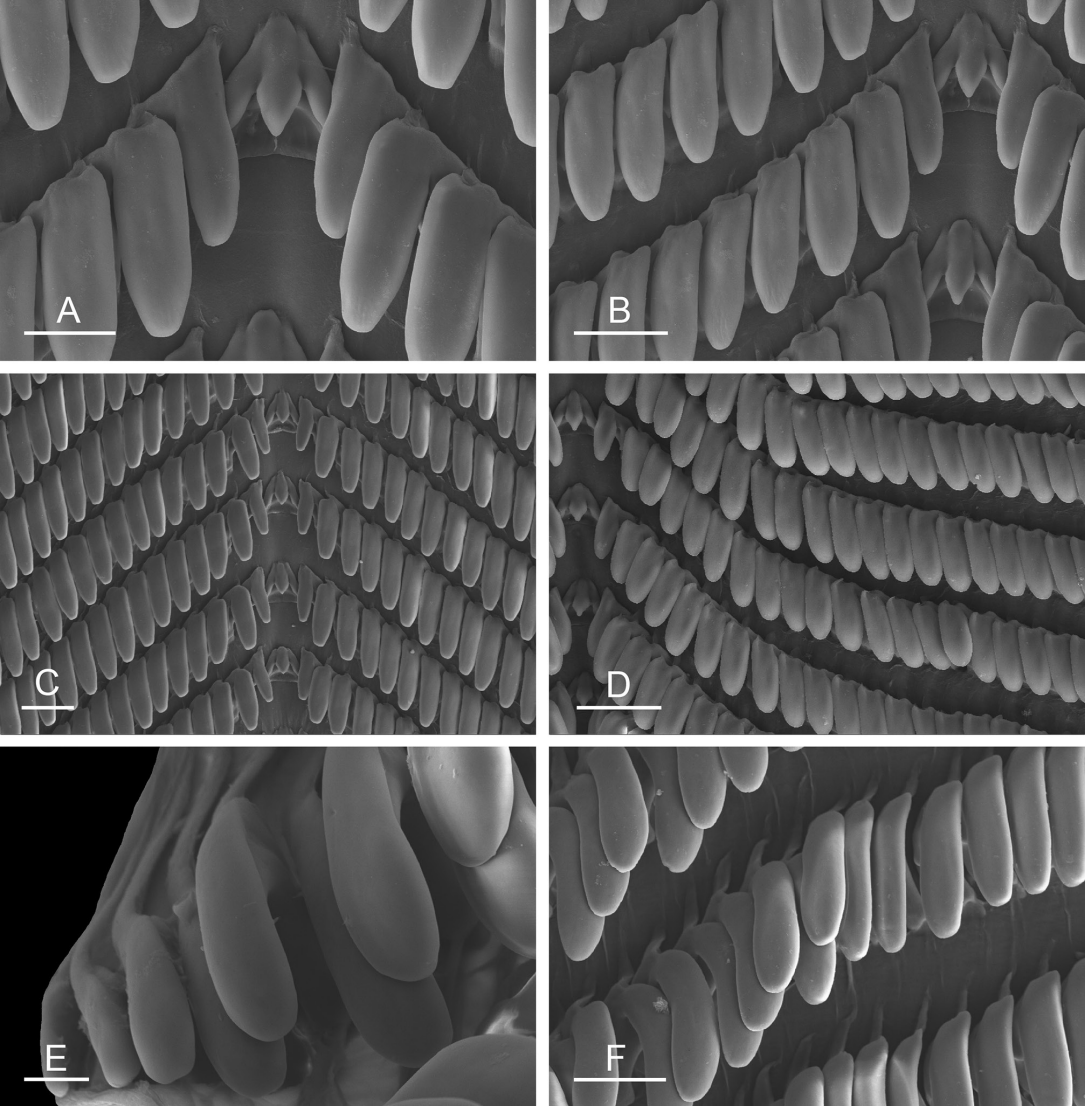
Radula, *Wallaconchis ater*, Indonesia (A–B) Bali [3137] (UMIZ 00053) (D) Sulawesi [2330] (UMIZ 00039) (**C, E–F**) Sulawesi [2220] (UMIZ 00050). **A** Rachidian and innermost lateral teeth, scale bar 20 μm **B** Rachidian and left innermost lateral teeth, scale bar 30 μm **C** Rachidian and innermost lateral teeth, scale bar 50 μm **D** Rachidian and right innermost lateral teeth, scale bar 50 μm **E** Outermost lateral teeth, scale bar 10 μm **F** Transition between inner lateral teeth and outer lateral teeth, scale bar 40 μm.

(Fig. 38, Table 4). Each radular row contains a rachidian tooth and two half rows of lateral teeth. Examples of radular formulae are presented in Table 4. The length of the rachidian teeth is approximately 20–25 µm, significantly smaller than that of the lateral teeth. The length of the hook of the lateral teeth gradually increases, from 40 to 70 µm, from the inner to the outer teeth (excluding the innermost and outermost lateral teeth which are significantly smaller). The intestinal loops are of type I.

##### Reproductive system

**Figure 39.**
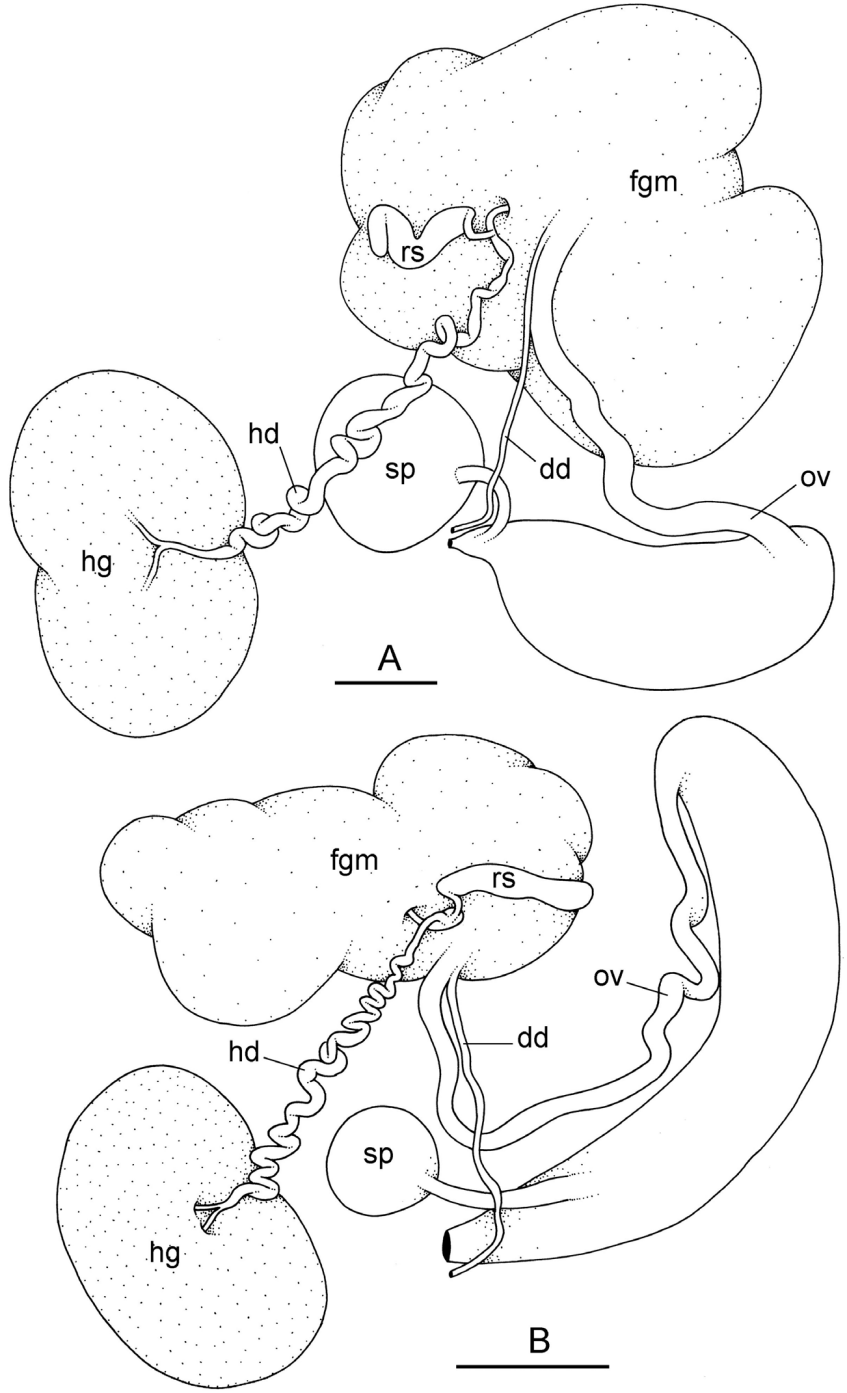
Reproductive system, hermaphroditic (female), posterior parts, *Wallaconchis ater*, Indonesia **A** Lombok, scale bar 2 mm [2970] (UMIZ 00043) **B** Sulawesi, scale bar 3 mm [2220] (UMIZ 00050). Abbreviations: **dd** deferent duct **fgm** female gland mass **hd** hermaphroditic duct **hg** hermaphroditic gland **ov** oviduct **rs** receptaculum seminis **sp** spermatheca.

(Fig. 39). Posteriorly, the oviduct is larger than the deferent duct and is especially wide distally. The spermatheca is spherical and enters the distal end of the oviduct through a short duct. In small specimens (less than 17 mm long) female reproductive organs are small, with a narrower oviduct.

##### Copulatory apparatus

**Figure 40.**
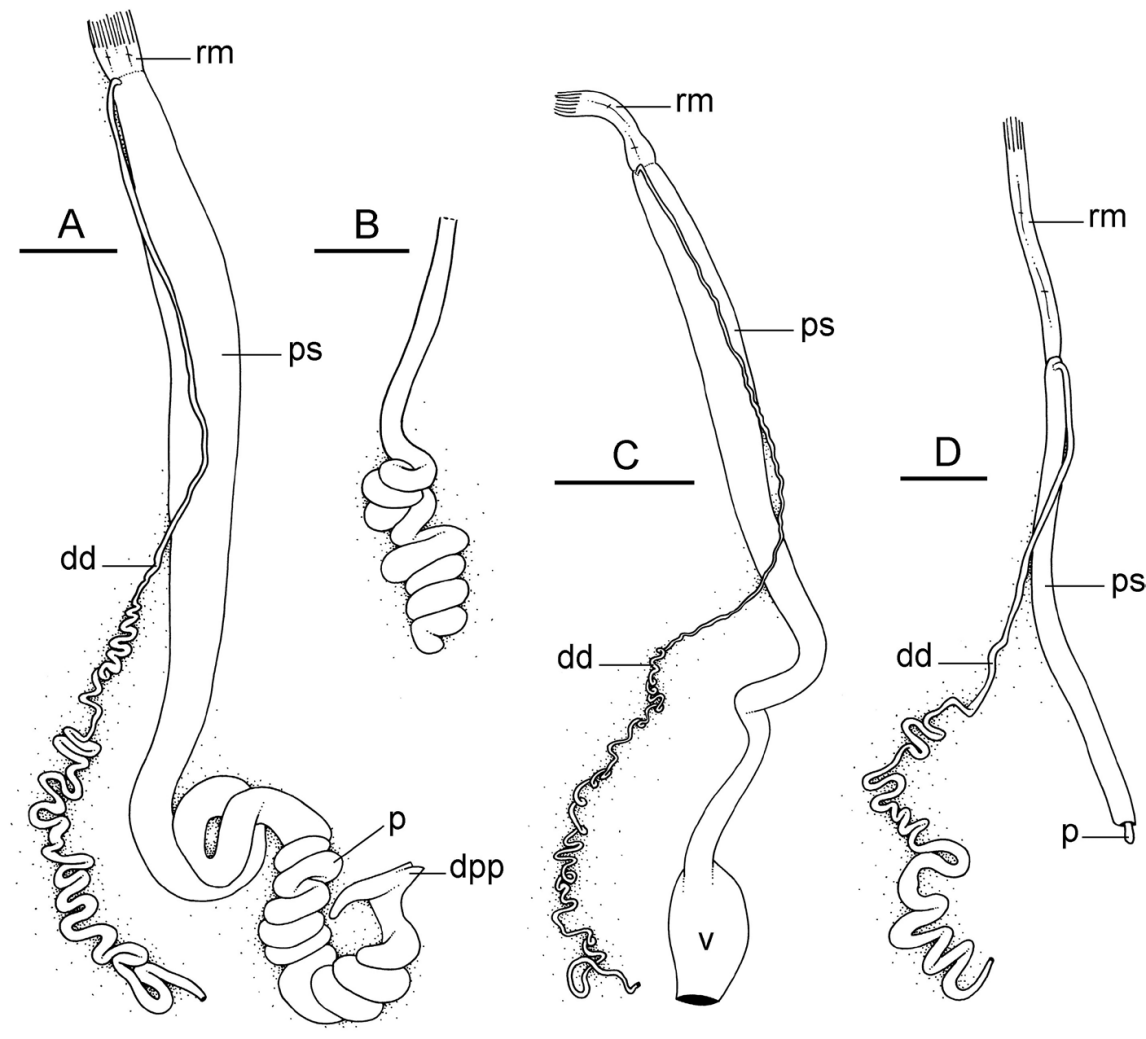
Reproductive system, *Wallaconchis ater*, Indonesia. **A** Anterior, male copulatory parts (vestibule removed, showing the coiled and U-shaped penis), Halmahera, scale bar 2 mm [5078] (UMIZ 00047) **B** Penis (coil of loops removed from vestibule), Sulawesi, scale bar 2 mm [2283] (UMIZ 00038) **C** Anterior, male copulatory parts, scale bar 5 mm, same as B **D** Anterior, male copulatory parts, Kei Islands, scale bar 1 mm [2939] (UMIZ 00041). Abbreviations: **dd** deferent duct **dpp** distal penial projections **p** penis **ps** penial sheath **rm** retractor muscle **v** vestibule.

**Figure 41.**
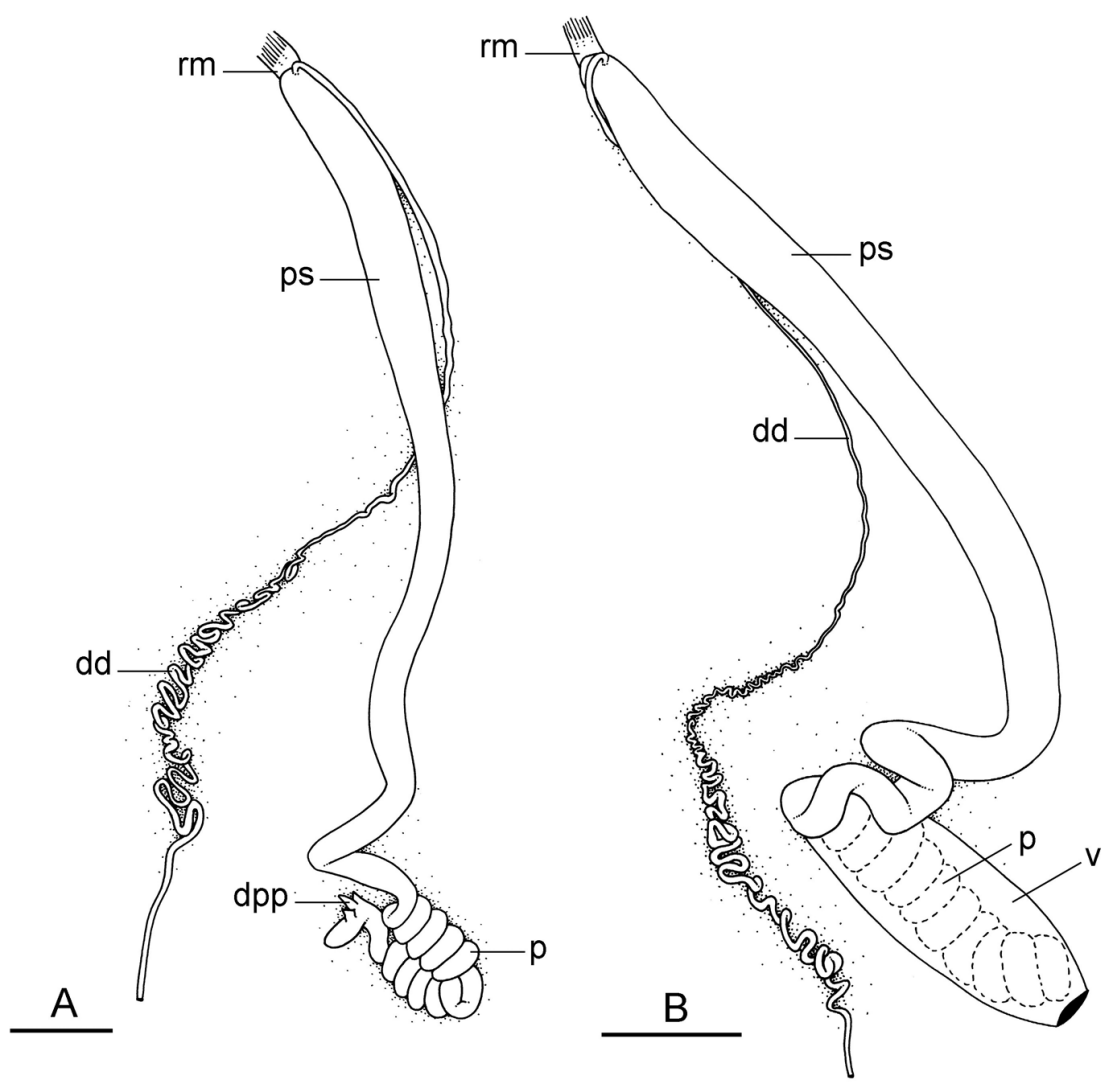
Reproductive system, anterior, male copulatory parts, *Wallaconchis ater*. **A** Vestibule removed, showing the coiled and U-shaped penis, Indonesia, Sulawesi, scale bar 3 mm [2220] (UMIZ 00050) **B** Vestibule with coiled penis inside, Philippines, Bohol, scale bar 3 mm [3270] (PNM 041220). Abbreviations: **dd** deferent duct **dpp** distal penial projections **p** penis **ps** penial sheath **rm** retractor muscle **v** vestibule.

**Figure 42.**
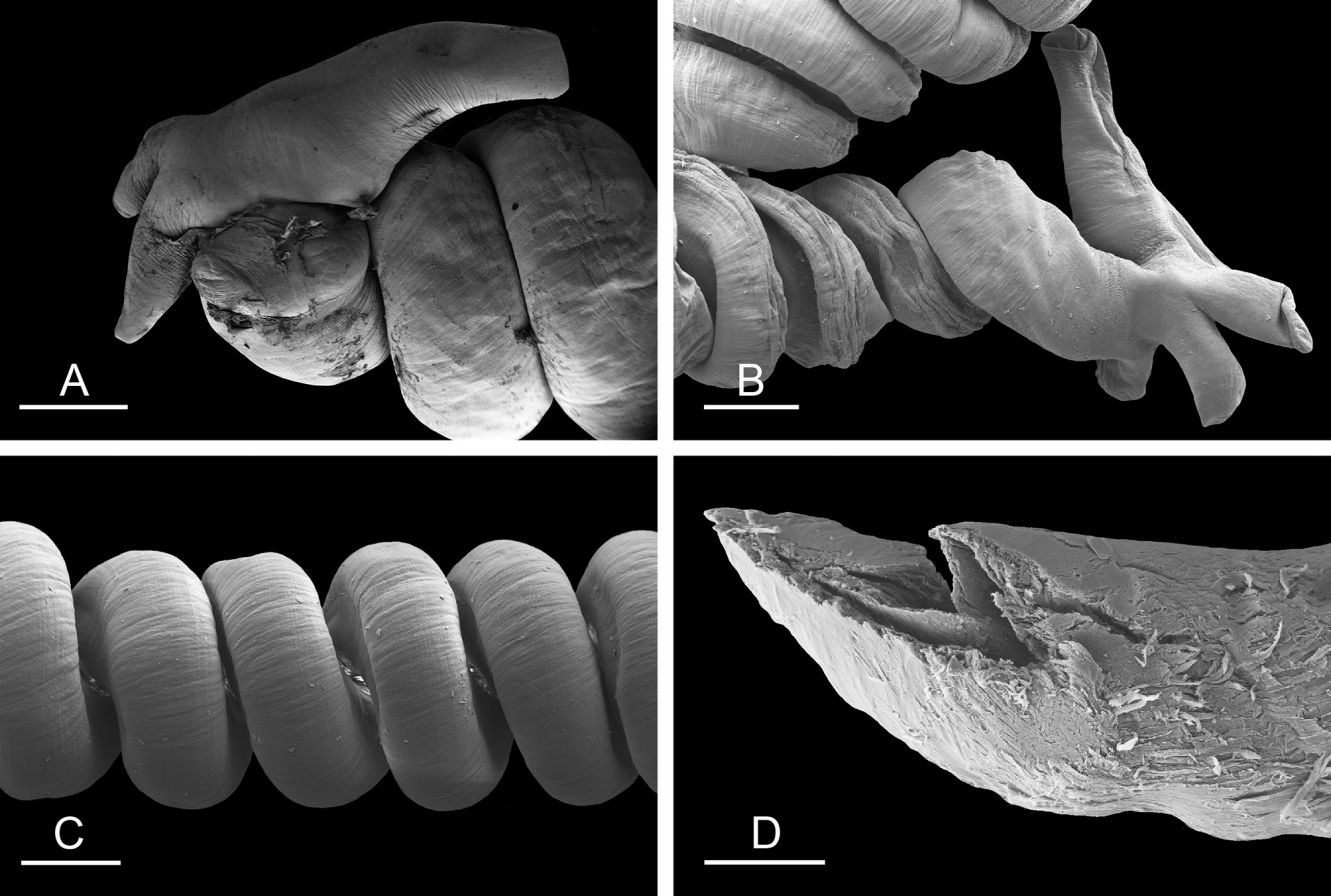
Penis, *Wallaconchis ater*, Indonesia. **A** Detail of distal portion when fully relaxed (with distal penial projections) and partial view of the coil, Indonesia, Halmahera, scale bar 500 μm [3393] (PNM 041218) **B** Detail of distal portion when fully relaxed (with distal penial projections) and partial view of the coil, Halmahera, scale bar 500 μm [5125] (UMIZ 00054) **C** Partial view of the coil (with six loops), Philippines, Bohol, scale bar 500 μm [3266] (PNM 041214) **D** Cross-section showing the longitudinal fold, Bali, scale bar 300 μm [3137] (UMIZ 00053).

(Figs 40–42). The penis is smooth. It does not bear any hooks. In mature specimens (> 22 mm long), it is tightly coiled (Fig. 42C), with approximately eight to 15 loops in the coil (Figs 40A, B, 41). However, the number of loops in the coil is correlated with the body length (and sexual maturity) and there are fewer (less than five) or no loops in shorter (< 22 mm long) and less mature specimens (Fig. 40D). When the tip of the penis is evaginated, it is tapered distally and bears three pointed projections (Figs 40A, 41A, 42A, B). The penis is enclosed within the vestibule (Figs 40C, 41B), and the coil may form a U-shape within the vestibule (Fig. 41A). The penis is sometimes longitudinally folded and it appears that its loops could expand in diameter (cross-section of penis, Fig. 42D). The vestibule is exceptionally large (it can be nearly half the length of the body cavity). The penial sheath is long and usually forms a loop within the body cavity. The deferent duct is exceptionally narrow and convoluted, and only becomes wider distally in the anterior region of the body cavity (Fig. 40). In small specimens, the deferent duct is less convoluted than in larger (and more mature) specimens. The retractor muscle is short (approximately 2 mm) and inserts on the posterior body wall, on the right side of the rectum.

##### Remarks.

Lesson’s original description of *Onchidium ater* was brief, mostly based on the external morphology, and lacked illustrations of the internal anatomy. Fortunately, some organs remain in the syntypes which display the diagnostic characters of *Wallaconchis* (intestinal loops of type I and no rectal gland).

The male parts were removed from both syntypes by Labbé when he re-described them for his 1934 monograph. Therefore, only Lesson’s original description of the male parts and their re-description by Labbé can be used to determine the application of the name *O. ater*. Lesson (1830: 300, our translation from French) describes the penis as a “very elongated, cylindrical, twisted upon itself, and very contractile tube in the anterior part of the body.” Also, Labbé’s (1934: fig. 30) illustration of the penis of the syntypes of *O. ater* reveals that it consists of a coil of tight loops, which perfectly matches the penis of the species described here. In addition, the wide oviduct in the syntypes is consistent with this species. As a result, we apply the name *O. ater* to the species described here.

Labbé (1934: 206) also described “feathery gills” in the two syntypes of *O. ater*, which, as a result, he transferred to the genus *Scaphis*. Labbé commonly made surprising mistakes, describing features in specimens in which they were clearly not present. In this case, there are no gills on the dorsal notum of the syntypes of *O. ater*. Labbé also described a penial accessory gland in the syntypes of *O. ater*. Because the male parts are missing (Labbé removed them), it is something that we cannot verify. However, given that there is no known onchidiid species in any other genus with this type of penial anatomy, Labbé’s description of an accessory penial gland is regarded as one of his numerous mistakes. Because of the errors he made when he re-described the two syntypes of *O. ater*, only Labbé’s (1934: fig. 30) illustration of the penis is cited as a description of *W. ater* in the list of references above.

The unique combination of characters observed on the lectotype of *Onchidium keiense* (intestinal loops of type I, no rectal gland, male opening below the right tentacle, and no accessory penial gland) indicates that *O. keiense* belongs to *Wallaconchis*. The re-examination of the lectotype and most paralectotypes available for *O. keiense* revealed no difference with the two syntypes of *O. ater* for the penial anatomy and the enlarged oviduct, therefore *O. keiense* is identified here as a junior synonym of *W. ater*. However, not all paralectotypes of *O. keiense* are part of *W. ater*: at least one paralectotype of *O. keiense* belongs to the genus *Peronia* (due to the presence of an accessory gland and a different intestinal type). The designation of the lectotype makes it clear that the name *O. keiense* applies to the species described by Hoffmann without an accessory gland.

Hoffmann (1926: 23) wrote that *O. keiense* was very close to *O. ater*, but he was not convinced that the two were the same species because he thought that the cylindrical “organe excitateur” that Lesson described could have either been the penis or an accessory penial gland. If Hoffmann had been able to examine the syntypes of *O. ater*, he would have seen that the penial morphology is identical to *O. keiense*. Hoffmann’s description of *O. keiense* is consistent with *Wallaconchis ater*, except for the description of dorsal eyes. The dorsal notum of *W. ater* bears many papillae with three or four dorsal eyes on each papilla, while Hoffmann described 12 large papillae on the dorsal notum of *O. keiense* with one dorsal eye each, and the central papillae with three or four eyes. Although the papillae are retracted in the lectotype and many paralectotypes, the presence of three or four dorsal eyes on all visible papillae was confirmed.

Hoffmann (1928: 81) examined additional specimens (he does not indicate how many) from Mindanao (Philippines) and Edam (a small Indonesian island near Jakarta) which he identified as *O. keiense* in his subsequent revision of the Onchidiidae. Hoffmann did not comment on the anatomy of these additional specimens (which were not examined for the present study). However, given that he is the original author of *O. keiense*, it can be assumed that he recognized the distinct penial anatomy of this species. Mindanao is within the geographic range of *W. ater*. However, the presence of *W. ater* in Edam slightly expands to the west the geographic range delineated here based on our collections and expands the known distribution of *W. ater*.

Hoffmann (1926: 24, our translation from German) considered creating a new genus for *Onchidium keiense*: “The peculiarity of the penis could almost suggest the idea that we are dealing here with a whole new genus. In the genus *Onchidium* [spelled *Oncidium*], however, there are already species which differ quite considerably from each other, so that I have no reason to create a new genus for my species.” Hoffmann recognized the similarity between some *Onchidium* species transferred here to *Wallaconchis* (i.e., *O. buetschlii*, *O. nangkauriense*, and *O. ovale*), but he did not comment on the similarity between *O. keiense* and these species. However, in his identification key, Hoffmann (1928: 109–110) listed *O. keiense* in the same part of the key as the *Onchidium* species transferred here to *Wallaconchis*, using exactly the same characters: no accessory penial gland, no rectal gland, and intestinal loops of type I. So, even though Hoffmann showed in his identification key that those species are similar, he did not create or name any group for them. Also, in that same part of the key, Hoffmann listed *O. papuanum* and *O. palaense*. He must have assumed that the intestinal loops in these two species are of type I because there is no indication in the original descriptions that their intestinal loops are actually of type I and Hoffmann did not comment on the type material (which is lost for both species). However, the position of the male opening in the original descriptions of *O. papuanum* and *O. palaense* (to the left of right tentacle) indicates that these two names do not apply to *Wallaconchis* species.

Hoffmann (1926:23) wrote that *W. keiense* seemed to be like *Onchidium cinereum* Quoy & Gaimard, 1832, but his comparison was based on Semper’s (1880: pl. 20, fig. 11, pl. 23, fig. 13; 1882:286) description of new specimens from Tonga-tabu [Tongatapu Island, Tonga] which he received from the Museum Godeffroy, instead of Quoy and Gaimard’s original type material (from Tonga). The type material of *O. cinereum* could not be located. *Onchidium cinereum* should likely be regarded as a *nomen dubium* because and it is unclear to which genus it belongs. Hoffmann also found an enlarged oviduct in *Onchidium aberrans* Semper, 1882 similar to the oviduct found in *O. keiense*, but *O. aberrans* does not belong to *Wallaconchis* as it lacks the diagnostic characters of *Wallaconchis*.

Hoffmann did not illustrate the coiled penis of *O. keiense* (the penial complex was illustrated with the vestibule and did not show the loops inside), which made it difficult for others to identify the diagnostic character of *O. keiense*. Labbé did not recognize the similarity between the syntypes of *O. ater* which he examined and Hoffmann’s description of *O. keiense*. Labbé moved *O. keiense* to his genus *Paraoncidium* Labbé, 1934, which he created for a series of *Onchidium* species with no accessory gland and no rectal gland, but he did not examine any new material for *O. keiense*. Note that *Paraoncidium* is a junior synonym of *Onchidina* (Dayrat and Goulding 2017).

Another species, *Onchidium ferrugineum* Lesson, 1830 was described from the same type locality as *O. ater* (Manokwari, West Papua, Indonesia). The number of syntypes was not included in Lesson’s original description, but Labbé (1934: 214) indicates that he examined four syntypes, one of which was “completely emptied of its viscera.” There are now only three syntypes left in the jar (36/25 mm, 35/19 mm, and 30/18 mm), suggesting that one of the syntypes was lost (MNHN 22951). Lesson’s (1830: 301) description of the penis of *O. ferrugineum* as cylindrical and very twisted is remarkably similar to his description of the penis of *O. ater*. However, Lesson also clearly specifies that gills are present on the dorsal notum. A re-examination of the three syntypes of *O. ferrugineum* shows that they are part of two different genera: the largest syntype (36/25 mm) bears gills (only found in species of the genus *Peronia*) while the two other syntypes do not. To clarify the application of *O. ferrugineum*, the largest syntype with gills is designated here as the lectotype (and now clearly labeled in the jar). Therefore, the name-bearing type of *O. ferrugineum* is part of a *Peronia* species. Both paralectotypes display the diagnostic characters of *Wallaconchis*, and the penis left in one of the paralectotypes is identical to the large, coiled penis of *W. ater* and *W. keiense*. However, because the paralectotypes no longer have any name-bearing function, *O. ferrugineum* applies to a *Peronia* species and cannot apply to a *Wallaconchis* species. Because Labbé’s re-description is based on specimens from two different species (and two different genera), only Labbé’s (1934: fig. 50) illustration of the penis of a paralectotype of *O. ferrugineum* is cited as a description of *W. ater* in the list of references above.

Bretnall (1919) translated Lesson’s description of *O. ater* but did not examine any new material. Gray (1850) and Adams and Adams (1858) mentioned the name *Oncidiella nigra* Lesson, but, as Tapparone-Canefri (1883) first suggested, Lesson did not describe a species with this name, and these authors likely made the error of using the name *O. nigra* for *O. ater* (both *ater* and *nigra* mean black in Latin). Gray (1850) did not include a description to accompany the name *O. nigra*, but included an illustration (pl. 181, fig. 7) of the dorsal notum of an animal which could be a *Wallaconchis* species but cannot be positively identified. Adams and Adams (1858) only listed the name *O. nigra* without commenting on it. Finally, Hoffmann (1928) and Labbé (1934) adopted Tapparone-Canefri’s conclusion that *O. nigra* was mistakenly used to refer to *O. ater*.

#### Wallaconchis graniferum

Taxon classificationAnimaliaSystellommatophoraOnchidiidae

(Semper, 1880) comb. n.

**Figure 43.**
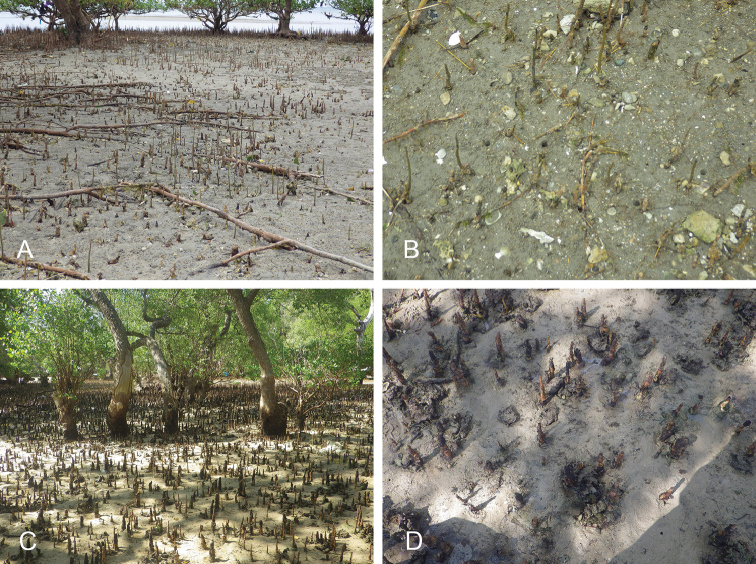
Habitats, *Wallaconchis graniferum*. **A** Philippines, Bohol, Loay, mostly sand, very few rocks, and a few small *Avicennia* trees (st 198) **B** Close up of sand with shells and *Avicennia* roots (same locality as A) **C** Indonesia, Timor, Oesapa, sandy area of mangrove with *Avicennia* and *Sonneratia* trees spread out **D** Close up of sediment and onchidiid tracks (same locality as C).

**Figure 44.**
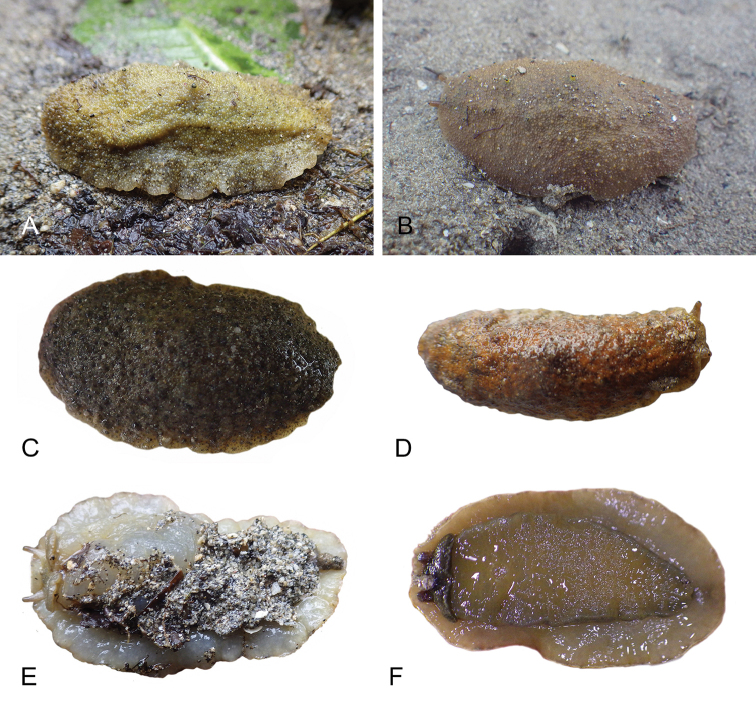
Live specimens, *Wallaconchis graniferum*, Philippines. **A** Dorsal view, 46 mm long [3163], Luzon (PNM 041227) **B** Dorsal view, 30 mm long [5902], Indonesia, Timor (UMIZ 00073) **C** Dorsal view, 28 mm long [3636], Bohol (PNM 041228) **D** Dorsal view, 26 mm long [3635], Bohol (PNM 041228) **E** Ventral view, same as A, **F** Ventral view, same as B.

**Figure 45.**
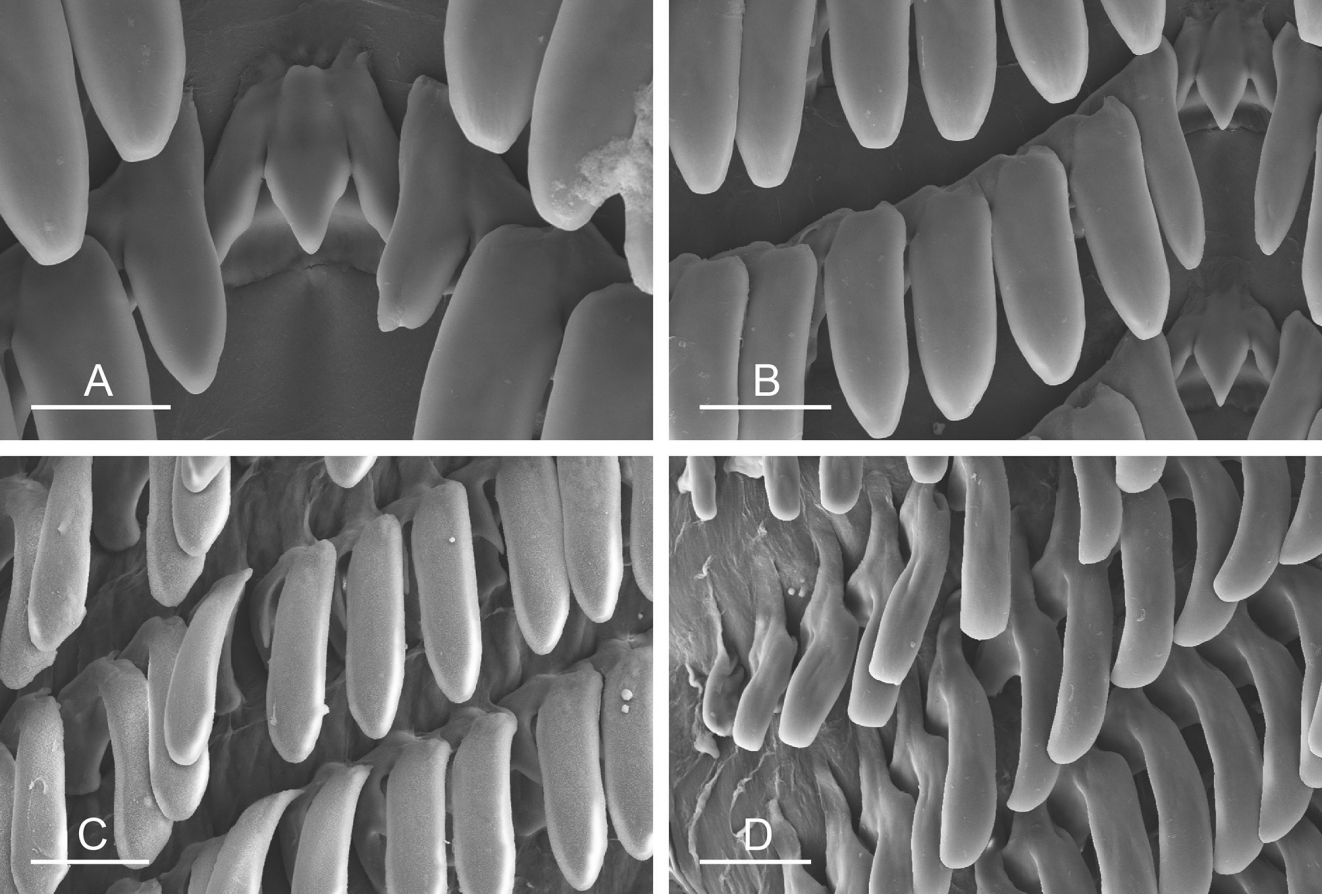
Radula, *Wallaconchis graniferum*, Philippines. **A** Rachidian and innermost lateral teeth, Bohol, scale bar 20 μm [3636] (PNM 041228) **B** Rachidian and innermost lateral teeth, Luzon, scale bar 30 μm [3163] (PNM 041227) **C** Transition between inner lateral teeth and outer lateral teeth, Bohol, scale bar 30 μm [3635] (PNM 041228) **D** Outermost lateral teeth, Bohol, scale bar 30 μm [3636] (PNM 041228).

**Figure 46.**
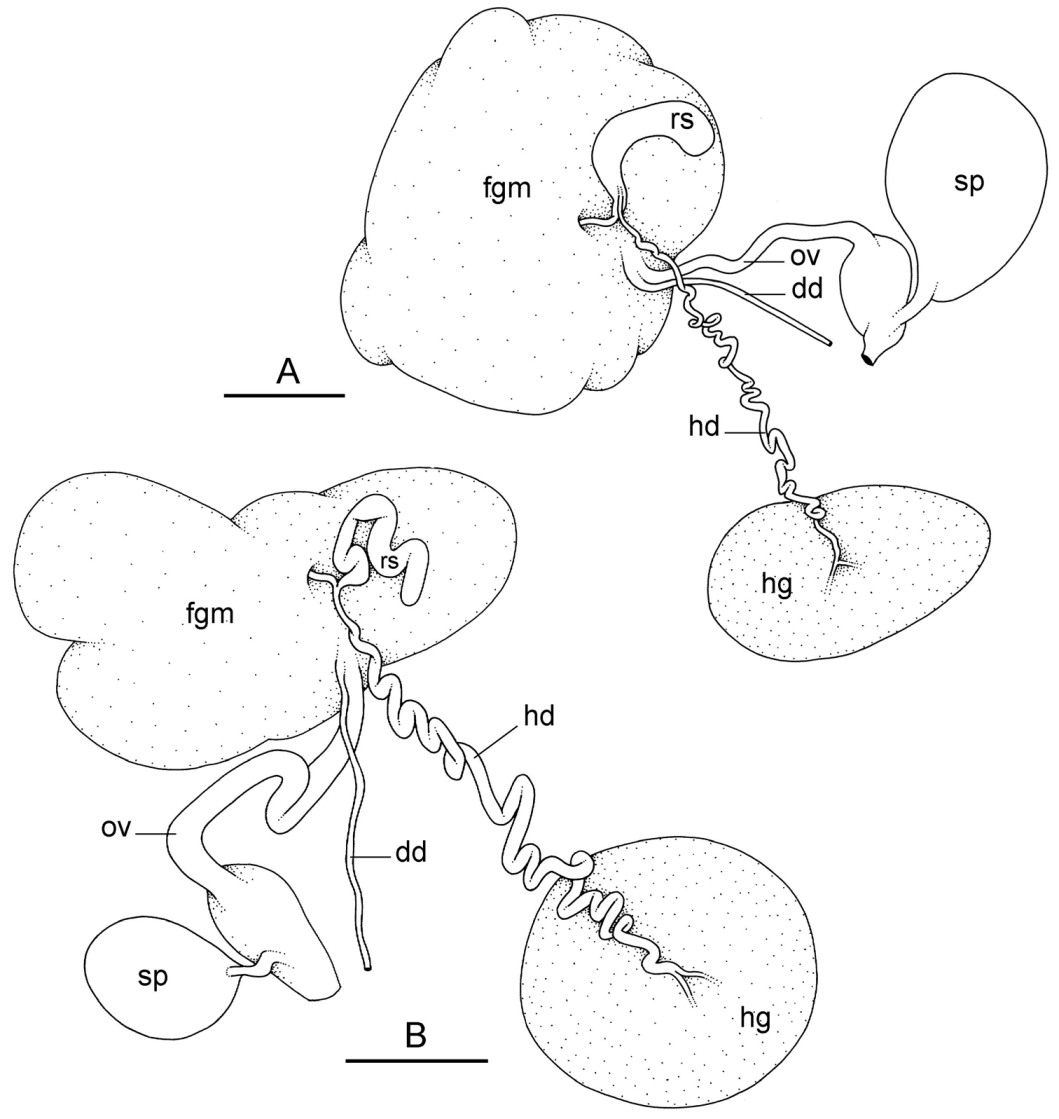
Reproductive system, Hermaphroditic (female), posterior parts, *Wallaconchis graniferum*, Philippines. **A** Luzon, scale bar 5 mm [3163] (PNM 041227) **B** Bohol, scale bar 2 mm [3638] (PNM 041225). Abbreviations: **dd** deferent duct **fgm** female gland mass **hd** hermaphroditic duct **hg** hermaphroditic gland **ov** oviduct **rs** receptaculum seminis **sp** spermatheca.

**Figure 47.**
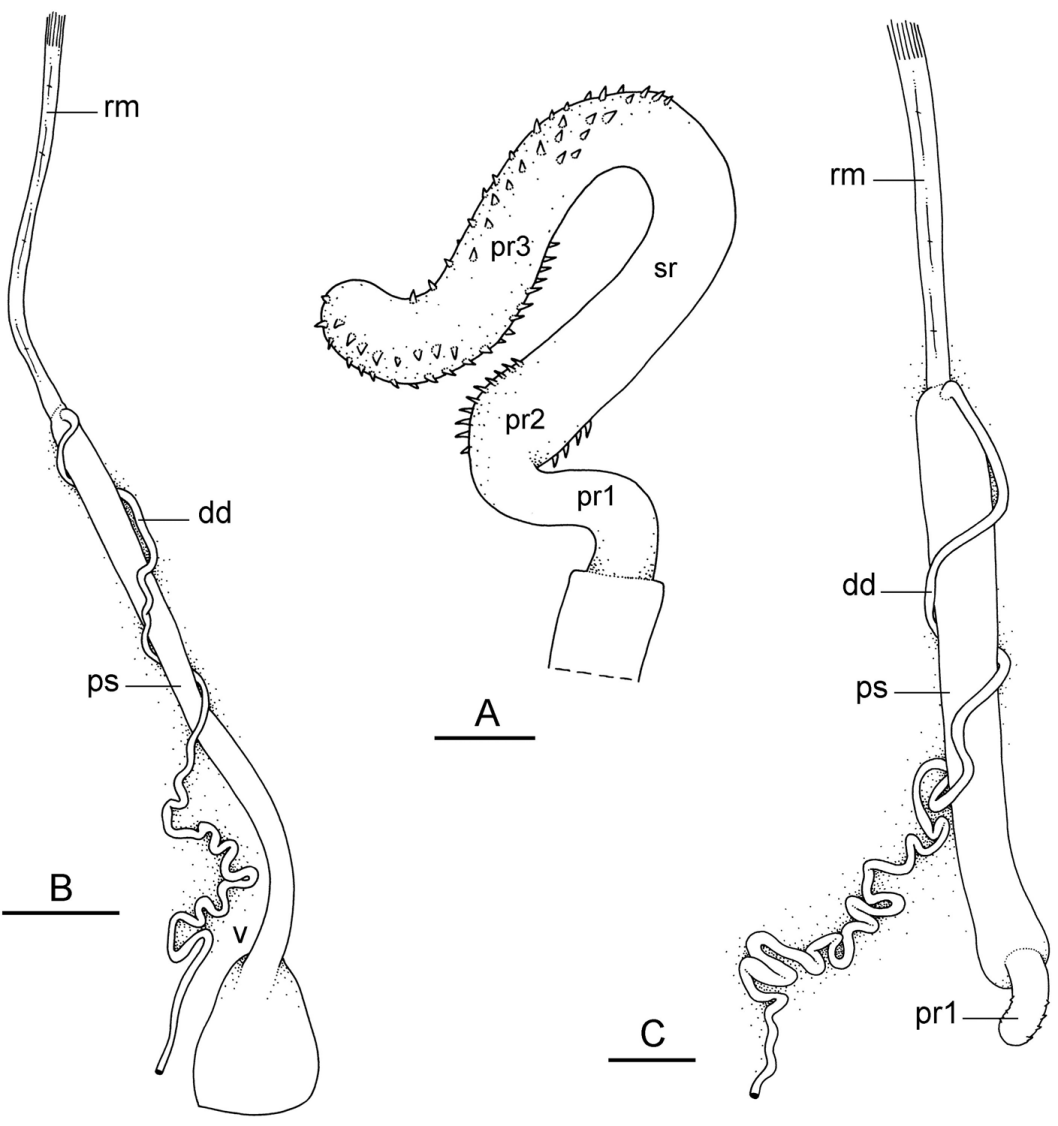
Reproductive system, anterior, male copulatory parts, *Wallaconchis graniferum*, Philippines. **A** Penis with two proximal regions of hooks separated from a third distal region of hooks by a gap with no hooks (the hooks of the first, most proximal region are on the other side of the penis and cannot be seen here), Luzon, scale bar 0.7 mm [3163] (PNM 041227) **B** Anterior male copulatory parts scale bar 3.6 mm same as A. **C** Penis partially evaginated inside the vestibule (removed) Bohol scale bar 1 mm [3638] (PNM 041225). Abbreviations: **dd** deferent duct **pr1** first most proximal penial region (with hooks on the other side) **pr2** second proximal penial region (with hooks) **pr3** third distal penial region (with hooks) **ps** penial sheath **rm** retractor muscle **sr** smooth region of the penis (with no hooks) **v** vestibule.

**Figure 48.**
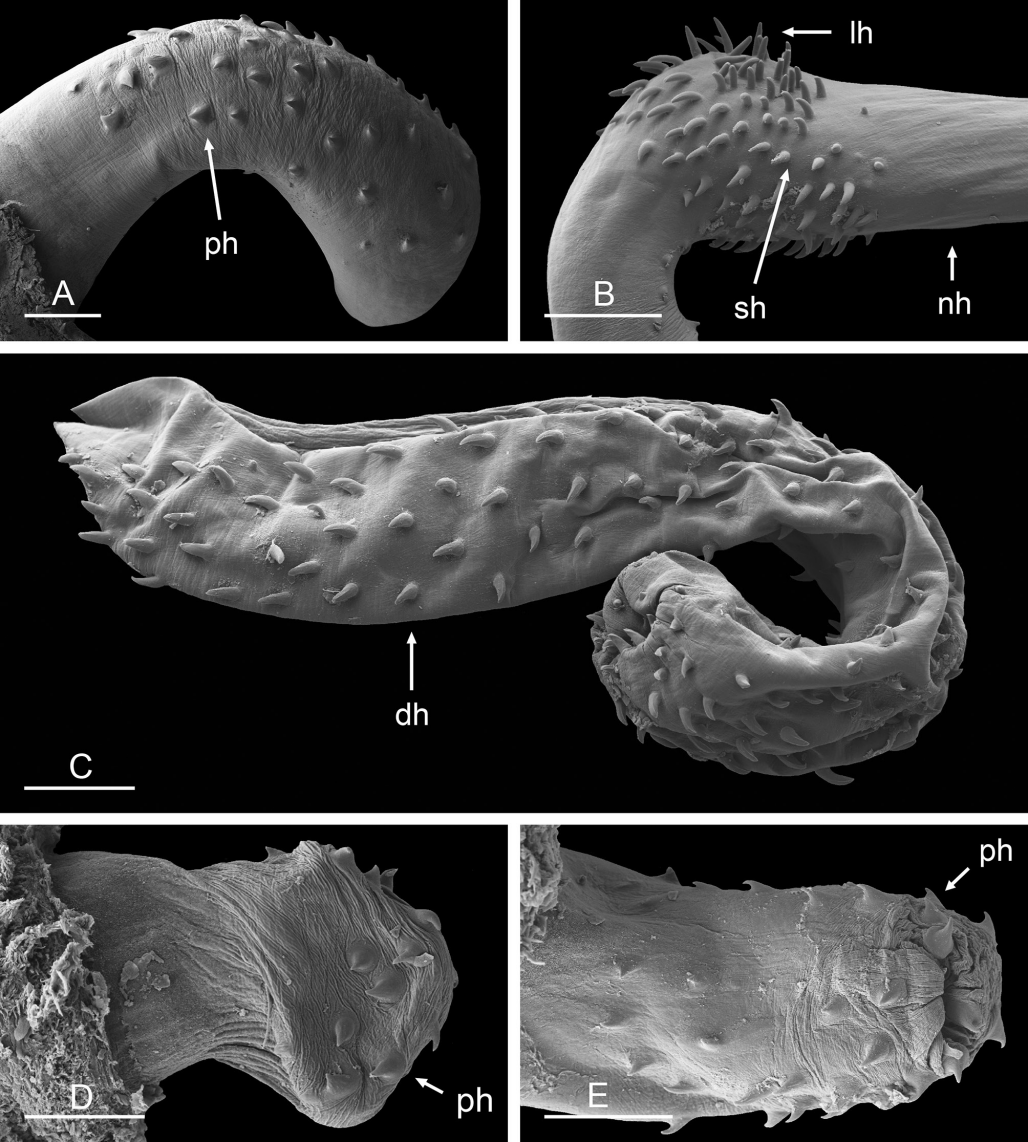
Evaginated penis, *Wallaconchis graniferum*, Philippines (**A–C**) Luzon [3163] (PNM 041227) (**D**) Bohol [3636] (PNM 041228) (E) Bohol [3638] (PNM 041225). **A** Proximal region of evaginated penis with hooks, scale bar 400 μm **B** Middle region of evaginated penis with long slender hooks on one side, and shorter hooks on the other side, scale bar 400 μm **C** Distal region of evaginated penis with distal hooks, scale bar 300 μm **D** Partially evaginated penis with proximal hooks (more distal hooks are inside the rest of the invaginated penis), scale bar 200 μm **E** Partially evaginated penis with most proximal hooks (more distal hooks are invaginated inside), scale bar 200 μm. Abbreviations (from proximal to distal): **ph** proximal hooks (first penial region) **lh** long slender hooks (second penial region) **sh** short hooks (second penial region) **nh** no hooks (gap) **dh** distal hooks (third penial region).

**Figure 49.**
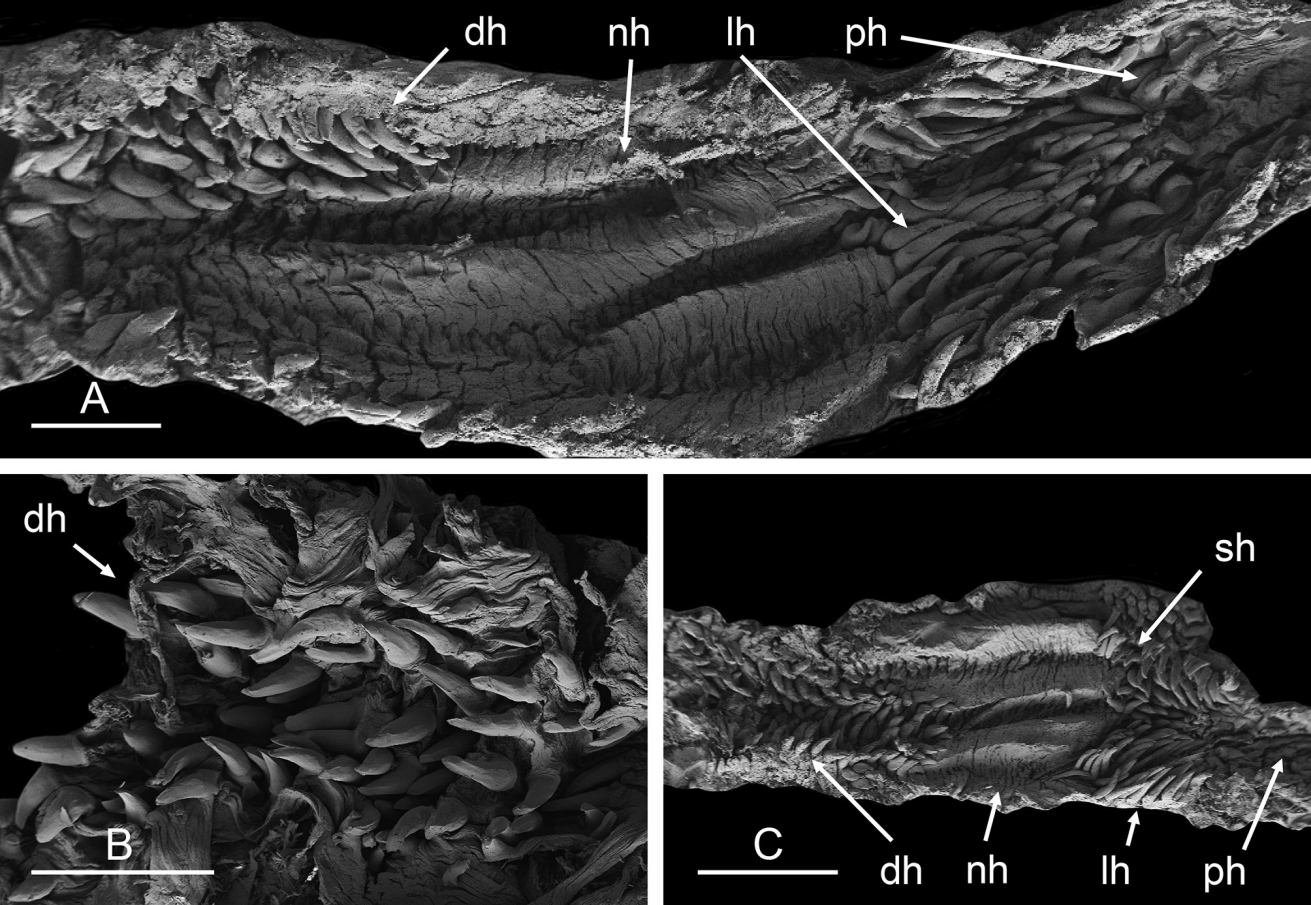
Penis, *Wallaconchis graniferum*, not evaginated and opened, Philippines, Bohol (**A–B**) [5762] (PNM 041226) (**C**) [5761] (PNM 041228). **A** Penis invaginated inside the penial sheath opened to reveal regions of penial hooks, scale bar 200 μm **B** Most proximal region of penis invaginated inside the penial sheath opened to reveal penial hooks, scale bar 200 μm **C** Penis invaginated inside penial sheath (both opened to reveal the penial hooks), scale bar 0.5 mm. Abbreviations (from proximal to distal): **ph** proximal hooks (first penial region) **lh** long slender hooks (second penial region) **sh** short hooks (second penial region) **nh** no hooks (gap) **dh** distal hooks (third penial region).

Figs 43 , 44 , 45 , 46 , 47 , 48 , 49

Onchidium graniferum Semper, 1880: pl. 19, fig. 13, pl. 23, fig. 3; Semper 1882, 273–274, pl. 21, fig. 10; Hoffmann 1928: 80–81 (as Oncidium graniferum).
Paraoncidium reevesii : Britton 1984: 184–185, fig. 4 [non Onchidium reevesii J. E. Gray, 1850].
Onchidella sp.: Sun et al. 2014: fig. 4 [non Onchidella J. E. Gray, 1850].

##### Type locality.

Bohol (Philippines).

##### Type material.

Three syntypes (ZMB 39032), between 22/14 mm and 11 mm, according to Semper (no width was provided by Semper for the smaller specimen). The syntypes were destroyed prior to the present study, and it is only through Semper’s description that it could be ascertained that there were originally three syntypes. Only small pieces of dorsal notum and of one digestive system (stomach, intestine, and digestive glands) now remain.

##### Additional material examined.

**Indonesia**, Timor, Oesapa, 10°08.73'S, 123°38.10'E, 1 specimen 30/19 mm [5902], st 250, sandy part of mangrove with *Sonneratia* and *Avicennia* trees (UMIZ 00073). **Philippines**, Luzon, Lian, Batangas, 13°59.76'N, 120°37.43'E, 1 specimen 46/27 mm [3163], st 181, sandy, open *Avicennia* forest (PNM 041227); Bohol, Loay, 09°36.23'N, 123°59.72'E, 6 specimens 28/17 mm [3636], 26/18 mm [3638], 26/15 mm [3635], 18/12 mm [5760], 16/13 mm [5762] and 16/11 mm [5761], st 198, mostly sand, and a few *Avicennia* (PNM 041228).

##### Distribution.

Indonesia: Timor. Philippines: Bohol (type locality) and Luzon. China. All records are new except for the type locality.

##### Habitat

(Fig. 43, Table 3). In Bohol, *Wallaconchis graniferum* was found on a sandy beach with a few *Avicennia* trees; the sand was finely grained, mixed with many broken shells, and sculpted into round pellets by sand bubbler crabs. In Luzon, it was found on fine sand in a coastal mangrove forest, near the roots of an *Avicennia* tree. In Timor, one animal was found in a sandy area of a *Sonneratia* and *Avicennia* mangrove.

##### Diagnosis

(Table 5). *Wallaconchis graniferum* cannot be distinguished externally from other *Wallaconchis* species. Brown specimens cannot be distinguished from any other species, and brightly-colored specimens cannot be distinguished from *W. nangkauriense* and *W. ater*. The peculiar pattern of penial hooks of *W. graniferum* (two proximal regions of hooks separated from a distal region of hooks by a gap without hooks) has not been observed in any other onchidiid genus.

##### Color and morphology of live animals

(Fig. 44). The dorsal color is variable and may be brown, yellow or orange but additional color variants may exist. The ocular tentacles are white, beige, yellow-orange, or orange-brown. The hyponotum is white or beige-orange. The foot is orange or yellowish white.

##### External morphology.

Approximately three to twelve papillae bear dorsal eyes, with three or four eyes per papilla. There is a retractable papilla with eyes in the center of the dorsal notum, which may be slightly raised above the dorsal surface.

##### Digestive system

(Fig. 45, Table 4). Examples of radular formulae are presented in Table 4. The length of the rachidian teeth is approximately 25–30 µm, significantly smaller than that of the lateral teeth. The length of the hook of the lateral teeth gradually increases, from 40 to 60 µm, from the inner teeth to the outer teeth (excluding the innermost and outermost lateral teeth which are significantly smaller). The intestinal loops are of type I.

##### Reproductive system

(Fig. 46). In the posterior (hermaphroditic) part of the reproductive system, the oviduct is larger than the deferent duct and is especially wide distally. The spermatheca is spherical and joins the distal end of the oviduct through a short duct. In the specimen from Luzon, the spermatheca was larger than in the specimens from Bohol.

##### Copulatory apparatus

(Figs 47–49). The penis bears hooks in three distinct regions, two proximal regions being separated from one distal region by a gap with no hooks (Fig. 47A). When the penis is partially evaginated, only the hooks near the base can be observed (Figs 47C, 48D, E). In large individuals, the penis may reach approximately 8 mm in length when fully evaginated (Fig. 48A–C). In the first, most proximal region (at the base of the penis), the hooks are small, between 50 and 90 μm long, and clustered on one side of the penis. Following these hooks, a second region bears slender hooks, closely packed together, which vary in size. They may be longer on one side of the penis (between 90 and 170 μm, Figs 48B, 49C) than on the other side (between 40 and 50 μm). Then the penis is smooth (with no hooks) (Figs 48B, 49A, C). Finally, distally, at the tip of the penis, a fourth region bears hooks between 60 and 100 μm long (Figs 48C, 49). When it is evaginated, the penis is enclosed within the penial vestibule, which may be spherical or pear-shaped depending on the orientation of the penis inside (Fig. 47B). When it is not evaginated, the penis is within the penial sheath, and the hooks are hidden within the penis (Fig. 49A–C). All specimens examined were sexually mature. The deferent duct is narrow and convoluted. The retractor muscle is long (equal to at least one third of the length of the body cavity) and inserts posteriorly by the rectum.

**Figure 50.**
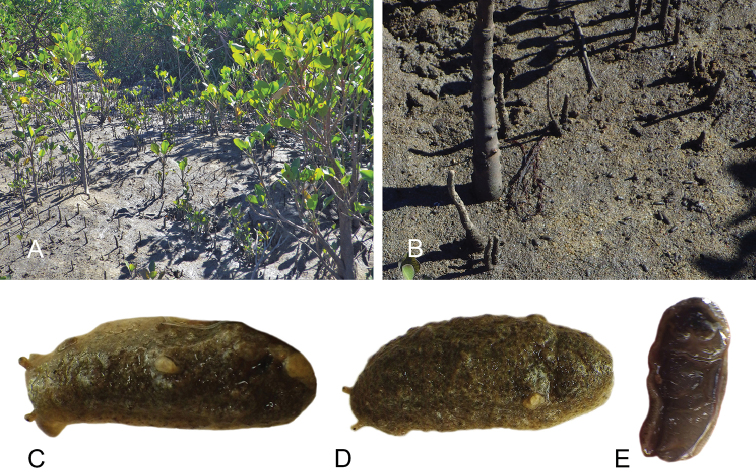
Habitats and live specimens, *Wallaconchis achleitneri*, Australia, Queensland, Bowen, Doughty Creek. **A–B** Type locality, sandy area with *Avicennia* and *Rhizophora* trees in narrow band by the creek (st 117) **C–D** Dorsal view, approximately 14 mm long (MTQ st117) **E** Ventral view, approximately 14 mm long (MTQ st 117).

##### Remarks.

*Onchidium graniferum* must be transferred to *Wallaconchis* because the diagnostic combination of characters of *Wallaconchis* is found in Semper’s original description and in what remains of the syntypes. Semper (1882: 274) wrote that there is no accessory penial gland and no rectal gland in *O. graniferum*, which is fortunate here because the copulatory organs of the syntypes are all missing. Semper did not describe the intestinal loops type but the one digestive system that remains in the type material is clearly of type I. Semper’s description of the male opening below the right tentacle also is consistent with the fact that *O. graniferum* belongs to *Wallaconchis*.

The only issue in Semper’s description (1882: 274, our translation from German) of *O. graniferum* is that he wrote that the retractor muscle of the penis inserted “somewhat behind the center of the animal,” or slightly posterior to the center of the body cavity. In all *Wallaconchis* species, the retractor muscle inserts at the posterior end of the body cavity, near the rectum. More precisely, that type of insertion is found in all mature animals. In small animals (which may or may not be fully mature), the retractor muscle occasionally inserts near the heart, approximately at mid-length of the body cavity. One of the syntypes that Semper examined was only 11 mm long and would likely be immature. If Semper observed the insertion of the retractor muscle in this immature specimen, it could explain why his description of the retractor muscle differs from the insertion observed here.

The publication dates of various sections of the volume on *Landmollusken* by Carl Semper in the *Reisen im Archipel der Philippinen* series were clarified by Johnson (1969). The species name *Onchidium graniferum* was first published by Semper in 1880 (pl. 19, fig. 13 & pl. 23, fig. 3) with two illustrations of the external anatomy and the radula and no written description. Because *Onchidium graniferum* was published before 1931, ICZN (1999) Article 12.2.7 applies, and Semper’s figures are regarded as an indication accompanying the new name *Onchidium graniferum*. The text of the description and the illustration of the radula (pl. 21, fig. 10) were published in 1882. No other existing names were found to apply to the species described here.

The copulatory organs of the syntypes were all destroyed prior to the present study (likely by Semper himself), but Semper (1882: 274, translated from German) described the penis of *O. graniferum* as a “cartilaginous tube” with only a “tooth-bearing portion” and “cartilage teeth of very different sizes.” Semper (1882: 274, our translation from German) also indicated a specific order in which penial hooks (which he called teeth) of different sizes are organized, which is in agreement with the arrangement of hooks described here: “The foremost ones are very irregular and broad; they are on average 80 μm long. Then a section with teeth three times as long, of 180 μm, then again another with small ones; but in the last part great ones are found. The large teeth are very tightly pressed.” Semper illustrated these penial hooks of different sizes individually, but not the hooks together *in situ*, so the exact arrangement of the penial hooks that he observed cannot be known with certainty. Semper also did not indicate whether the penis was evaginated or not, but it can be assumed that his “foremost” hooks are at the base (i.e., proximal on the penis when evaginated). So, overall, Semper’s description of the arrangement of penial hooks of different sizes is consistent with the pattern of penial hooks observed here from the proximal base of the penis to its evaginated tip. Semper’s illustrations of a thick penial sheath and the shapes of the penial hooks are also consistent with the species described here. Finally, Semper described the penis as slightly shorter than the retractor muscle. The retractor muscle was found to be shorter than the penis in the species described here, but this trait varies within *Wallaconchis* species and is not useful for identification.

Britton (1984: fig. 4D) illustrated the penial anatomy of an onchidiid species from Hong Kong which matches the penial anatomy of the species described here perfectly. Britton misidentified that species as *Paraoncidium reevesii* (J. E. Gray, 1850) because the valid name *Onchidium reevesii* J. E. Gray, 1850 actually applies to a species with both an accessory penial gland and a rectal gland, both of which Britton noted were absent from his Hong Kong specimens (Dayrat et al. 2016). Britton’s description (1984: 184) of the intestinal loops of type II is unlikely given the distinctive penial anatomy he illustrated (known only in this onchidiid species). Britton either made an error characterizing the intestinal loops, or the specimens he examined were a mix of multiple species with different intestinal types. Britton’s record of *Paraoncidium reevesii* is thought to be a misidentification for *Wallaconchis graniferum*. Finally, the presence of *Wallaconchis graniferum* in China is also known thanks to a few DNA sequences found in GenBank, misidentified as *Onchidella* sp. (Sun et al. 2014) and that are part of the species described here (Fig. 1).

Labbé (1934) identified a specimen from the Philippines as *Paraoncidium graniferum* for which he mentioned intestinal loops of type I, no rectal gland, and no accessory penial gland, which together, if correct, indicate that he examined a *Wallaconchis* species. Labbé’s description (1934: 228) of the penis as “curled, with teeth anteriorly” suggests that he probably examined *W. graniferum*, but the description of the hooks is not detailed enough to be sure that he did not examine *W. uncinus* or another species. At any rate, this species is found in the Philippines and Labbé’s record would not improve our knowledge of their geographic distribution.

Finally, Hoffmann (1928: 80–81, our translation from German) commented that Semper’s description was inconsistent: “the cartilage of the penis is approximately 3 mm long and has only the tooth-bearing portion” but in “his illustration, which is made in natural size, the penis is 7 mm long.” However, contrary to Hoffmann’s claim, Semper’s description is not inconsistent. Indeed, it is the entire penial sheath which is 7 mm long in Semper’s illustration and not the penis itself.

#### Wallaconchis achleitneri

Taxon classificationAnimaliaSystellommatophoraOnchidiidae

Goulding sp. n.

http://zoobank.org/91D9C26C-2483-4330-BBA2-2196C5B91CA2

**Figure 51.**
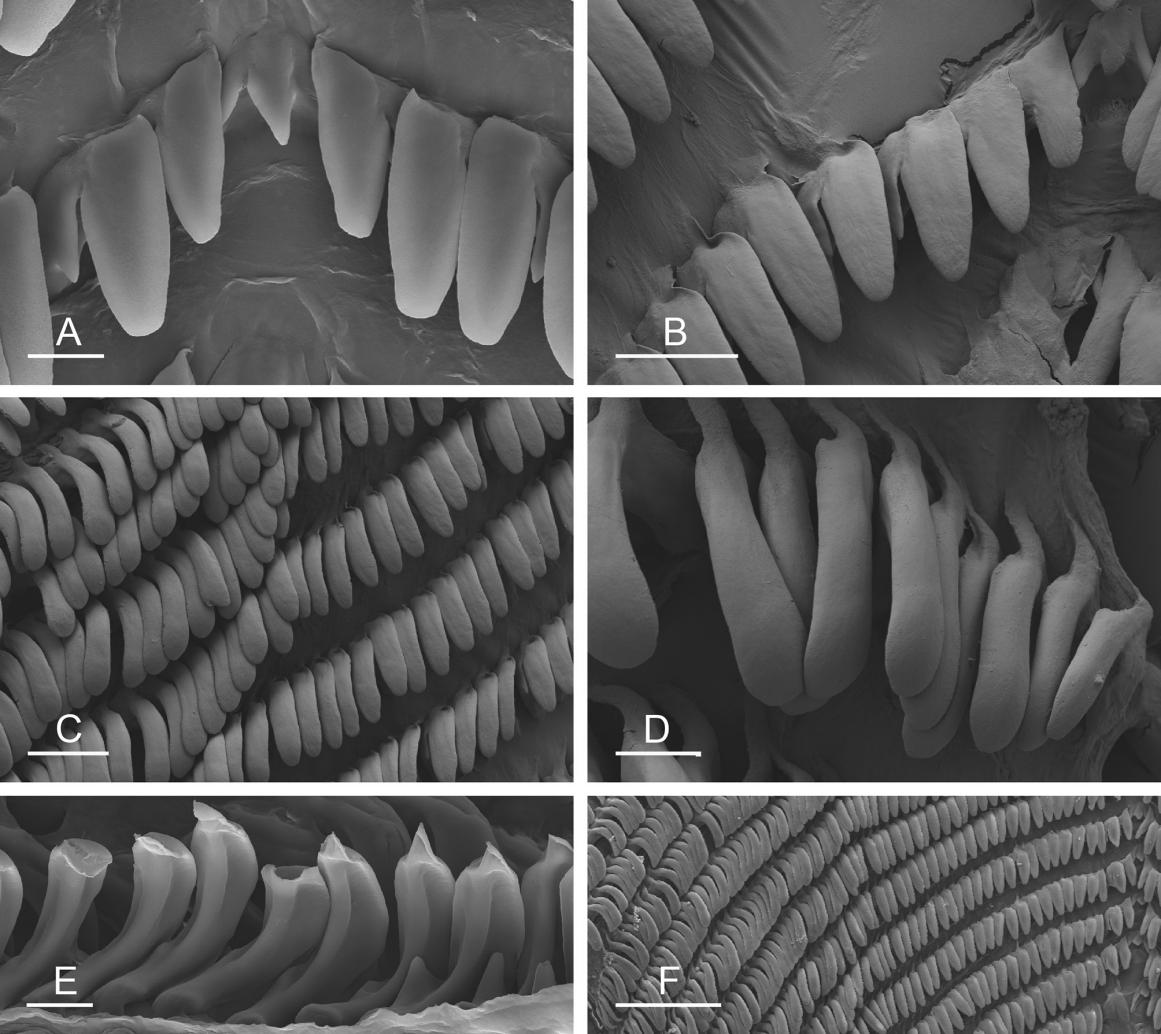
Radula, *Wallaconchis achleitneri*, Australia, Queensland (MTQ st117). **A** Rachidian and innermost lateral teeth, scale bar 6 μm [#1] **B** Rachidian and innermost lateral teeth, holotype, scale bar 20 μm [3534] **C** Transition between inner lateral teeth and outer lateral teeth, holotype, scale bar 30 μm [3534] **D** Outermost lateral teeth, holotype, scale bar 10 μm [3534] **E** Lateral teeth with basal lateral spine decreasing in size from right to left, scale bar 10 μm [#1] **F** Left lateral teeth, scale bar 100 μm [#2].

**Figure 52.**
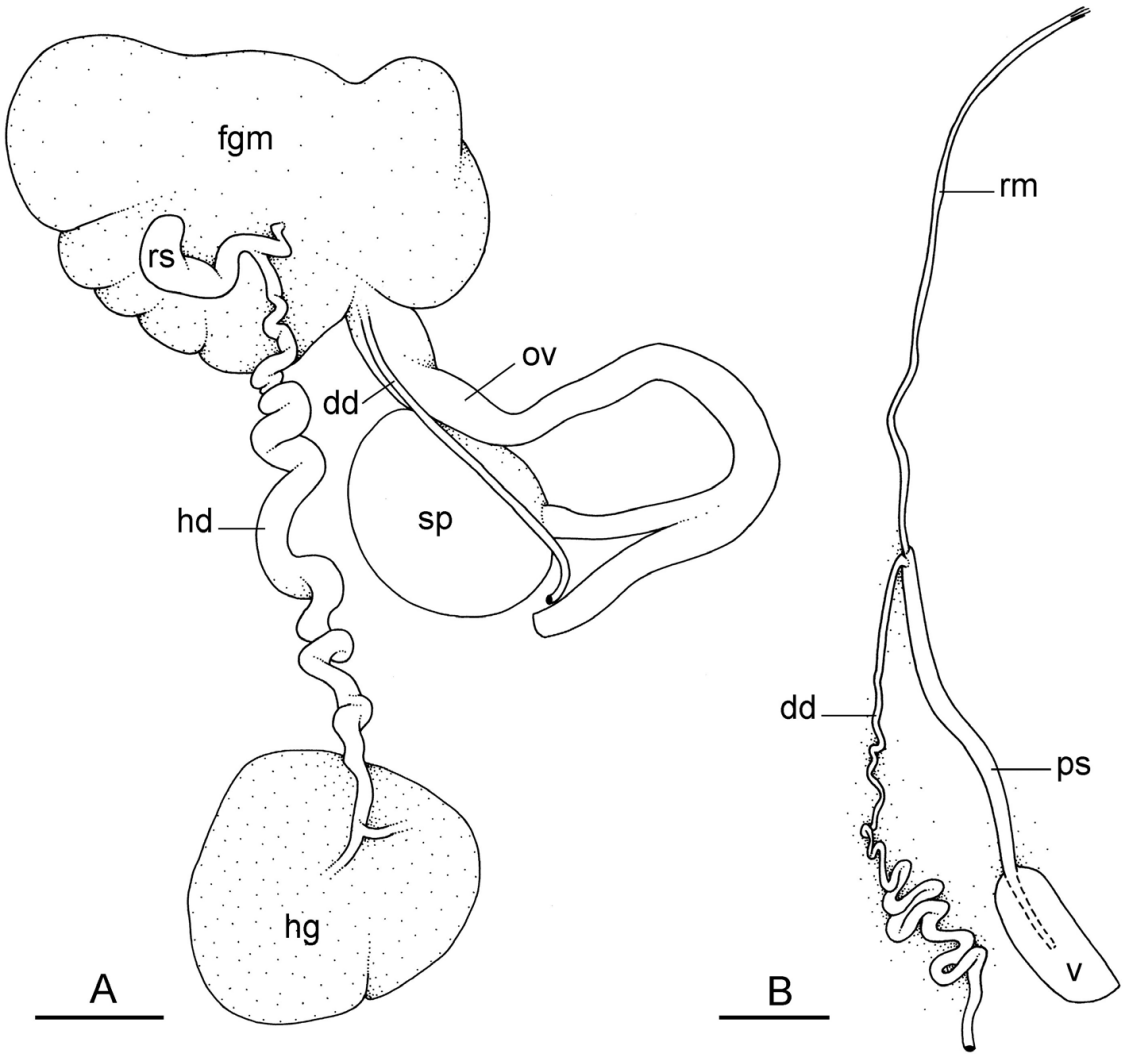
Reproductive system, *Wallaconchis achleitneri*, Australia, Queensland (MTQ st117). **A** Hermaphroditic (female), posterior parts, scale bar 1 mm [#4] **B** Anterior, male copulatory parts, scale bar 1 mm [#1]. Abbreviations: **dd** deferent duct **fgm** female gland mass **hd** hermaphroditic duct **hg** hermaphroditic gland **ov** oviduct **ps** penial sheath **rm** retractor muscle **rs** receptaculum seminis **sp** spermatheca **v** vestibule.

**Figure 53.**
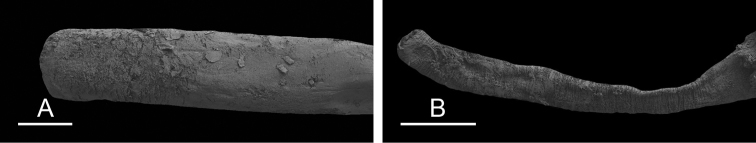
Penis, *Wallaconchis achleitneri*, Australia, Queensland (MTQ st117). **A** Scale bar 100 μm [2645] **B** Holotype, scale bar 100 μm [3534].

Figs 50 , 51 , 52 , 53

##### Type locality.

Australia, Queensland, Bowen, Doughty Creek, 20°01.26'S, 148°14.35'E, st 117, *Avicennia* and *Rhizophora* trees by a creek with sandy mud.

##### Type material.

Holotype, 7/5 mm [3534], designated here (st 117, MTQ).

##### Additional material examined.

**Australia**, Queensland, Bowen, Doughty Creek, 20°01.26'S, 148°14.35'E, 64 specimens 16/10 mm [#3] to 8/5 mm [3535]; 14/8 mm [#1], 14/8 mm [#2], 14/8 mm [#4] and 9/6 mm [2645], st 117, *Avicennia* and *Rhizophora* trees by a creek with sandy mud (st 117, MTQ).

##### Distribution.

Australia: Queensland (type locality).

##### Habitat

(Fig. 50A–B, Table 3). Animals were found abundantly in a small mangrove patch near a small river, on mud with large grain sand. *Wallaconchis achleitneri* was found at only one station in Queensland despite five weeks of sampling, suggesting it may require something particular in this habitat, possibly the sediment.

##### Etymology.

*Wallaconchis achleitneri* is dedicated to Stefan Achleitner, a team member in our expedition to Queensland, and whose German translation helped to clarify parts of old and challenging species descriptions.

##### Diagnosis

(Table 5). Externally, *Wallaconchis achleitneri* cannot be distinguished from brown or grey specimens of other *Wallaconchis* species, except by its small size. Internally, the combination of a smooth, long, and narrow penis within a large penial vestibule, a spherical spermatheca, a free (not attached to the body wall by fibers), and a slightly enlarged oviduct distinguishes *W. achleitneri* from other *Wallaconchis* species. Finally, even though its known species distribution may change in the future, *W. achleitneri* is not presently considered to be sympatric with any other *Wallaconchis* species.

##### Color and morphology of live animals

(Fig. 50C–E). The dorsal notum is brownish grey. The ocular tentacles are orange-brown. The hyponotum and foot are yellow-grey.

##### External morphology.

The number of dorsal papillae with eyes is three to four papillae with three to four eyes per papilla. There is a retractable papilla with three to four eyes in the center of the dorsal notum, but which is not raised above the other papillae.

##### Digestive system

(Fig. 51, Table 4). Examples of radular formulae are presented in Table 4. The length of the rachidian teeth is approximately 15 µm, significantly smaller than that of the lateral teeth. The length of the hook of the lateral teeth gradually increases (from 30 to 35 µm) from the inner to the outer teeth (excluding the innermost and outermost lateral teeth which are significantly smaller). The intestinal loops are of type I.

##### Reproductive system

(Fig. 52A). In the posterior (hermaphroditic) part of the reproductive system, the oviduct is larger in circumference than the deferent duct. The spherical spermatheca joins the distal region of the oviduct through a short duct.

##### Copulatory apparatus

(Figs 52B, 53). The penis (approximately 0.6–0.9 mm long) is narrow and smooth with no hooks (Fig. 53). The penial vestibule is cylindrical and the penial sheath is narrow. The distal end of the penis lies within the vestibule (Fig. 52B). The distal end of the deferent duct is approximately the same width as the penial sheath. The deferent duct is convoluted. The retractor muscle is slightly longer than the penial sheath and inserts at the posterior end of the body cavity, near the rectum.

#### Wallaconchis comendadori

Taxon classificationAnimaliaSystellommatophoraOnchidiidae

Goulding & Dayrat sp. n.

http://zoobank.org/F96331E4-4A09-425E-B103-62811C10816D

**Figure 54.**
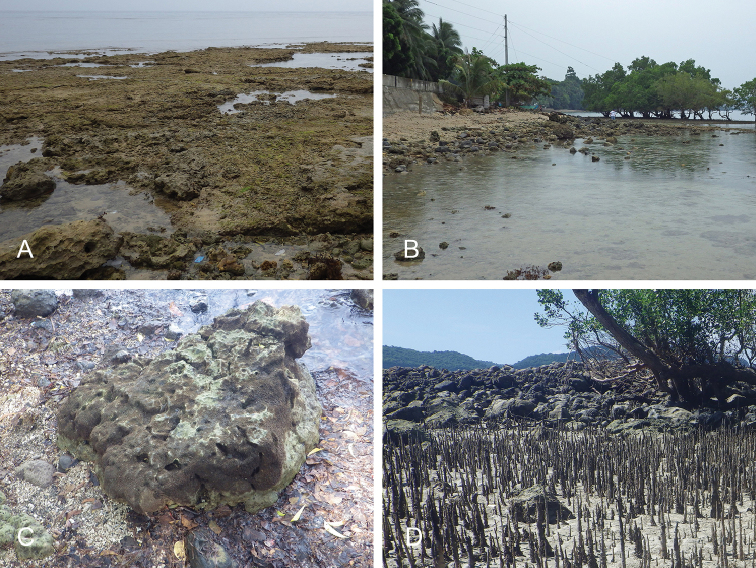
Habitats, *Wallaconchis comendadori*. **A** Type locality, Philippines, Bohol, Guindulman, rocks and coral rubble near a few *Avicennia* trees (st 197) **B** View of the shore (same locality as A) **C** Indonesia, Bali, Pemuteran, Labuhan Lalang Harbor, coral rubble, rocks and a few *Avicennia* trees (st 157) **D** Indonesia, Lombok, Don Don, old *Avicennia* forest, with coral rubble (st 149).

**Figure 55.**
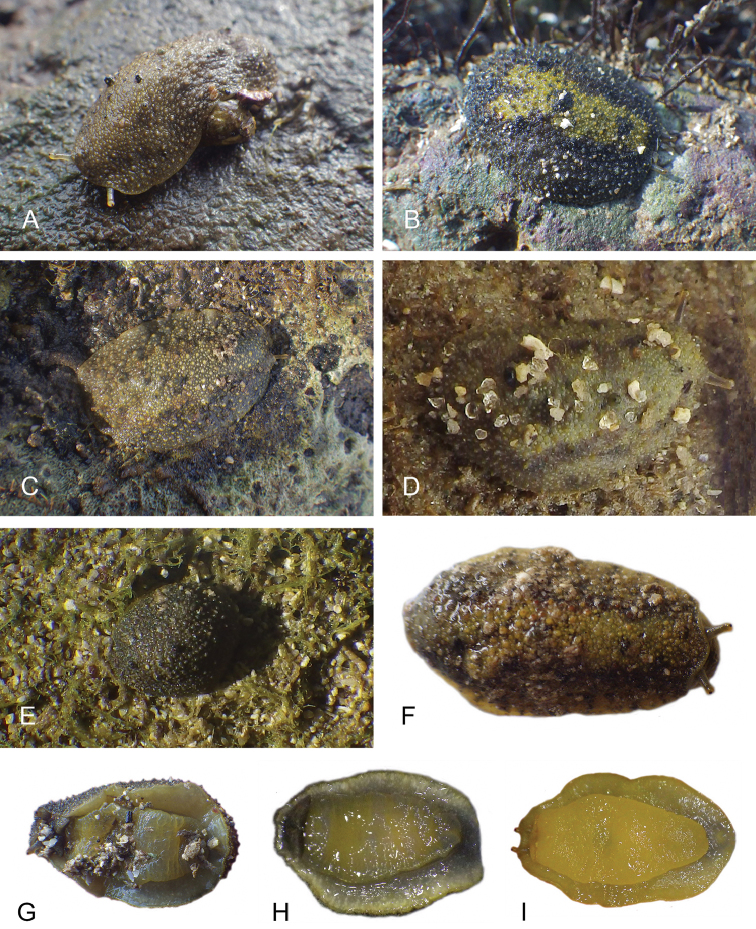
Live specimens, *Wallaconchis comendadori*. **A** Dorsal view, 16 mm long [3133], Indonesia, Bali (UMIZ 00064) **B** Dorsal view, 18 mm long [2985], Indonesia, Lombok (UMIZ 00063) **C** Dorsal view, 13 mm long [3131], Indonesia, Bali (UMIZ 00064) **D** Dorsal view, 12 mm long [2725], Indonesia, Ambon (UMIZ 00061) **E** Dorsal view, 7 mm long [2983], Indonesia, Lombok (UMIZ 00063) **F** Dorsal view, holotype, 12 mm long [3626], Philippines, Bohol (PNM 041232) **G** Ventral view, same as B **H** Ventral view, 15 mm long [2315], Indonesia, Sulawesi (UMIZ 00060) **I** Ventral view, 22 mm long [3385], Philippines, Bohol (PNM 041234).

**Figure 56.**
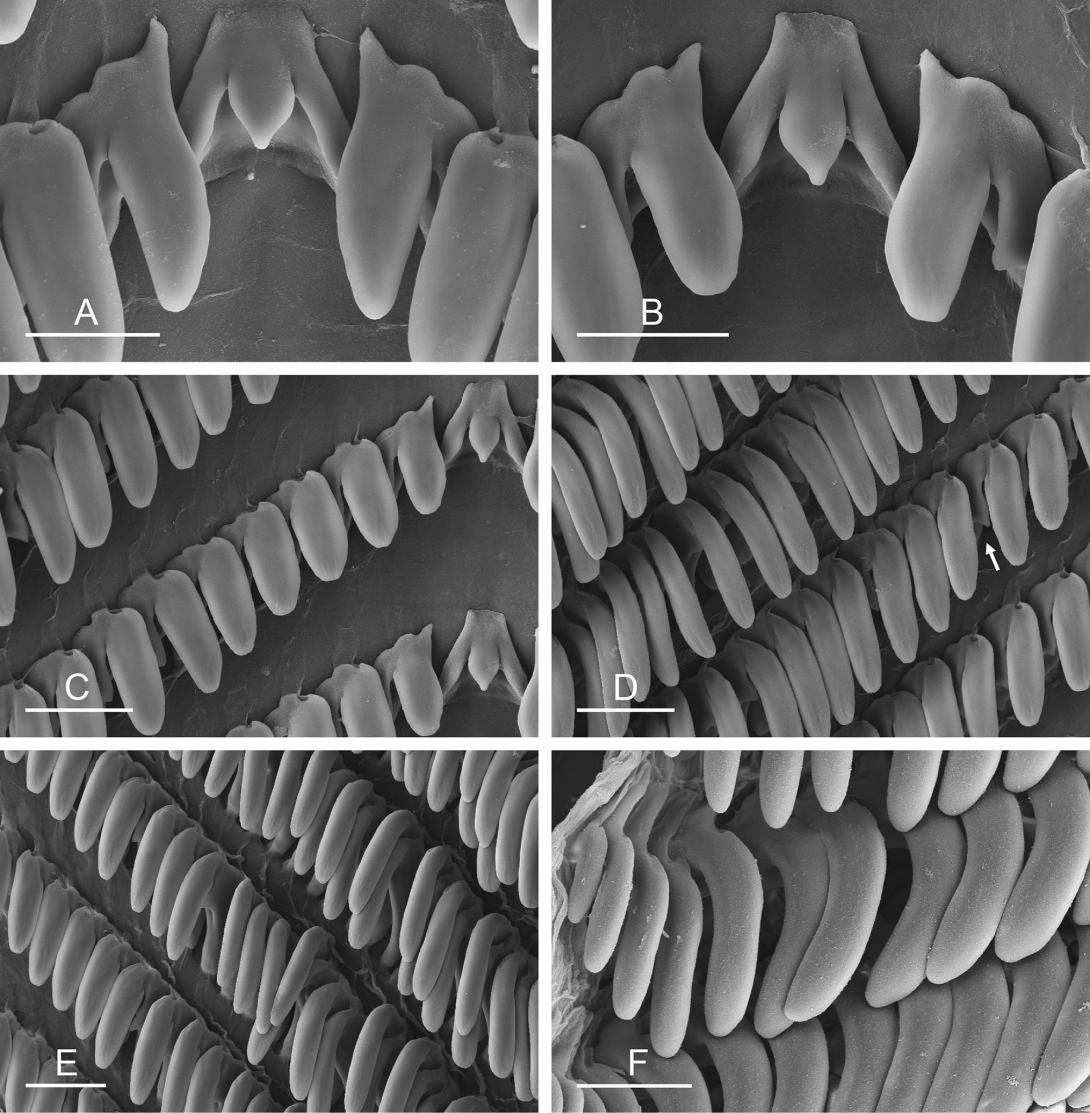
Radula, *Wallaconchis comendadori*. **A** Rachidian and innermost lateral teeth, Philippines, Bohol, scale bar 20 μm [3385] (PNM 041234) **B** Rachidian and innermost lateral teeth, Indonesia, Lombok, scale bar 20 μm [2985] (UMIZ 00063) **C** Rachidian and inner lateral teeth, scale bar 30 μm, same as B **D** Transition between inner lateral teeth and outer lateral teeth, arrow shows basal lateral spine, scale bar 30 μm, same as B **E** Transition between inner lateral teeth and outer lateral teeth, scale bar 30 μm, same as B **F** Outermost lateral teeth, Indonesia, Ambon, scale bar 20 μm [3539] (UMIZ 00061).

**Figure 57.**
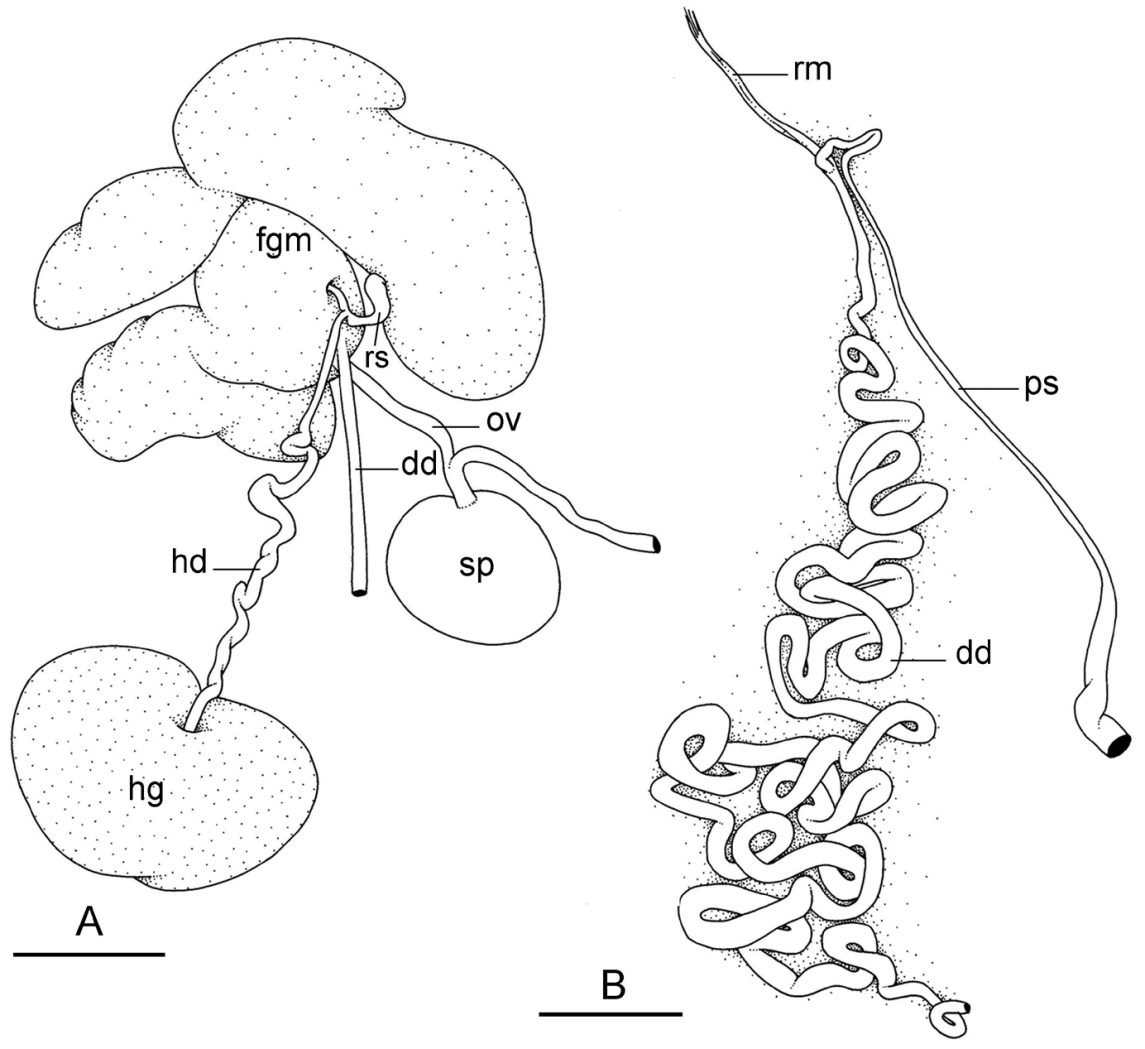
Reproductive system, *Wallaconchis comendadori*. **A** Hermaphroditic (female), posterior parts, Indonesia, Lombok, scale bar 3 mm [2985] (UMIZ 00063) **B** Anterior, copulatory parts, Philippines, Bohol, scale bar 2 mm [3385] (PNM 041234). Abbreviations: **dd** deferent duct **fgm** female gland mass **hd** hermaphroditic duct **hg** hermaphroditic gland **ov** oviduct **ps** penial sheath **rm** retractor muscle **rs** receptaculum seminis **sp** spermatheca.

**Figure 58.**
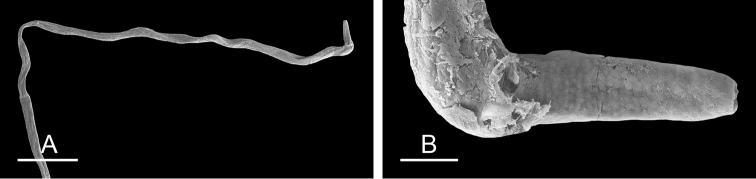
Penis, *Wallaconchis comendadori*, Philippines, Bohol [3385] (PNM 041234). **A** View of entire penis, scale bar 0.5 mm **B** Distal tip of penis, scale bar 50 μm.

Figs 54 , 55 , 56 , 57 , 58

##### Type locality.

Philippines, Bohol, Guindulman, 09°44.06'N, 124°27.63'E, st 197, rocks and coral rubble near a few *Avicennia* trees.

##### Type material.

Holotype: 12/11 mm [3626], designated here (PNM 041232).

##### Additional material examined.

**Indonesia**, Sulawesi, Mantehage Island, 01°41.88'N, 124°46.74'E, 1 specimen 15/7 mm [2315], st 91, rocks behind a mangrove of *Sonneratia* and *Rhizophora* (UMIZ 00060); Ambon, Haruku Island, 03°36.52'S, 128°25.07'E, 2 specimens 12/7 mm [2725] and 7/6 mm [3539], st 127, rocky *Sonneratia* mangrove with coral rubble (UMIZ 00061); Lombok, Don Don, 08°54.54'S, 116°21.50'E, 2 specimens 18/12 mm [2985] and 7/6 mm [2983], st 149, old *Avicennia* forest with coral rubble (UMIZ 00063); Bali, Pemuteran, Labuhan Lalang Harbor, 08°08.61'S, 114°32.33'E, 2 specimens 16/8 mm [3133] and 13/9 mm [3131], st 157, coral rubble, rocks and a few *Avicennia* (UMIZ 00064). **Philippines**, Bohol, Guindulman, 09°44.06'N, 124°27.63'E, 2 specimens 30/11 mm [3627] and 19/15 mm [3630], st 197, rocks and coral rubble near a few *Avicennia* trees (PNM 041233); Bohol, Maribojoc, 09°44.020'N, 123°47.45'E, 2 specimens 22/13 mm [3385] and 21/16 mm [3400], st 200, coral rubble with sand, low tide at night (PNM 041234).

##### Distribution.

Indonesia: Ambon, Bali, Lombok, and Sulawesi. Philippines: Bohol.

##### Habitat

(Fig. 54, Table 3). *Wallaconchis comendadori* is found in the rocky intertidal, on rocks or large pieces of coral rubble, generally covered by a thin mat of algae.

##### Etymology.

This species is dedicated to Joseph Comendador, from the National Museum of the Philippines. Our expedition in the Philippines would not have been possible without his help with logistics, and we enjoyed exploring mangroves with him.

##### Diagnosis

(Table 5). Externally, *Wallaconchis comendadori* cannot be distinguished from brown or black specimens of other *Wallaconchis* species, although a bright yellow, longitudinal band on the dorsum is more commonly found in this species. Internally, the narrow penis, a free oviduct (not attached to the body wall by fibers), and the apple-shaped spermatheca distinguishes *W. comendadori* from most *Wallaconchis* species, except *W. melanesiensis*, from which it differs by having a longer penis and a more highly convoluted deferent duct.

##### Color and morphology of live animals

(Fig. 55). Animals are not frequently brightly colored. The dorsal notum is predominantly brown or black, which may be mixed with subtle patches of yellow, green, red, and grey. A median, longitudinal yellow band was observed in a few individuals. The ocular tentacles are brown or grey. The hyponotum is light or bright yellow. The foot is bright yellow or light yellow-grey.

##### External morphology.

Between five and seven papillae bear eyes (with three or four eyes per papilla) but more may be retracted. There is a retractable papilla with eyes in the center of the dorsal notum, which is not raised above the other papillae.

##### Digestive system

(Fig. 56, Table 4). Examples of radular formulae are presented in Table 4. The length of the rachidian teeth is approximately 15–22 µm, significantly smaller than that of the lateral teeth. The length of the lateral teeth (from 30 to 60 µm) gradually increases from the inner to the outer teeth (excluding the innermost and outermost lateral teeth which are significantly smaller). The intestinal loops are of type I.

##### Reproductive system

(Fig. 57A). The oviduct is narrow, approximately the same width as the deferent duct. The spermatheca is apple-shaped, with two lobes, and joins the oviduct through a short duct.

##### Copulatory apparatus

(Figs 57B, 58). The penis (more than 1.5 mm long) is narrow (approximately 100 µm) and smooth with no hooks (Fig. 58). The penial sheath is narrow proximally, and gradually widens distally into a vestibule. The penis is within the narrow region of the vestibule. The deferent duct is extremely convoluted (Fig. 57B), but slightly less convoluted in immature specimens. The length of the penial sheath equals approximately two thirds to three quarters the length of the body cavity. The deferent duct is approximately the same width as the penial vestibule. The retractor muscle is narrow and inserts at the posterior end of the body cavity, near the rectum.

#### Wallaconchis melanesiensis

Taxon classificationAnimaliaSystellommatophoraOnchidiidae

Goulding & Dayrat sp. n.

http://zoobank.org/31E8A78F-1DF3-4B41-A6EB-F0E5F515299A

**Figure 59.**
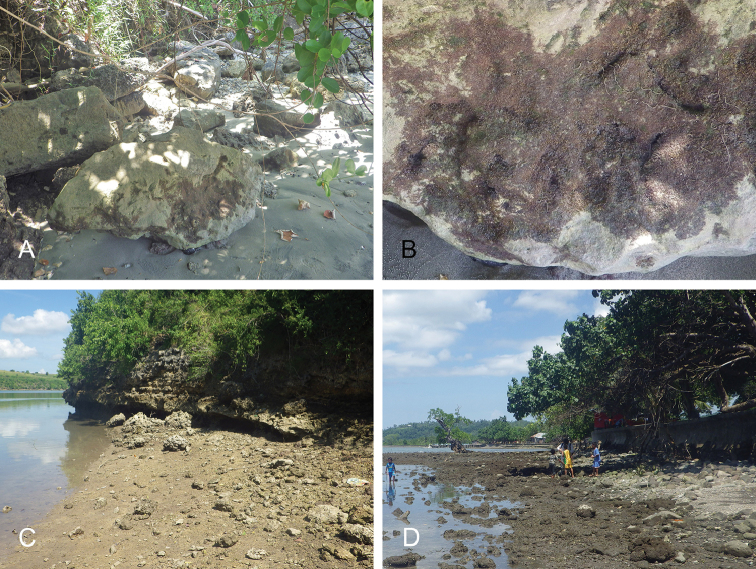
Habitats, *Wallaconchis melanesiensis*, Indonesia. **A** Halmahera, Foli, large rocks with algae high in intertidal (st 217) **B** Close-up of boulder showing the algae where the onchidiid was found (same locality as A) **C** Lombok, Seriwe Bay, narrow rocky beach between a cliff and the sea water **D** Halmahera, Sofifi, muddy, rocky intertidal (st 204).

**Figure 60.**
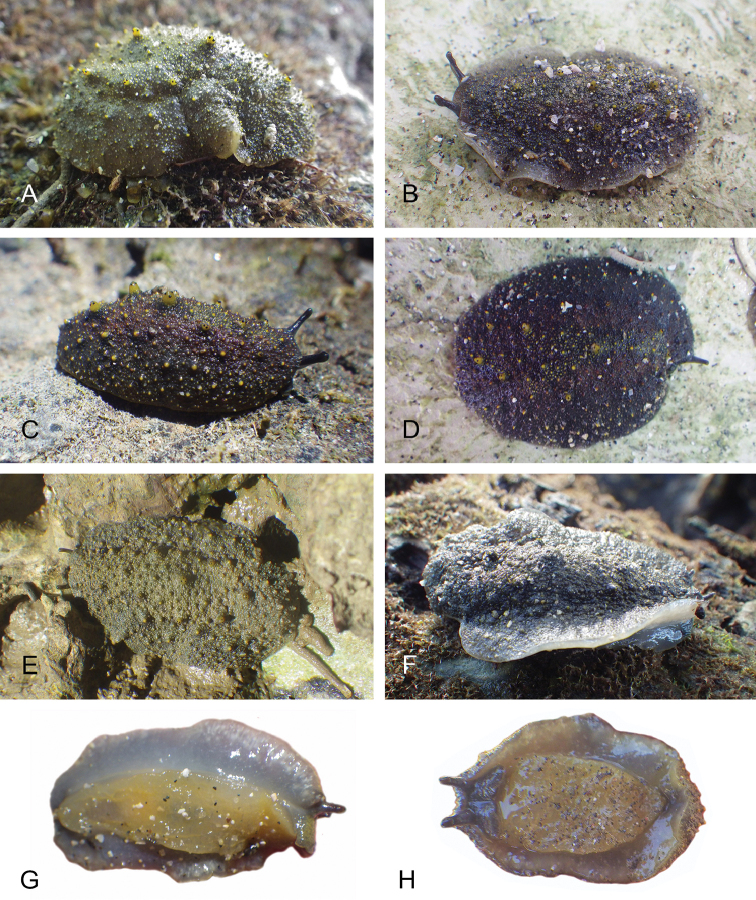
Live specimens, *Wallaconchis melanesiensis*, Indonesia. **A** Dorsal view, 25 mm long [5131], Halmahera (UMIZ 00069) **B** Dorsal view, 27 mm long [5133], Halmahera (UMIZ 00069) **C** Holotype, dorsal view, 15 mm long [5065], Halmahera (UMIZ 00065) **D** Dorsal view, 23 mm long [5132], Halmahera (UMIZ 00069) **E** Dorsal view, 27 mm long [2963], Lombok (UMIZ 00070) **F** Dorsal view, 43 mm long [2735], Ambon (UMIZ 00068) **G** Ventral view, same as B **H** Ventral view, same as C.

**Figure 61.**
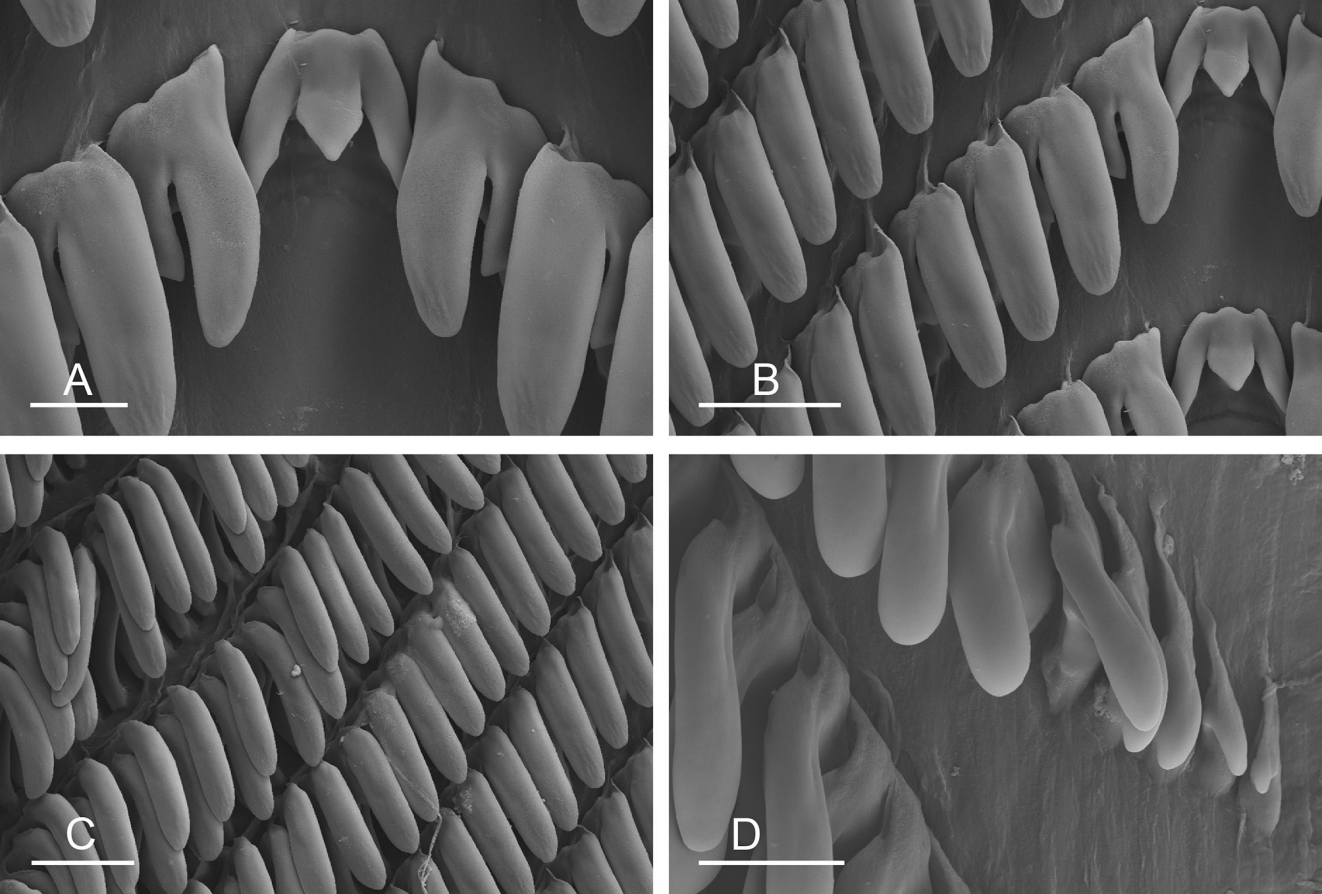
Radula, *Wallaconchis melanesiensis*, Indonesia (**A–C**) Halmahera [5132] (UMIZ 00069) (D) Ambon [2733] (UMIZ 00068). **A** Rachidian and innermost lateral teeth, scale bar 20 μm **B** Rachidian and inner left lateral teeth, scale bar 20 μm **C** Transition between inner lateral teeth and outer lateral teeth, scale bar 30 μm **D** Outermost lateral teeth, scale bar 20 μm.

**Figure 62.**
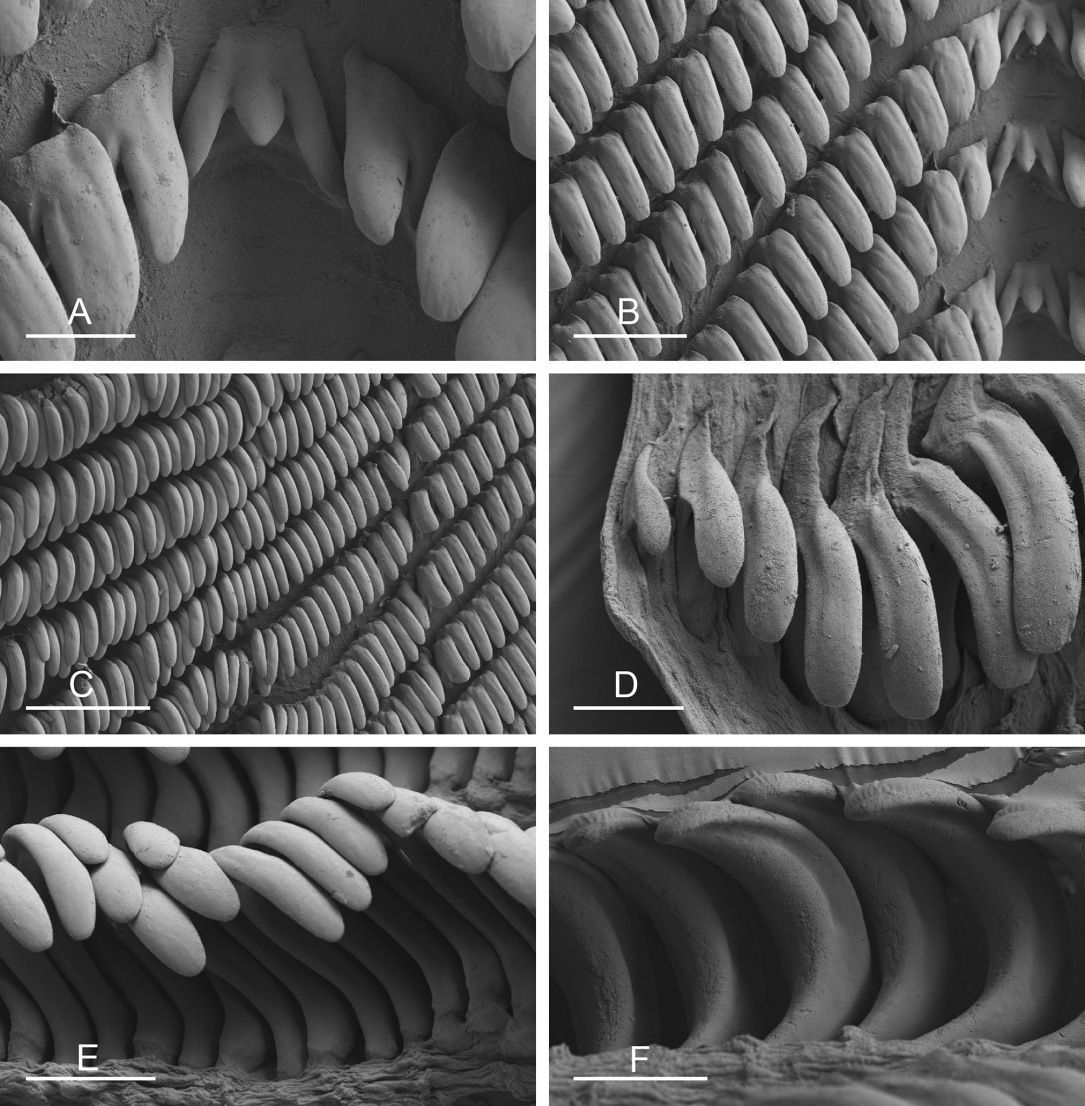
Radula, *Wallaconchis melanesiensis*, Vanuatu [5484] (IM-2013-62406). **A** Rachidian and innermost lateral teeth, scale bar 20 μm **B** Rachidian and inner left lateral teeth, scale bar 50 μm **C** Transition between inner lateral teeth and outer lateral teeth, scale bar 100 μm **D** Outermost lateral teeth, scale bar 20 μm **E** Hook of left lateral teeth from underneath, without basal lateral spine, scale bar 30 μm **F** Hook of left lateral teeth from underneath, without basal lateral spine, scale bar 20 μm.

**Figure 63.**
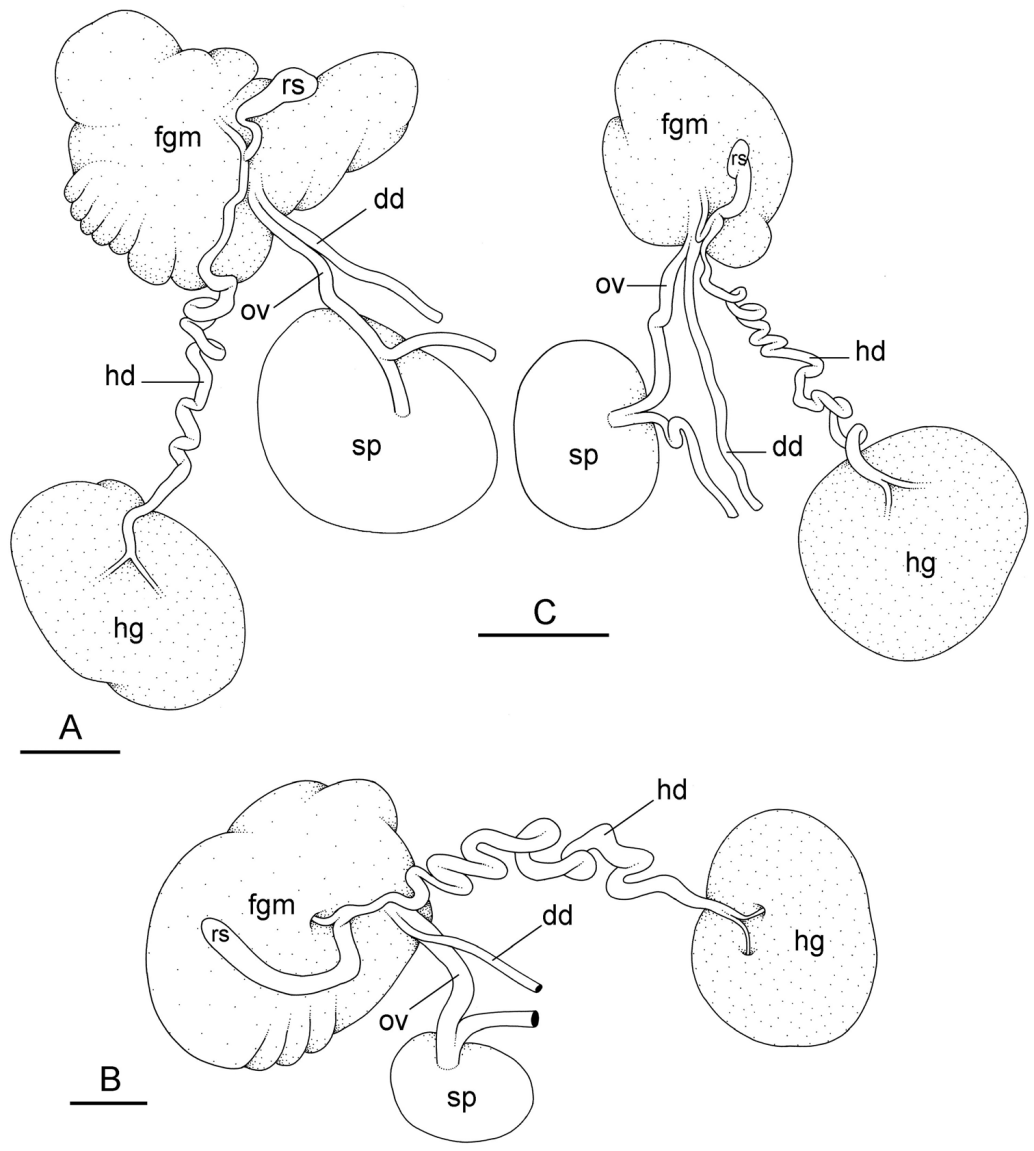
Reproductive system, hermaphroditic (female), posterior parts, *Wallaconchis melanesiensis.* **A** Indonesia, Halmahera, scale bar 2 mm [5131] (UMIZ 00069) **B** Vanuatu, scale bar 1 mm [5484] (IM-2013-62406) **C** Indonesia, Lombok, scale bar 2.4 mm [2963] (UMIZ 00070). Abbreviations: **dd** deferent duct **fgm** female gland mass **hd** hermaphroditic duct **hg** hermaphroditic gland **ov** oviduct **rs** receptaculum seminis **sp** spermatheca.

**Figure 64.**
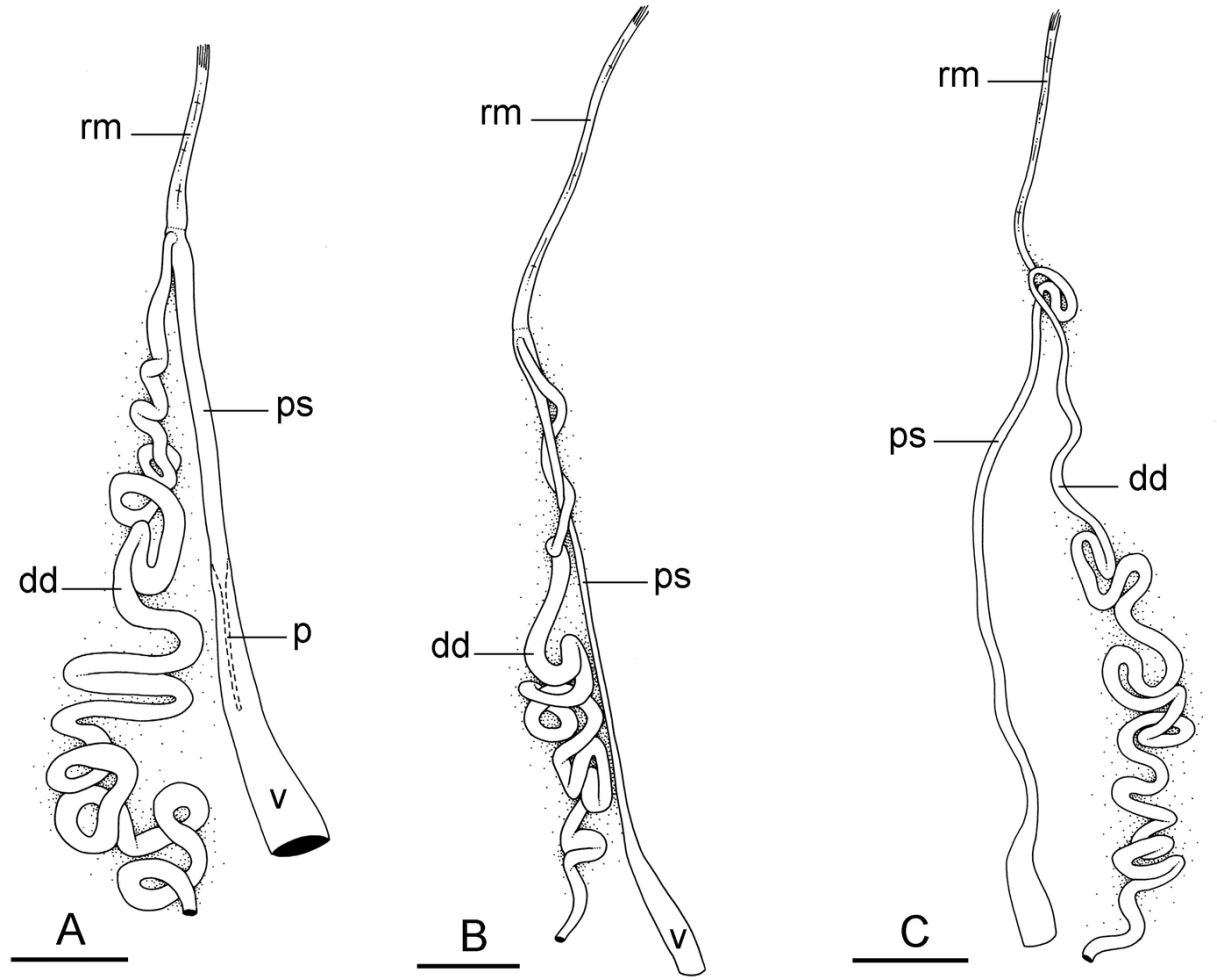
Reproductive system, anterior, male copulatory parts, *Wallaconchis melanesiensis*. **A** Vanuatu, scale bar 1 mm [5484] (IM-2013-62406) **B** Indonesia, Halmahera, scale bar 2 mm [5132] (UMIZ 00069) **C** Indonesia, Lombok, scale bar 2 mm [2963] (UMIZ 00070). Abbreviations: **dd** deferent duct **p** penis **ps** penial sheath **rm** retractor muscle **v** vestibule.

**Figure 65.**
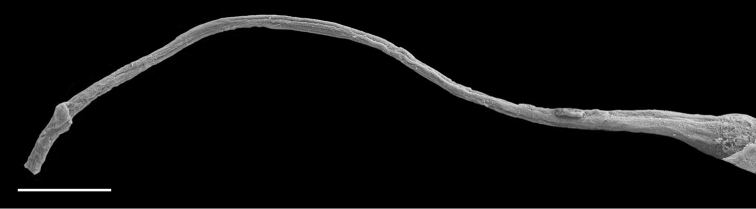
Penis, *Wallaconchis melanesiensis*, Papua New Guinea, Madang, scale bar 10 μm [5421] (IM-2013-14039).

Figs 59 , 60 , 61 , 62 , 63 , 64 , 65

##### Type locality.

Papua New Guinea, Madang, SW Hargun Island, 05°01.60'S, 145°47.90'E, st PM24, night tide.

##### Type material.

Holotype, 13/10 mm [5417], designated here (IM-2013-13761).

##### Additional material examined.

**Indonesia**, Lombok, Seriwe Bay, 08°51.70'S, 116°32.87'E, 1 specimen 27/14 mm [2963], st 147, small beach of coral rubble and rocks (UMIZ 00070); Sulawesi, Tamperong, 01°41.51'N, 125°00.80'E, 1 specimen 8/7 mm [2202], st 85, sand and small rocks outside a mangrove (UMIZ 00066); Sulawesi, Bahoi, 01°43.36'N, 125°01.23'E, 1 specimen 26/17 mm [2215], st 88, sand and small rocks outside a mangrove (UMIZ 00067); Ambon, Haruku Island, 03°36.52'S, 128°25.07'E, 3 specimens 31/25 mm [2735], 31/25 mm [2732], and 13/8 mm [2733], st 127, rocky *Sonneratia* mangrove with coral rubble (UMIZ 00068); Halmahera, Sofifi, 00°45.40'N, 127°35.47'E, 1 specimen 15/8 mm [5065], st 204, muddy, rocky intertidal (UMIZ 00065); Halmahera, Foli, 01°14.66'N, 128°10.61'E, 4 specimens 27/17 mm [5133], 25/19 mm [5131], 23/18 mm [5132], and 22/13 mm [5026], st 217, large rocks with algae high in intertidal of beach (UMIZ 00069). **Papua New Guinea**, Madang, SW Hargun Island, 05°01.60'S, 145°47.90'E, 1 specimen 17/14 mm [5421], st PM24, night tide (IM-2013-14039); Madang, SW Hargun Island, 05°01.60'S, 145°47.90'E, 1 specimen 7/6 mm [5446], st PM24, night tide (IM-2013-14046); New Ireland, Kavieng, 02°41.00'S, 150°57.00'E, 1 specimen 19/18 mm [6089], st KM05, mixed hard platform and seagrass bed at outlet of rivulet (IM-2013-53524); New Ireland, Kavieng, 02°41.00'S, 150°57.00'E, 1 specimen 20/15 mm [6090], st KM05, mixed hard platform and seagrass bed at outlet of rivulet (IM-2013-53522). **Vanuatu**, Santo Rose Point, 15°34.90'S, 167°02.40'E, 1 specimen 14/13 mm [5483], st VM02, intertidal, coral sand (IM-2013-62405); Santo Rose Point, 15°34.90'S, 167°02.40'E, 1 specimen 14/12 mm [5484], st VM02, intertidal, coral sand (IM-2013-62406).

##### Distribution.

Indonesia: Ambon, Halmahera, and Sulawesi. Papua New Guinea: Madang and Kavieng. Vanuatu.

##### Habitat

(Fig. 59, Table 3). *Wallaconchis melanesiensis* is found in the rocky intertidal, on rocks or large pieces of coral rubble generally covered by a thin mat of algae.

##### Etymology.

*Wallaconchis melanesiensis* is named after the region of Melanesia, as it is the only *Wallaconchis* species found in Papua New Guinea and Vanuatu.

##### Diagnosis

(Table 5). Externally, *Wallaconchis melanesiensis* cannot be distinguished from grey or black specimens of other *Wallaconchis* species. Internally, the combination of a narrow penis, an apple-shaped spermatheca, and a free oviduct (not attached to the body wall by fibers) distinguishes *W. melanesiensis* from all *Wallaconchis* species except *W. comendadori*, from which it differs by a shorter penis and a less convoluted deferent duct (in the anterior copulatory apparatus).

##### Color and morphology of live animals

(Fig. 60). The dorsal notum is generally grey, but may be blackish red. The ocular tentacles are dark grey. The hyponotum is light grey. The foot is yellow-orange.

##### External morphology.

Between eight and ten papillae bear dorsal eyes (three or four per papilla). There is a retractable papilla with eyes in the center of the dorsal notum, which is not raised above the other papillae.

##### Digestive system

(Figs 61–62, Table 4). Examples of radular formulae are presented in Table 4. The length of the rachidian teeth is approximately 20 µm, significantly smaller than that of the lateral teeth. The length of the hook of the lateral teeth gradually increases (from 30 to 50 µm) from the inner to the outer teeth (excluding the innermost and outermost lateral teeth which are significantly smaller). The intestinal loops are of type I.

##### Reproductive system

(Fig. 63). The oviduct is narrow (approximately the same width as the deferent duct). The spermatheca is apple-shaped, with two lobes, and joins the oviduct through a short duct.

##### Copulatory apparatus

(Figs 64–65). The penis (from 0.5 to 1 mm long) is extremely narrow (approximately 20–30 µm) and smooth with no hooks (Fig. 65). The penial sheath is narrow proximally and widens distally into a vestibule (of which the shape varies) (Fig. 64). The penis is within the proximal region of the vestibule, i.e., near the end of the penial sheath (Fig. 64A). The deferent duct is highly convoluted. The length of the penial sheath is approximately two thirds of the body cavity. The deferent duct is thicker than the penial sheath (excluding the vestibule). The retractor muscle is narrow and inserts at the posterior end of the body cavity, near the rectum.

##### Remarks.

*Wallaconchis melanesiensis* is the only *Wallaconchis* species found in New Ireland (Papua New Guinea), which is the type locality of *Onchidium granulosum* Lesson, 1826. Lesson’s (1826: pl. 14, fig. 2) illustration of the dorsal notum and his written description of small, dorsal tubercles (Lesson 1830) are similar to the granular dorsal notum of *Wallaconchis*. Hoffmann (1928:86) considered *Onchidium granulosum* to be part of the genus Oncis (Platevindex) and *Oncis lata* to be a synonym of *O. granulosum*. However, *Onchidium granulosum* does not belong to *Platevindex* because the foot originally illustrated by Lesson (1826: pl. 14, fig. 2B) is much wider than the very narrow foot of *Platevindex* species. However, while the width of the foot indicates it does not belong to *Platevindex*, there is not enough information to confirm it is a *Wallaconchis* species. Because Lesson did not describe or illustrate the internal anatomy and because the type material is lost, *Onchidium granulosum* is regarded here as a *nomen dubium*.

The application of *Onchidium cinereum* Quoy & Gaimard, 1832 (with a type locality in Tonga) has remained confusing. The original description is short and uninformative. The type material was not located. At this stage, it cannot be determined whether *Onchidium cinereum* applies to a species of *Peronia*, *Wallaconchis*, or another genus. Therefore, *Onchidium cinereum* is regarded here as a *nomen dubium*. Semper re-described *O. cinereum* based on Tonga specimens from the collections of the Museum Godeffroy (not part of the type series), and these specimens are part of a *Wallaconchis* species (based on the anatomical characters mentioned). The specimens that Semper examined indicate that a *Wallaconchis* species lives in Tonga, which could be *W. comendadori*, *W. melanesiensis*, or even a distinct species, but this could not be tested here because we did not have access to Tonga material. Finally, note that Hoffmann (1928) and Labbé (1934) commented on *O. cinereum* based on Semper’s re-description, not based on the original descriptiotion or new material.

Intra-specific genetic divergence is higher in *W. melanesiensis* than in other *Wallaconchis* species. Specimens from Vanuatu are 3.8% to 5.6% genetically divergent from the other specimens (from Indonesia and Papua New Guinea). High genetic divergences are even observed between specimens from the same locality (e.g., 3.4% within Vanuatu and Halmahera, and 4.4% within Kavieng, Papua New Guinea). The genetic divergence between the individuals from Vanuatu and those from Papua New Guinea and Indonesia could simply be an artifact of the geographic isolation of Vanuatu. Also, intra-specific divergences up to 5.5% were observed within other onchidiid species (Dayrat et al. 2016). The presence of a distinct species in Vanuatu cannot be excluded but would result in splitting *W. melanesiensis* into three or more species, as the specimens from Papua New Guinea and Indonesia are not reciprocally monophyletic with respect to Vanuatu individuals; therefore, specimens from Vanuatu are not currently considered to be a distinct species.

### Identification key

A key is provided here to help identify the ten species of *Wallaconchis*. The majority of *Wallaconchis* species cannot be distinguished externally; therefore the key is based on internal characters of reproductively mature specimens.

**Table:** 

1	The oviduct is extremely long and convoluted	***W. nangkauriense***
–	The oviduct is not extremely long and convoluted	**2**
2	The distal part of the oviduct is approximately twice as wide as the proximal part	**3**
–	The oviduct is approximately the same width along its length (narrow)	**5**
3	The penis forms loops which do not bear hooks	***W. ater***
–	The penis bears hooks	**4**
4	The penis bears hooks in two regions which are separated by a large gap without hooks	***W. graniferum***
–	Penis bears large, flattened hooks, which are distributed across the length of the penis (not separated by a large gap)	***W. uncinus***
5	Part of the oviduct forms a loop attached to the visceral cavity wall by a fibrous membrane	**6**
–	The oviduct is not attached to the visceral cavity wall	**7**
6	The penis is not protected by a penial sheath and has longitudinal ridges internally	***W. buetschlii***
–	The penis is narrow and protected within a penial sheath	***W. gracile***
7	The spermatheca is spherical	**8**
–	The spermatheca is apple-shaped or lobed	**9**
8	Oviduct is slightly wider than the deferent duct, the penis is within the base of the vestibule	***W. sinanui***
–	Oviduct is more than twice the width of the deferent duct, the penis is free within the vestibule	***W. achleitneri***
9	Deferent duct extremely convoluted	***W. comendadori***
–	Deferent duct is slightly convoluted	***W. melanesiensis***

## Discussion

### Higher relationships

The monophyly of *Wallaconchis* is highly supported by all analyses of mitochondrial and nuclear loci (Figs 1–5). Within *Wallaconchis*, clades A and B also are highly supported and are deeply divergent in molecular analyses, but there is no anatomical difference between clade A and clade B to support the creation of two distinct generic names.

### Species delineation

Ten species of *Wallaconchis* can be recognized based on internal anatomy, i.e., essentially the anatomy of the reproductive system (Table 5). However, the mitochondrial loci (COI, 12S, and 16S) support 13 molecular units while the nuclear loci (ITS1, ITS2, and 28S) support only 9 units (Figs 1–5). Molecular units in the nuclear phylogenies are largely congruent with morphology, with the exception of two species which are morphologically distinct (*W. uncinus* and *W. graniferum*) but cannot be differentiated by ITS or 28S sequences, very likely because nuclear loci are not variable enough to differentiate these two species. Six of the nuclear units are exactly the same in mitochondrial analyses and correspond to six anatomically distinct species: *W. comendadori*, *W. buetschlii*, *W. gracile*, *W. sinanui*, *W. achleitneri*, and *W. nangkauriense*.

However, there is some incongruence between mitochondrial and nuclear trees regarding three species: *W. ater*, *W. graniferum*, and *W. melanesiensis*. *Wallaconchis ater*, which forms a single unit in nuclear trees, is split in two distinct molecular units in mitochondrial trees, and the same thing goes for *W. graniferum*, and *W. melanesiensis*. For each species, the two mitochondrial units are more than 20% divergent for COI sequences (Table 2). For instance, *W. ater* unit #1 and unit #2 are from 25.7 to 28.9 % divergent with COI sequences. Now, more disturbingly, mitochondrial DNA sequences split both *W. ater* and *W. graniferum* into units which are not even closely related (*W. melanesiensis* is split into two units which are sister groups, but extremely divergent): in mitochondrial trees, *W. ater* unit #1 and unit #2 are not sister groups and *W. graniferum* unit #1 and unit #2 are not sister groups either (Figs 1–2).

If the two mitochondrial haplotypes (or units) found in each species were simply the result of a simple case of population structure, we would expect to find them to be sister groups and much less divergent from each other. In this particular case, population structure is more complex than usual. Interestingly, the extremely high mitochondrial genetic divergence observed within each of these three species (> 20 %) is accompanied by high divergence in amino acid sequences: namely, the amino acid COI sequences of *W. ater* unit #2, *W. graniferum* unit #2, and *W. melanesiensis* unit #2 are very different from those all other *Wallaconchis* species. These divergent amino acid sequences do not contain any stop codons: they are perfectly functional and are not due to a frame shift mutation. Also, all COI sequences included here most closely match other onchidiid sequences in GenBank and they are not due to contamination.

We hypothesize that the divergent COI sequences in *W. ater* unit #2, *W. graniferum* unit #2, and *W. melanesiensis* unit #2, are due to an ancestral sequence maternally transmitted through generations in three distinct lineages. It probably was present in other species as well but was ultimately lost or not sampled yet. The fact that the amino acid COI sequences of *W. ater* unit #2 and *W. graniferum* unit #2 are fairly similar to each other suggests that the ancestral haplotypes were present in a common ancestor and that the mitochondrial haplotypes that code for these amino acids are ancestral and predate more recent diversification events. The fact that the amino acid COI sequence of *W. melanesiensis* unit #2 is more closely related to *W. melanesiensis* unit #1 and *W. comendadori* is also consistent with this theory: *Wallaconchis melanesiensis* is within clade A, which diverged from clade B very early in the evolution of *Wallaconchis*. So, as the genus diversified, and new species formed, the ancestral haplotypes were only retained in a few lineages. Because both 16S and 12S evolve in the same way as COI, the trees based on concatenated mitochondrial COI, 16S, and 12S DNA sequences yielded identical results with respect to the delineation and relationships of molecular units. Overall, the delineation of *W. ater*, *W. graniferum*, and *W. melanesiensis* is therefore based on nuclear DNA sequences and morphology, and each of these three species is not split in two, as suggested by mitochondrial DNA sequences.

Integrating mitochondrial DNA, nuclear DNA, and anatomy as multiple lines of evidence has been key to delineating *Wallaconchis* species. Six out of ten *Wallaconchis* species are consistently differentiated by all three sources of data, but neither mitochondrial DNA nor nuclear DNA sequences were able to delineate all ten species. One species, *W. uncinus*, has a highly distinct penial anatomy and can be recognized using mitochondrial DNA, while nuclear ITS sequences do not distinguish it from *W. graniferum*. On the other hand, delineating *W. ater*, *W. graniferum*, and *W. melanesiensis* was possible with nuclear ITS sequences and morphology, but not possible with mitochondrial DNA.

### Species diversity

Ten *Wallaconchis* species are described here, including five new species. The five species with existing names were known only from the type material prior to the present study, and the numerous new records provided here greatly expand their geographic distribution (Fig. 6). In the secondary literature, there is only one case of non-type specimens that are known now to be part of a particular *Wallaconchis* species. *Onchidium keiense*, recognized here as a synonym of *W. ater*, was identified by Hoffmann from additional, non-type localities in the Philippines and Indonesia (Edam Island near Jakarta). Because Hoffmann is the author of *O. keiense* and its reproductive anatomy is highly distinctive, he presumably identified it correctly. Labbé identified some slugs using *Onchidium* species names transferred here to *Wallaconchis*, but those records either are clear misidentifications (e.g., see the remarks regarding *W. simrothi* in *W. nangkauriense*) or cannot be confirmed because Labbé’s material could be part of more than one *Wallaconchis* species (e.g., see the remarks on *W. buetschlii*). Thus, Hoffmann’s record of *O. keiense* in Edam Island is the only record other than the type locality which can be confirmed for the five *Wallaconchis* species with existing names.

Given that few characters can be used to classify onchidiids at the genus level and that nobody knew what those characters were before our lab started working on the revision of the entire family, it is not surprising that only through an integrative approach, could such a diverse genus be formally recognized and named. Also, the great diversity of reproductive parts in *Wallaconchis* makes it difficult to recognize that the species are related and belong to a single genus. Access to fresh material preserved for DNA analysis, combined with a broad range of data (microhabitat where each specimen was collected, color of each live animal, etc.), has been essential in revising the taxonomy of this diverse but poorly known group.

All *Wallaconchis* species are externally cryptic. No external feature can be used for species identification. However, all species are internally distinct. The high diversity of copulatory organs observed in *Wallaconchis* is unusual in the Onchidiidae. Usually, differences in the copulatory organs of closely-related species are subtle. Even when species are distinguished based on the presence or the absence of an accessory penial gland, reproductive parts are anatomically similar, as in *Melayonchis* for instance (Dayrat et al. 2017).

The geographic distribution of *Wallaconchis* species is also unique in the Onchidiidae. Many *Wallaconchis* species are sympatric in the Coral Triangle, a region with the highest diversity of reef-building corals and associated fishes (Hoeksema 2007, Veron et al. 2009), while other onchidiid genera are poorly or even not represented at all in that region (Dayrat et al. 2016, 2017, Goulding et al. in press). The geographic distribution of *Wallaconchis* species directly relates to the microhabitats that they colonized. *Wallaconchis* species are the only onchidiid species that live on coral rubble and sand (a habitat commonly encountered in the Coral Triangle because of the abundance of nearby reefs), or on rocks covered with algae and firm mud (a distinct habitat commonly found in the narrow, coastal mangroves of the Coral Triangle). These habitats are very different from the extensive and muddy mangrove forests that form around large river systems in Peninsular Malaysia, Borneo and eastern Sumatra, through the slow accumulation of large loads of silt and nutrients. Muddy mangroves are present in the Coral Triangle, but they are much smaller and less common than in the Strait of Malacca and around Borneo. The adaptation of *Wallaconchis* to coral rubble and sand on oceanic islands largely explains that its geographic distribution closely matches the hotspot of coral reef organisms (Allen 2008). It is possible that, in the future, some *Wallaconchis* species will be discovered in the South China Sea or that a species from a genus currently unknown from the Coral Triangle will be discovered there, but it is clear that the geographic distribution of *Wallaconchis* is distinct from several of the genera that live in muddy mangrove forests.

The low intra-specific genetic divergences observed in some *Wallaconchis* species suggest that high dispersal has been maintained between populations of the same species within the center of the Coral Triangle (Indonesia and the Philippines), or that populations have undergone a bottleneck and experienced a recent population expansion. Slightly higher intra-specific divergences (but still < 3.2%) tend to be observed between populations from localities on the edge of the *Wallaconchis* distribution range (Andaman Islands, Sumatra, Queensland, and Vanuatu), suggesting lower dispersal between those more isolated populations and those in the Coral Triangle. The very high mitochondrial divergence between unit #1 and #2 in *W. graniferum*, *W. ater*, and *W. melanesiensis* is likely due to the maintenance over time of ancestral haplotypes (see above). Geographic isolation may have played a role in the formation of divergent haplotypes in the early history of *Wallaconchis*, but further studies are needed to understand the evolution of these mitochondrial haplotypes.

### Nomenclature

After recognizing the existence of this genus and its ten distinct species, its unique combination of traits (no dorsal gills, intestinal loops of type I, no rectal gland, and no accessory penial gland) was used to help determine whether existing genus- and species-group names could apply to these taxa. The types of all existing onchidiid names were examined and all species descriptions were read meticulously. All existing names were considered because supra-specific relationships in onchidiids have been notoriously confusing (Dayrat 2009). Descriptions are especially important in all cases where the type material is missing or partially destroyed. Re-examining types, scrutinizing old descriptions, and comparing all characters to new samples is in its nature an extremely slow process, but this time-consuming detective work is vital to sound taxonomy. Without systematically addressing all of the existing onchidiid species-group names, we would not have been able to find and save so many existing names in *Wallaconchis*.

The confusing nomenclature of the Onchidiidae has clearly hindered biodiversity knowledge. The fact that all existing names had to be considered before any new species could be described has kept taxonomists away from the Onchidiidae for more than 80 years (Dayrat 2009). As a result, several species of large slugs with a completely distinct anatomy and commonly found in rocky intertidal were never described. Also, in cases where existing names were found to apply to some of the large, colorful, and common species described here, it remains that these species were poorly known since their original descriptions. Thanks to the many new records provided here, these species are now known in much greater detail, anatomically, ecologically, and biogeographically.

### Diversification: transitions to vastly different microhabitats

The fact that *Wallaconchis* species live in different microhabitats is unique among the onchidiids. In other onchidiid genera, all species are found in the same microhabitat(s). For example, *Onchidium* species live in mangroves, where they are found on (or inside) old logs and on mud; *Melayonchis* species are found in mangroves, on tree roots and trunks as well as old logs; *Peronina* species are found in mangroves, on mud saturated with water; and *Peronia* species are found on rocks in the rocky intertidal. The different microhabitats on which *Wallaconchis* species live are frequently in close proximity to coastal mangroves, and it was only by photographing many specimens in the field that we were able to determine that the species were found on different substrates. The adaptation of *Wallaconchis* species to different microhabitats is perfectly illustrated by our station 198, on the southern coast of Bohol, Philippines, where an ideal combination of microhabitats (coral rubble, fine sand, and sandy mud) brought all five *Wallaconchis* species from clade B present in the Philippines (there are three other species in clade B but they are not found in the Philippines) to a single site of only 100 square meters (Fig. 43A). Additionally, *Wallaconchis* was the only genus of onchidiids present at that site, illustrating that coral rubble and sandy mangroves are microhabitats to which only *Wallaconchis* became adapted. Importantly, *W. comendadori* (clade A), which is also present in the Philippines but lives on rocks covered by algae, was not present at the site, but was found on a site with some coral rubble and a rocky shore at our station 197, also on the southern coast of Bohol, illustrating that the presence of those species depends on distinct microhabitats.

The adaptation of *Wallaconchis* species to distinct microhabitats likely is essential to the existence of so many species at the same sites, which would otherwise compete for limited food resources. In other onchidiid genera, species live in the same microhabitat and thus probably utilize the same resources. Ultimately, this may largely explain the distinct patterns of species richness observed in *Wallaconchis* in comparison to the two other onchidiid genera with a similar number of species (*Peronia* and *Platevindex*). Indeed, in *Peronia* and *Platevindex*, species richness peaks at three or four species within any given region, while eight *Wallaconchis* species are sympatric and broadly distributed in the Coral Triangle (Fig. 6). In other genera, species potentially live in the same places (because they are adapted to the same microhabitat), while, strictly speaking, many *Wallaconchis* do not live in the same places even though they are sympatric geographically, because they are adapted to distinct microhabitats.

**Figure 66.**
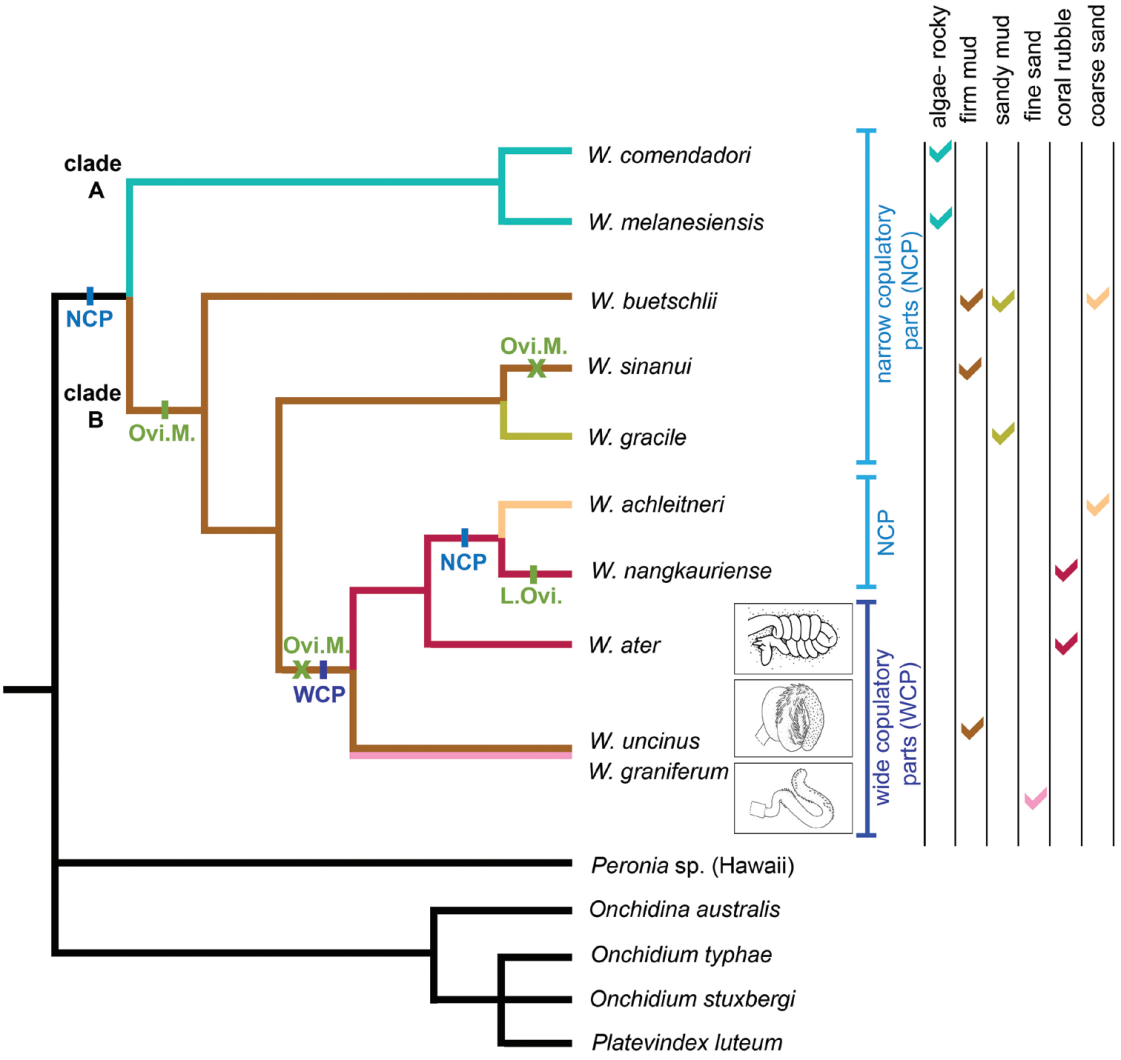
Phylogenetic relationships of *Wallaconchis* species, with shifts in habitat and character state mapped (for the details on node support and numbers of specimens included in each species, see Figs 1–5). Abbreviations L.Ovi. long oviduct NCP narrow copulatory parts Ovi.M. oviduct membrane WCP wide copulatory parts.

Microhabitat adaptation can be mapped onto our *Wallaconchis* phylogenetic tree, to form hypotheses about the diversification processes and microhabitat shifts that possibly took place during the evolutionary history of *Wallaconchis* (Fig. 66). The deep divergence between the two clades of *Wallaconchis* (clades A and B) was accompanied by a colonization of a distinct microhabitat. Species in clade A live on rocks covered by algal mats while the basal species in clade B live on mud, sandy mud, or coarse-grained sand. It is still unclear what microhabitat was ancestral to all *Wallaconchis*. Given that most onchidiids live in mangroves, it is likely that the slugs of the ancestral lineage of *Wallaconchis* lived on mud, like the most basal species in clade B (*W. buetschlii*, Fig. 66). However, if it is shown later that *Peronia* is sister-group to *Wallaconchis*, then a rocky habitat could be ancestral to all *Wallaconchis* because all *Peronia* species live in the rocky intertidal. In clade B, it is possible that the ancestral lineage was a generalist, as the most basal species, *W. buetschlii*, today is the only *Wallaconchis* adapted to three distinct microhabitats –firm mud, muddy sand, and coarse-grained sand (Fig. 66). Early branches of clade B then specialized to sandy mud (*W. gracile*) and firm mud (*W. sinanui*). After the divergence of *W. sinanui* and *W. gracile*, lineages split and then transitioned to new microhabitats. *Wallaconchis uncinus* remained on muddy or coarse-grained sand, while *W. graniferum* shifted onto fine sand. *Wallaconchis uncinus* and *W. graniferum* diverged from a lineage that likely transitioned into coral rubble habitats before diversifying in *W. ater*, *W. nangkauriense*, and *W. achleitneri*. That would suggest that the species *W. achleitneri* transitioned back to a sandy-muddy habitat, although it could also be the remnant of an ancestral habitat. However, it is also possible that *W. ater* and *W. nangkauriense* represent independent transitions to coral rubble habitats from a muddy habitat (the phylogenies suggest that they are not sister taxa, see Figs 1–5).

*Wallaconchis* is among the most diverse genera of onchidiids in the tropical Indo-West Pacific (*Peronia* and *Platevindex* being slightly more diverse), and the ability of *Wallaconchis* lineages to colonize new microhabitats has likely been instrumental in its diversification. The high diversity in *Wallaconchis* is especially remarkable considering that *Peronia* is distributed much more widely (from South Africa and the Red Sea all the way to Hawaii and French Polynesia). However, unlike *Wallaconchis*, *Peronia* is restricted to a single habitat (rocky intertidal) and its diversification must have been driven by other factors than transitions to new habitats.

### Diversification: the role of reproductive isolation

The diversity of reproductive parts (especially the male copulatory ones) in *Wallaconchis* is truly exceptional in comparison to other onchidiids. In other genera, species occasionally differ with respect to the presence or absence of penial hooks or of an accessory penial gland, but otherwise the morphology of the copulatory organs is generally similar. All *Wallaconchis* species can be distinguished using reproductive parts, and the highly distinct penial hooks and loops found in some *Wallaconchis* species have not been observed in any other genera.

Mapping the different reproductive parts onto the *Wallaconchis* phylogenetic tree shows that the ancestral state likely was a narrow penis and a narrow oviduct, because it is shared by all species in clade A and the three most basal species in clade B (Fig. 66). In clade B, a wider penis and a correspondingly thicker oviduct were acquired (shared by *W. uncinus*, *W. ater*, and *W. graniferum*) and then lost again in the lineage ancestral to *W. nangkauriense* and *W. achleitneri* (Fig. 66). Not all wide copulatory parts are the same. There are three distinct types: a tightly coiled penis with no hooks (in *W. ater*), a coiled penis with flat hooks (in *W. uncinus*), and a penis with regions of hooks separated by a gap without hooks (in *W. graniferum*). The diversity of reproductive parts in *Wallaconchis* suggests that reproductive isolation has played an important role in diversification. Even though *Wallaconchis* species are specialized to different microhabitats, these microhabitats are often in close proximity. So, some incompatibility between reproductive parts may have contributed to the continued co-existence of these species by preventing hybridization.

### Endemism and conservation

Most *Wallaconchis* species are common. However, *W. sinanui* and *W. achleitneri* were each found at only one site and may be micro-endemic. It would have been impossible to discover such rare and endemic species without extensive sampling (gastropods were collected at more than 260 stations across the Indo-West Pacific, especially in South-East Asia). Now that *Wallaconchis* is well delineated and characterized and all ten known species have been described with modern tools, additional species will likely be discovered.

The majority of *Wallaconchis* species, like most other onchidiid species (Cumming et al. 2014, Dayrat et al. 2016, 2017), are broadly distributed. Those species are often threatened locally by habitat destruction or pollution, but their large distribution helps protect them from any imminent extinction. In contrast, the two micro-endemic species *W. sinanui* and *W. achleitneri* are likely to be much more vulnerable and susceptible to extinction. With additional fieldwork, rare or narrowly endemic species may be found at additional localities, but they are unlikely to be common. We found some endemic species in other onchidiid genera, as in *Peronina* (Goulding et al. in press); however, these endemic species were found at several sites. In *Wallaconchis*, the two endemic species appear to need a highly specific substrate or diet because they were each found at only one site (and in both cases they were very abundant at that site). Further study is needed locally to determine whether *W. sinanui* and *W. achleitneri* are present at other sites nearby their type locality, and if the habitats where they were found need any special protection.

## Supplementary Material

XML Treatment for Wallaconchis

XML Treatment for Wallaconchis sinanui

XML Treatment for Wallaconchis uncinus

XML Treatment for Wallaconchis buetschlii

XML Treatment for Wallaconchis gracile

XML Treatment for Wallaconchis nangkauriense

XML Treatment for Wallaconchis ater

XML Treatment for Wallaconchis graniferum

XML Treatment for Wallaconchis achleitneri

XML Treatment for Wallaconchis comendadori

XML Treatment for Wallaconchis melanesiensis
